# Long-term outcomes of a digital alcohol intervention targeting online help-seekers: a simulation study of incidence of disease, quality-adjusted life-years and costs

**DOI:** 10.1136/bmjph-2025-003503

**Published:** 2026-06-24

**Authors:** Katarina Ulfsdotter Gunnarsson, Martin Henriksson, Marcus Bendtsen

**Affiliations:** 1 Department of Health, Medicine and Caring Sciences, Linköping University, Linköping, Sweden

**Keywords:** Public Health, Epidemiologic Methods, Digital Health, Epidemiologic Factors, Program Evaluation

## Abstract

**Introduction:**

Harmful and hazardous alcohol consumption is a major public health concern and places considerable burdens on societies. Digital alcohol interventions (DAIs) are promising tools to support behaviour change, yet the extent to which their behavioural effects translate into long-term health benefits is unclear. This study aimed to develop and apply an individual-level simulation model to estimate the long-term outcomes of a DAI.

**Methods:**

We developed an individual-level simulation model to simulate the life course of individuals in two virtual cohorts: one receiving a DAI cohort and the other receiving treatment-as-usual (TAU cohort). We contrasted incidence rates for ten alcohol-related diseases, quality-adjusted life-years (QALYs) and healthcare costs between the cohorts. We also calculated an incremental cost-effectiveness ratio.

**Results:**

The results indicated that when assuming a sustained population-level intervention effect over time, the DAI cohort experienced lower incidence rates of most alcohol-related diseases compared with the TAU cohort. The intervention also yielded a marginally higher per individual QALY (0.008, IQR=−0.007; 0.023) compared with treatment as usual, as well as a lower cost (€−89, IQR=€−147; €−30). When the population-level effect was assumed halved or slowly waning over time the results were similar but attenuated, and when assumed to wane more rapidly, there were no marked differences between cohorts. While the digital intervention could be considered a dominant strategy, considerable uncertainty remained in the cost-effectiveness plane when exploring varying assumptions about the effectiveness.

**Conclusions:**

DAIs could play a role in a comprehensive public health strategy aimed at reducing alcohol consumption. However, challenges remain to produce stronger evidence for their cost-effectiveness, not least addressing the lack of data on persistent effects. The realisation of these benefits in practice depends on effective implementation, including sustained investment in dissemination and monitoring of their use.

**WHAT IS ALREADY KNOWN ON THIS TOPIC**

- Harmful alcohol consumption is a major public health issue, and digital interventions have demonstrated short-term effectiveness in reducing drinking.
- The long-term health and economic impacts of digital alcohol interventions remain largely unexplored.

**WHAT THIS STUDY ADDS**

- By developing and using an individual-level simulation model, this study demonstrates that a digital alcohol intervention has the potential to reduce alcohol-related diseases and increase quality-adjusted life-years at a lower cost compared with treatment-as-usual; however, there are considerable uncertainties attached to the findings.

**HOW THIS STUDY MIGHT AFFECT RESEARCH, PRACTICE OR POLICY**

- Digital alcohol interventions could be integrated into public health strategies to mitigate alcohol-related harm, yet stronger evidence is necessary to better understand the implications of doing so.
- Future research should look to mitigate challenges in modelling long-term outcomes and include a societal perspective.

## Introduction

 Harmful and hazardous alcohol consumption remains a pervasive public health concern globally, with significant social, economic and health-related consequences.^1^,^3^ In Sweden, approximately one-third of men and one out of six women, are harmful users of alcohol according to national guidelines which take into consideration both total consumption and frequency of episodes of heavy drinking.^4^ It has been estimated that the societal costs related to alcohol harm amounts to €9 billion annually in Sweden, with the healthcare sector alone accounting for 15% of this cost.^5^

One form of prevention which has emerged over the past decade is the dissemination of digital alcohol interventions (DAIs)—interventions which deliver supportive content to individuals who could benefit from reducing their alcohol consumption through, for instance, websites, mobile phone applications, text messages and email. DAIs significantly expand the reach of traditional brief interventions by leveraging technology that is almost ubiquitous in many countries, including Sweden, and can overcome barriers of stigma associated with seeking support. Digital interventions also allow for personalised support,^6 7^ typically combining brief, low-intensity modules built on established behaviour-change approaches. These most commonly consist of self-monitoring with personalised normative feedback, motivational interviewing approaches, cognitive-behavioural therapy and behavioural self-control training. Overall, DAIs can reach those seeking help outside conventional care and have, in meta-analysis, been found to yield reductions in population level alcohol use (~22 g ethanol/week) and higher adherence to low-risk guidelines.^6 7^

Negative consequences from alcohol consumption develop over time; thus, it is necessary to adopt a long-term perspective when evaluating prevention interventions. If not, evaluations of these types of interventions on a short-term basis lead to an underestimation of the expected health outcomes, underestimation of costs and do not provide suitable guidance for decision-making regarding dissemination.^8 9^ However, in the existing literature, studies aiming to estimate the cost-effectiveness of DAIs have overlooked the opportunity to leverage modelling techniques capable of studying long-term outcomes appropriately over extended time horizons.^10^ Incorporating such modelling approaches is essential to provide better guidance regarding expected future outcomes of DAIs.

In a recent trial, the effectiveness of a DAI was estimated among Swedish adults who were looking online for help to reduce their drinking.^11 12^ The core component of the digital intervention was a weekly text reminder sent to participants on Sunday afternoons. The text invited participants to reflect on their past week’s consumption by completing a self-monitoring and feedback tool. After having received feedback on their consumption, participants were given access to interactive support tools, including normative feedback, information about risks, timeline and goal setting and skill-building. The support tool also offered optional supportive text messages sent to participants throughout the week. For a more detailed description of the intervention, please see the trial protocol and presentation of main findings.^11^ The intervention was contrasted with a control group who were given information about alcohol and health typically found when searching online. Four months post-randomisation, the intervention group reported a weekly consumption that was approximately 77% of the amount of alcohol that the control group were drinking.^12^ These results were encouraging since a population level reduction in alcohol consumption should lessen its burden on society. However, while the effects on behaviour could be estimated within the conditions of the trial, the degree to which these effects impact long-term health outcomes remains uncertain^10^—thus, the degree to which the digital intervention can reduce the burden on society caused by alcohol is unknown. Therefore, the aim of this study was to develop and apply an individual-level simulation model capable of estimating the long-term health outcomes of the DAI, specifically in terms of incidence of alcohol-related diseases, quality-adjusted life-years (QALY) and healthcare costs.

## Methods

In this study, we developed and applied an individual-level simulation model to estimate the long-term outcomes of a DAI. Unlike traditional cohort state-transition models that track average risks within broad health states, individual-level simulation represents person-level heterogeneity (age, sex, baseline consumption), accounts for history-dependent processes by updating each individual’s attributes over time, and allows coexisting conditions that affect risks, costs and QoL.

In online supplemental appendix A, we have provided a detailed description of the model structure, analytical assumptions, how the model is parameterised, and validation results showing how outcomes match against expected incidence of disease and mortality. The model operated by simulating the life course of individuals, and we used a lifetime time horizon to account for the potential of preventing long-term morbidity and reducing premature mortality. The simulated individuals made up two virtual cohorts: one cohort was administered the DAI cohort, while the other cohort did not have access to the intervention (treatment-as-usual (TAU cohort)). The estimate of relative difference in total weekly alcohol consumption between these two cohorts on alcohol consumption was based on results from a previous trial of the digital intervention (incidence rate ratio=0.77, 95% compatibility interval=0.69;0.86, probability of effect >99.9%).^11 12^

After simulating the life course of the two cohorts we contrasted health-related outcomes, thus allowing us to study the long-term outcomes attributed to the DAI. By using Monte Carlo simulations, the results presented incorporate the uncertainty attached to the various input parameters presented in online supplemental appendix A. Modelling and data analysis were conducted in accordance with Consolidated Health Economic Evaluation Reporting Standards.^13^ The simulation model was built using Python and analyses were conducted using Python (pandas and numpy) and R V.4.2.

### Population characteristics and initialising cohorts

We intended to simulate the life course of a population which reflected those participating in the trial of the DAI.^12^ The trial was designed to mimic a real-world implementation, with low barriers for participation by not requiring face-to-face contact and ensuring confidentiality, as well as using technology ubiquitous in Sweden. Additionally, participants in the trial had searched online for help and so we overall considered them to be similar to those who would look for and use the intervention when disseminated. To reflect the participants in the trial, the simulated population consisted of 58% women and 42% men, and the mean age of the individuals was 46 years (quartiles=37; 54). We created two cohorts that were identical at baseline (the DIA and TAU cohorts).

Assignment of individual level weekly alcohol consumption was done as follows. First, we sampled a weekly alcohol consumption for each individual using a negative binomial model conditional on their age and sex to match the distribution of alcohol consumption among trial participants when they entered the study. This represented the baseline consumption that participants entered the study with. Then, at the start of each simulation, we altered the individuals’ alcohol consumption relative to the estimated conditional effect of the intervention (TAU cohort) or control conditions (DAI cohort) in the trial. These relative effects were drawn from a negative binomial model that was adjusted for age, sex and baseline consumption; thus, we retained both individual response to the conditions and the uncertainty of the estimated effects. On average, women in the DAI cohort consumed 92 g of alcohol per week, while women in the TAU cohort consumed 117 g. The corresponding numbers for men were 125 g in the DAI cohort and 158 g in the TAU cohort.

### Input parameters and outcomes

We modelled morbidity and mortality based on several existing data sources (for further information see online supplemental appendix A). When available, we sought input parameters which were age and sex specific. The probability of being diagnosed with a disease was determined based on incidence rates published by the Swedish National Board of Health and Welfare. Disease-specific mortality rates were derived from the Swedish National Board of Health and Welfare’s Cause of Death register, and non-disease specific mortality ratios were taken from the standard mortality ratio published by Statistics Sweden.

We derived HRs associated with weekly alcohol consumption from published research literature. In the literature, HRs are sometimes reported as a HR per different category of amount of alcohol consumed, for edxample, an HR for those consuming between 140 and 280 g of alcohol per week. In these cases, in order to obtain a continuous relationship between quantity and risk, we estimated a new HR per gram of alcohol consumed, based on the HRs reported per category. We did this using natural cubic spline models that captured the non-linear relationships between grams of alcohol consumed and HRs; thus, we captured curved relationships and did not assume linear relationships between alcohol consumption and relative risks. For stroke and myocardial infarction, HR estimates in the literature were already provided on continuous scale (per 100 g of alcohol), which meant that we did not need to re-estimate spline models but could use these directly. Since these were estimated in log-space and transformed to HRs, they also provided non-linear relationships between consumption and hazard ratios (see online supplemental appendix A).

The simulation model included 10 diseases (see table 1), including cardiovascular diseases, alcohol-related liver disease and six different types of cancer. The diseases were chosen based on alcohol consumption having an impact on their incidence. Quality of Life (QoL)-weights and costs (converted to 2024 euros using the consumer price index) associated with each disease were collected from the literature. We did not model treatment-specific effects on QoL or costs associated with diseases, ie, the intervention only impacted the incidence of diseases not their severity (see online supplemental appendix A).

**Table 1:** Alcohol-related diseases included in the simulation model

Diseases (ICD-10 codes*)	Reference
Alcohol-related liver disease (K70, K73-K74)	^21^
Breast cancer (C50)	^22^
Colorectum (C18–20)	^22^
Oesophageal cancer (C15)	^22^
Haemorrhagic stroke (I60–62)	^23^
Ischaemic stroke (I63–67)	^23^
Myocardial infarction (I21)	^24^
Liver cancer (C22)	^21^
Oral cancer (C00-06, 09–10, 12–14)	^22^
Pancreas cancer (C25)	^22^

* International Classification of Diseases, 10th Revision

We contrasted incidence rates, QALYs, healthcare costs, and mortality between the two virtual cohorts. We also calculated an incremental cost-effectiveness ratio (ICER) to establish dominance (DAI associated with higher QALYs and reduced costs compared with TAU). HRs for disease incidence and mortality were calculated using proportional hazards regression, with only the first incidence counted per individual. Incremental costs and incremental QALYs from all simulations were presented in cost-effectiveness planes.

Cancer diseases were modelled to develop at maximum once in an individual (co-morbid cancer diseases were allowed). Breast cancer could progress contralaterally and recur. Increased mortality risk and risk of progression or recurrence were accounted for in the first 5 years after diagnosis. However, for some cancer types, cost and QoL continued to be affected through life. Cancers were modelled to be detected in different stages which affect cost and QoL, please see online supplemental appendix A.

The increased mortality risk associated with stroke (haemorrhagic and ischaemic) and myocardial infarction only applied during the first year, but incidence led to a reduced QoL for the rest of life. These diseases could recur any number of times. Stroke recurrence was also higher among individuals aged above 65, with HRs increasing with age, please see online supplemental appendix A.

Individuals diagnosed with alcohol-related liver disease experienced an increased risk of mortality for the rest of life, as well as an increased cost and reduction in QoL. Alcohol-related liver disease could progress into liver cancer, in which case the increased risk of mortality from liver cancer was used rather than alcohol-related liver disease, and similarly so for costs and QoL. If the individual survived liver cancer (after 5 years), then mortality, cost, and QoL were again based on alcohol-related liver disease.

### Scenarios

To investigate the importance of modelling assumptions for the results, we ran simulations under four scenarios. In the first scenario, the population level effect of the DAI remained constant throughout the simulation. In other words, while alcohol consumption may fluctuate at the individual level, the expected difference between groups was assumed to remain at the population level. For scenario 2, we assumed that the interventions’ population level effect disappeared entirely after 10 years, with a linear annual reduction of 10 percentage points, and for scenario 3, we assumed that the intervention’s population level effect waned by 1 percentage point per year. Finally, in scenario 4, we assumed a constant population level effect as in scenario 1 but halved the effect size of the intervention to provide results under the assumption that the intervention’s effectiveness may not be generalisable as estimated. The inclusion of these scenarios also reflects that evidence for how the effects of DAIs persist over the long term is lacking in the research field.

In line with current recommendations, both QALYs and costs were discounted at an annual rate of 3%. We also explored various discounting assumptions. Specifically, we conducted simulations using discount rates of 0% for costs and QALYs; 5% for costs and QALYs and 3% for costs and 0% for QALYs.^14^ Additionally, to assess cost-effectiveness across age groups, we simulated the distribution of ICER values with QALY discounting (3%) and no QALY discounting. For each age group, ICER distributions were generated and compared with examine how discounting assumptions influence the relative cost-effectiveness of intervening at different ages.

## Results

**Figure 1:**
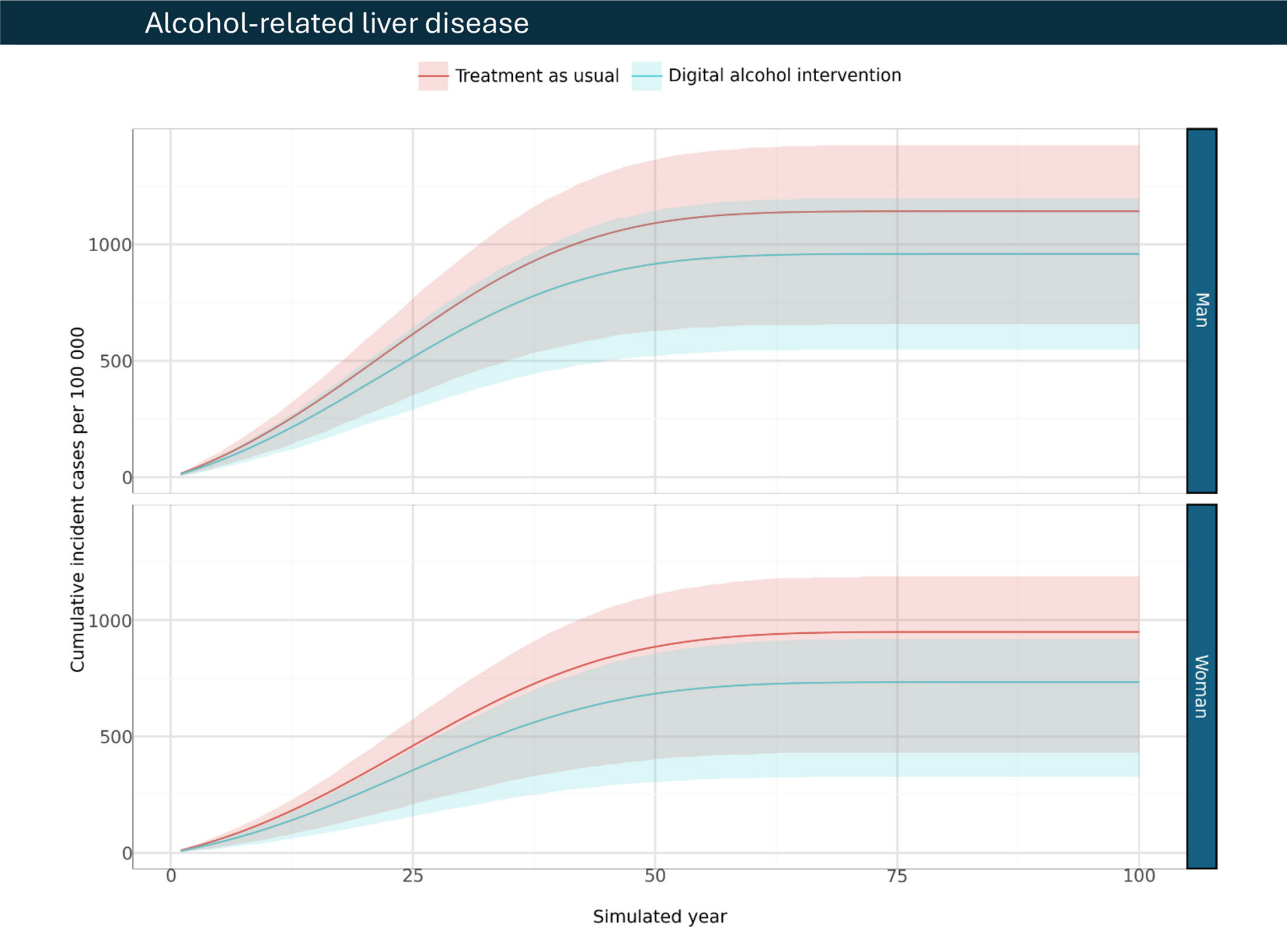
Cumulative incidence rates per 100 000 individuals of alcohol-related liver disease (mean and IQR representing the uncertainty across simulations).

The results from simulations of the first scenario, where the difference in population-level alcohol consumption remained constant between the two cohorts, are presented in table 2. We found that the intervention was associated with a lower incidence rate for most of the modelled diseases. For example, the number of cases of alcohol-related liver disease per 100 000 individuals in the DIA cohort was 960 (IQR=549; 1197) for men and 735 (IQR=327; 913) for women, compared with 1145 (IQR=663; 1431) for men and 959 (IQR=434; 1192) for women in the TAU cohort. The estimated HR for alcohol-related liver disease associated with the DAI was 0.838 (95% CI 0.836 to 0.841) for men and 0.773 (95% CI 0.771 to 0.775) for women. Note that the HR is based on the first incidence per-individual and does not consider recurrence. In figure 1, we have plotted the mean and IQR cumulative incidence of alcohol-related liver disease per 100 000 over the simulated cycles for both men and women. As can be seen, the DAI cohort and TAU cohort diverge early in the simulation, and there is a distinction between the two cohorts in terms of incidence rates. Online supplemental file 3Appendix C, Panels B to Jonline supplemental figure 1 shows the mean and IQR cumulative incidence rates for the other modelled diseases. We found no evidence of a difference in the hazard rate for mortality between the two cohorts. Regarding myocardial infarction and pancreatic cancer, the DAI was associated with a higher incidence of cases. This aligns with our input data, which suggested that these conditions may be partially protected by higher levels of alcohol intake. However, taken together with findings for other diseases, these potential harms from the intervention seem negligible.

**Table 2:** Long-term simulation outcomes, intervention effect constant over time (scenario 1)

Scenario 1: intervention effect constant over time								
	Incidence rates, mean (IQR)*					**HR (95% CI)†**		
	**DAI**		**TAU**			**DAI vs TAU**		
**Number of cases per 100 000 individuals:**	**Men**	**Women**	**Men**		**Women**	**Men**		**Women**
Alcohol-related liver disease	960 (549 to 1202)	735 (327 to 913)	1145 (663 to 1431)		949 (434 to 1192)	0.838 (0.836 to 0.841)		0.773 (0.771 to 0.775)
Breast cancer	21 (14 to 29)	3195 (3014 to 3369)	21 (14 to 29)		3246 (3059 to 3427)	-- ^‡^		0.984 (0.983 to 0.985)
Colorectum cancer	1507 (1421 to 1588)	1341 (1267 to 1409)	1537 (1445 to 1617)		1368 (1299 to 1436)	0.981 (0.979 to 0.983)		0.980 (0.978 to 0.982)
Oesophageal cancer	183 (143 to 215)	71 (55 to 86)	191 (152 to 224)		73 (55 to 86)	0.960 (0.951 to 0.963)		0.975 (0.966 to 0.983)
Haemorrhagic stroke	1112 (1062 to 1164)	897 (858 to 933)	1203 (1149 to 1254)		947 (906 to 989)	0.924 (0.922 to 0.927)		0.947 (0.945 to 0.949)
Ischaemic stroke	5930 (5795 to 6057)	4860 (4757 to 4960)	6276 (6139 to 6415)		5061 (4953 to 5162)	0.947 (0.946 to 0.948)		0.961 (0.960 to 0.962)
Liver cancer	600 (444 to 720)	346 (227 to 410)	671 (496 to 806)		406 (265 to 482)	0.839 (0.891 to 0.897)		0.851 (0.848 to 0.855)
Myocardial infarction	5021 (4913 to 5123)	3211 (3138 to 3286)	4910 (4808 to 5013)		3153 (3079 to 3224)	1.022 (1.020 to 1.023)		1.018 (1.017 to 1.020)
Oral cancer	308 (258 to 348)	174 (148 to 200)	329 (277 to 372)		184 (155 to 210)	0.936 (0.932 to 0.941)		0.946 (0.941 to 0.952)
Pancreas cancer	290 (262 to 320)	295 (269 to 324)	287 (258 to 315)		295 (265 to 320)	1.007 (1.002;1.013)		1.001 (0.999 to 1.005)
	Cost-effectiveness outcomes (per individual), mean (IQR)*							
	**DAI**			**TAU**			**Difference**	
Healthcare costs (euro)	2977 (2870 to 3058)			3065 (2947 to 3155)			−89 (−147 to −30)	
QALY	19.673 (19.656 to 19.691)			19.665 (19.648 to 19.683)			0.008 (−0.007 to 0.023)	
ICER (euro per QALY)	DAI dominant (improved QALYs and lowered costs)							

* IQR represents the uncertainty across simulations

† CI, mean plus/minus 1.96 SEs.

‡ Estimating HR was not feasible due to an insufficient number of cases of breast cancer among men

DAI, digital alcohol intervention; ICER, incremental cost-effectiveness ratio; QALY, quality-adjusted life-years; TAU, treatment-as-usual.

Table 2 also contains estimates for QALY, cost and ICER. The intervention did yield a marginally higher per individual QALY (0.008, IQR=−0.007; 0.023) compared with treatment as usual, as well as a lower cost (€−89, IQR = €−147; €−30). Consequently, the digital intervention can be considered a dominant strategy.

Appendix B online supplemental figure 1 presents a cost-effectiveness plane, showing that 53% of simulations fell within the southeast (SE) quadrant and 31.7% within the southwest (SW) quadrant. This suggests that, while the digital intervention is likely to be both more effective and less costly than TAU, considerable uncertainty remains as a large proportion of simulations resulted in lower effectiveness alongside reduced costs. A smaller proportion of simulations fell in the northwest (NW) quadrant (5.5%), while 9.8% lie in the northeast (NE) quadrant.

### Reducing the population level effect of the intervention

The results from simulations of the second, third, and fourth scenarios showed similar patterns as for the first scenario, where fewer incident cases were found in the DAI cohort compared with the TAU cohort for most diseases (see appendix B online supplemental tables 1-3). In the second scenario, where the population level effect of the intervention was reduced by 10 percentage points annually, the HRs were sharply pulled towards the null. However, in the third scenario, where the population level effect of the intervention was reduced by 1 percentage point annually, a clear distinction in hazard rates remained. For example, in the second scenario, the HR for alcohol-related liver disease for men was 0.985 (95% CI 0.982 to 0.987) and for women 0.982 (95% CI 0.980 to 0.985) compared with scenario three, where the HR for men was 0.873 (95% CI 0.870 to 0.875) and for women 0.823 (95% CI 0.821 to 0.825). Similar to the third scenario, when halving the intervention’s effect (fourth scenario), the estimates were pulled towards the null but with distinctions between incidence rates still clear for diseases where the risk profile with alcohol is strong.

Due to the different assumptions about the intervention’s effect size in the scenarios, the resulting difference in disease HRs also resulted in differences in cost-effectiveness results. In the second scenario, the simulations almost fell uniformly in the quadrants of the cost-effectiveness plane (see online supplemental figure 2), questioning the value of implementing the digital intervention. In the third scenario, the quadrant distribution was similar to the first scenario, while in the fourth scenario it was more even between the SW and SE quadrants (appendix B online supplementary figure 3 and figure 4). Notably, these results show the inherent uncertainty in forming conclusions from this study due to the lack of studies of the persistence of effects of DAIs.

### Discounting

In table 3, QALYs, costs and cost-effectiveness resulting from simulations where discount rates were altered are presented. As expected, a lower discount rate yielded higher QALYs and increased costs, while a higher discount rate resulted in the opposite. The most significant change in the results occurred when QALYs and costs were discounted at different rates, making the digital intervention save more healthcare costs and gain more QALYs compared with when both were discounted at 3%.

**Table 3:** Cost-effectiveness outcomes and various discounting assumptions

Cost-effectiveness outcomes (per individual), mean (IQR)*						
	**QALY and cost discounted at 0%**		**QALY and cost discounted at 5%**		**QALY discounted at 0% and cost discounted at 3%**	
	**DAI**	**TAU**	**DAI**	**TAU**	**DAI**	**TAU**
Healthcare costs (euro)	7417 (7156; 7626)	7622 (7610; 7634)	1775 (1712; 1824)	1827 (1756; 1883)	2976 (2872; 3059)	3064 (2945; 3158)
Difference	−207 (−345; −58)		−53 (−91; −14)		−88 (−148; −25)	
QALY	34.441 (34.398; 34.487)	34.421 (34.379; 34.467)	14.666 (14.656; 14.676)	14.661 (14.651; 14.672)	34.441 (34.398; 34.486)	34.421 (34.377; 34.468)
Difference	0.020 (−0.018; 0.056)		0.005 (−0.005; 0.014)		0.020 (−0.017; 0.056)	
ICER (euro per QALY)	DAI dominant		DAI dominant		DAI dominant	

* IQR represents the uncertainty across simulations

DAI, digital alcohol intervention; ICER, incremental cost-effectiveness ratio; QALY, quality-adjusted life-years; TAU, treatment-as-usual.

**Figure 2:**
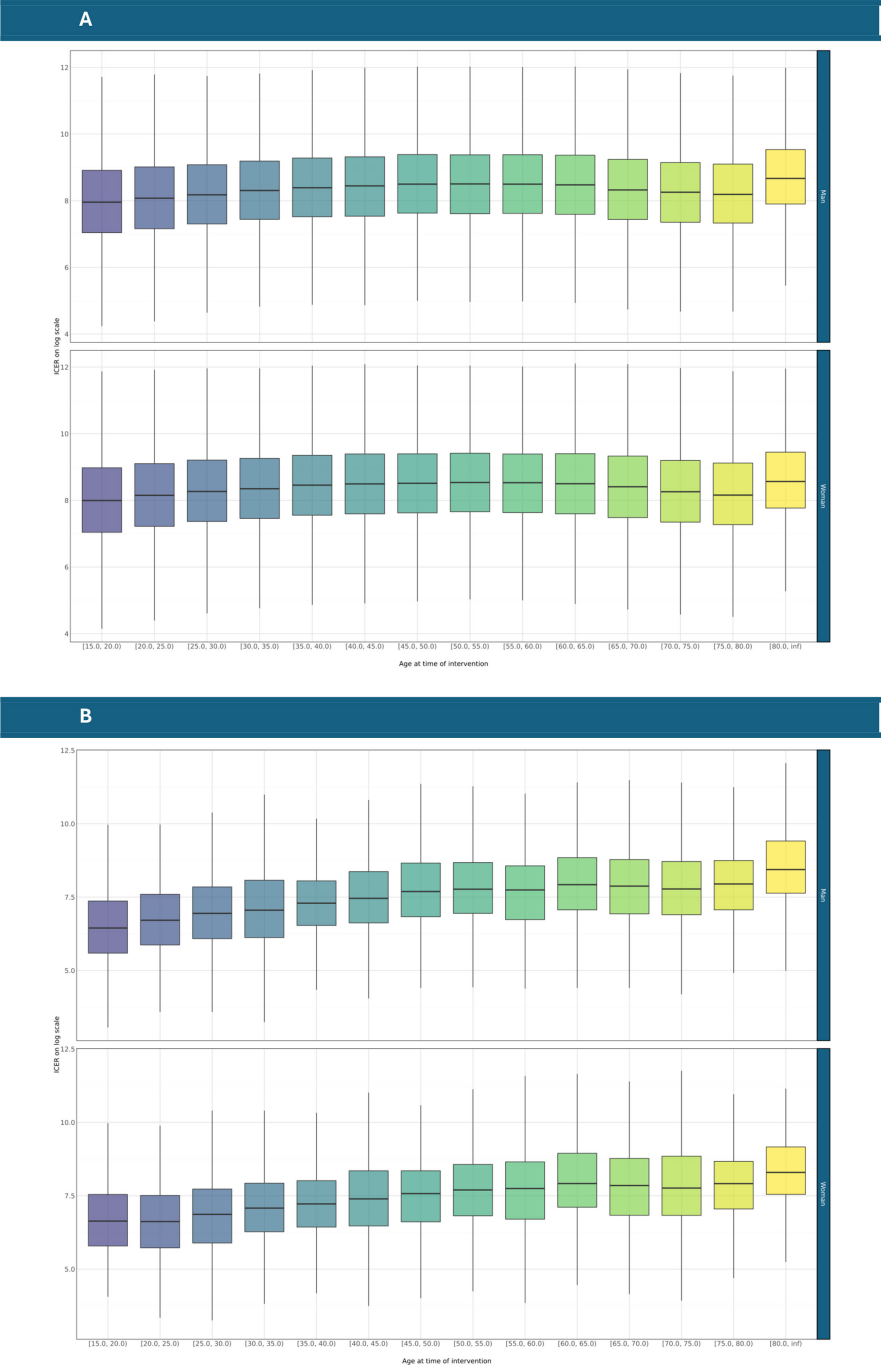
Distribution of ICER on the log scale conditional on age groups at the time of intervention. (A) Results from simulations where QALY discount rate was 3%. (B) Results from simulations when there was no discounting of QALY. ICER, incremental cost-effectiveness ratio; QALY, quality-adjusted life-years.

In figure 2, distributions of ICER (on the log scale) for different age groups are presented. A shows how the ICER is distributed under default discounting of QALYs (3%). There are differences between age groups, as expected, intervening in younger adults is likely to be more cost-effective. However, intervening in the upper age groups is also more cost-effective than the middle-age groups, possibly due to these groups having a much higher baseline risk for non-communicable diseases. Figure 2B shows the same analysis but without discounting QALYs. In this case, it is clearer that intervening early is more cost-effective.

## Discussion

We developed an individual-level simulation model to contrast two virtual cohorts to study the long-term outcomes of a DAI compared with TAU on incidence of disease, QALY, and healthcare costs. We used a lifetime time horizon to capture the potential of the intervention to prevent morbidity in the long-term. We also created four scenarios in which we tested different assumptions about the duration of the intervention effect. Under the assumption that the intervention effect is constant over time, the results showed that there was a lower incidence of disease in the DAI cohort that also spilled over to a reduction in healthcare costs. In cost-effectiveness terms this implies that DAI is a dominant strategy as it is associated with a gain in QALYs at lower costs compared with TAU. However, significant uncertainties and challenges remain that should be borne in mind when interpreting these findings, not least due to the lack of evidence to support the assumptions of persistent effects over time. The uncertainty that is portrayed in the cost-effectiveness plane, with 53% of simulations falling in the SE quadrant (higher effectiveness, lower costs) and 31.7% in the SW quadrant (lower effectiveness, lower costs), is also an indication of the uncertainty that remains regarding the cost-effectiveness of the intervention studied. The consequences from the uncertainty regarding the persistence of effects over time is illustrated in the cost-effectiveness plane for the three other scenarios explored. For example, when we assumed that the intervention’s effect disappeared entirely after 10 years (scenario 2), the simulated outcomes were almost uniformly distributed across all four quadrants of the cost-effectiveness plane, indicating no clear tendency toward either dominance or inferiority. In scenarios 3 (intervention effect declining by 1% per year) and scenario 4 (50% reduction in effect size), the cost-effectiveness planes were more similar to the plane in scenario 1. Here, 31.2% and 30.4% of simulations fell in the SW quadrant, while 48.6% and 37.1%, respectively, fell in the SE quadrant. Thus, conclusions about cost-effectiveness are contingent on the assumed scenario.

We estimated a per-individual 0.008 gain in QALYs, which translates to a gain of 800 years of full health across a population of 100 000 individuals, with healthcare costs for the DAI strategy estimated at €297.6 million compared with €306.5 million for the TAU strategy. The gain in QALYs was due to reduced incidence of disease, for example, we found that the digital intervention could save a total of 57 cases of colorectum cancer in a population of 100 000 individuals (who would normally experience 2905 cases). This disease-specific impact not only highlights the clinical relevance of the intervention but also serves as an additional metric for transparency and validation of our model. While these findings indicate meaningful health gains and cost savings at the population level, their translation into real-world impact depends on successful implementation beyond the modelled setting. Clear institutional ownership is essential to ensure population-level reach, sustained engagement and the realisation of projected health and economic benefits.

The effectiveness of the DAI strategy translates into relatively modest decreases in alcohol consumption per person, approximately 24 g of alcohol per week.^12^ Although any reduction in alcohol consumption is beneficial at the individual level, it is the population level impact that this modest effect has which is displayed in this study. From our findings it is also clear that the effect of the digital intervention was insufficient to have a major impact on the incidence and burden of alcohol-related diseases with a weaker causal pathway across the population. Achieving broader disease benefits may require interventions with larger effect sizes that can further reduce alcohol consumption. This was made even more clear in the other scenarios presented in online supplemental appendix B, where the effects of the intervention on diseases with weaker causal pathways were strongly attenuated towards the null, also increasing the uncertainty about the cost-effectiveness of the intervention.

The results of this study are contingent on the assumption that the intervention incurs no costs, which would not be true if it were to be disseminated on a larger scale in society. A large-scale dissemination would require large scale advertisement, which would incur significant costs; thus, the intervention would necessarily be considered less cost-effective. On the other hand, today’s TAU consists of health information on webpages which are already advertised, meaning that costs exist on both sides of the contrast. Given the expected gain in QALY in the DAI cohort, and considering a commonly used threshold of cost-effectiveness of €50 000 per QALY, the cost of the intervention per se could be €489 per individual (and added to the healthcare costs of €2976 estimated for the DAI strategy) and still yield an ICER below €50 000, assuming that TAU is free. It is highly unlikely that the cost per individual of the intervention would be higher than this estimated upper bound in a large scale roll out.

Studies examining the long-term effects of brief DAIs have generally not extrapolated their health impacts over extended periods, nor have they modelled the impact on incidence rates. Digital interventions nevertheless demonstrate in the short-term to be cost-effective compared with alternative approaches.^10^ Additionally, a systematic review highlighted that brief non-DAIs, particularly in primary care settings, represent a cost-effective strategy for addressing harmful alcohol consumption. When modelling techniques were applied, nearly all studies indicated that these interventions were either cost-saving and health-enhancing (ie, dominating a ‘do-nothing’ scenario) or incurred very low costs relative to the health gains achieved.^15^ However, it should be noted that the magnitude of health gains and potential cost savings depends heavily on the assumptions regarding the long-term effect of the intervention, as indicated by our scenario analyses. Thus, while previous studies may have suggested cost-effectiveness, the limitations raised in the current study also apply to those that came before.

### Limitations

The validity of assumptions and data in health economic models is crucial. Uncertainties and biases can significantly impact results, so they must be considered when interpreting findings. A strength of our simulation model is that uncertainties regarding input data are propagated into the results, as we allow parameters to be drawn from provided distributions. This means that each simulation run includes variations based on distributions for each parameter, reflecting the uncertainty in the data and providing a more realistic representation of outcomes. However, this does not mitigate the potential risk of bias in estimates used as input. The parameters used in our model are based on input data obtained from existing literature, and our own assumptions where data could not be found. For example, we assumed that individuals who developed cancer would not be subjected to a higher risk of mortality after 5 years, which should be considered when interpreting the results.

It is important to acknowledge that data from existing literature may be subject to bias, depending on the quality of the data and assumptions made in the original analyses. This is particularly relevant regarding the risk estimates of alcohol on the cardiovascular system and cancer that we have included in the model.^16^ Since it is not possible to conduct randomised controlled trials to validly estimate the effects of alcohol on health, we instead rely on observational studies for these estimates. In observational studies, controlling for confounding factors is inherently challenging, which may lead to bias in risk estimates concerning the effects of alcohol on health. Observational studies also may rely on individuals recalling their past alcohol consumption, which may introduce recall^17^ and social desirability bias.^18^ In addition, alcohol research has purposely been manipulated by actors with competing interest, including the alcohol industry.^19^

Another limitation concerns our use of standard mortality data to calculate population mortality risk. Standard mortality data already includes causes of death from a range of factors, including alcohol-related disease. This means that mortality is slightly higher in our simulations than we should expect in the real-world. It is important to address this factor moving forward, which would require more granular mortality data than is currently publicly available. A related limitation is that we use relative risks associated with alcohol consumption to calculate the risk of incidence of the modelled diseases. However, the incidence data that we have access to includes cases who consume alcohol, thus, the baseline incidence rate that we use is not an alcohol-free incidence rate. This means that we should expect to see a slightly higher incidence rate of diseases where the incidence rate is strongly affected by alcohol consumption. This potential double-counting of mortality and alcohol risk can lead to an overestimation of mortality and morbidity; however, this limitation is somewhat mitigated due to it being present in both sides of the contrast, that is, when comparing simulated populations with and without public health interventions, both are affected by these biases, and they cancel out to a degree in the findings.

We explored the effects of altering the population level effect in four different scenarios and showed how it affects the uncertainty of the cost-effectiveness plane. Another consequence of this modelling assumption is that the excess risks of alcohol consumption accumulating over time is not explicitly modelled; thus, an individual who has consumed a certain amount for a long time should have a higher risk than somebody consuming the same amount but for a shorter period. However, it should be noted that weekly alcohol consumption as modelled here, and how it is typically operationalised in the literature—including the observational studies from which we extracted risk estimates—represents a pattern of behaviour rather than a specific level of consumption. It is therefore more accurate to think of the measure of weekly consumption used here as something representing a behaviour of the simulated individual rather than their actual consumption. Moving forward, the simulator would benefit by explicitly including consumption trajectories and incorporate a more dynamic use of the intervention over time. For example, individuals in the DAI cohort could have re-engaged with the support after a few years if their alcohol consumption had increased and they wanted support again.

Finally, our simulations did not consider all diseases that have been shown to be linked with alcohol. The Sheffield Alcohol Policy Model, which aimed to evaluate public health strategies for alcohol harm reduction, included 47 acute and chronic conditions related wholly or partially to alcohol. Clearly, there are opportunities to extend our simulations to include a broader range of conditions, including mental health disorders. A direct consequence of this is that we expect our findings in this study to underestimate negative health outcomes and costs. What is more, the focus of this study was on the negative consequences to the health of the drinker; however, alcohol consumption causes severe harms to others who are in proximity to those who drink and overall places a large burden on society and welfare systems.^20^ Analysing costs and effects from a societal perspective would, therefore, have provided a broader understanding of the consequences of disseminating the DAI. However, there is no general agreement on which consequences should be included and how they should be measured and valued.^9^ Consequently, there is more research needed to fully understand the long-term outcomes associated with the dissemination of DAIs and the role they play as part of prevention strategies. Indeed, a key contribution of this work is that our simulator provides a framework for such extended analyses.

## Conclusions

The findings from this study demonstrate that a DAI could reduce the incidence of alcohol-related diseases, increase QALYs and lower healthcare costs, under the assumption that the effects remain stable over time. However, considerable uncertainty remains regarding its cost-effectiveness, and increasingly so when varying assumptions about effectiveness were explored. There are challenges that need to be addressed to increase the strength of evidence for the cost-effectiveness of DAIs, including reducing the uncertainty and bias in parameters necessary for modelling. In addition, the realisation of the benefit in practice depends on effective implementation, including sustained investment in dissemination, clear institutional ownership and governance structures that support long-term engagement. There is also further need for research to incorporate long-term societal impacts from alcohol, not only considering healthcare costs.

## Supplementary material

10.1136/bmjph-2025-003503 online supplemental file 1

10.1136/bmjph-2025-003503 online supplemental file 2

10.1136/bmjph-2025-003503 online supplemental file 3

## Data Availability

Data are available on reasonable request.
