# Supplementary material for: Long-term outcomes of a digital alcohol intervention targeting online help-seekers: a simulation study of incidence of disease, quality-adjusted life-years and costs
Source: BMJ Public Health. 2026 Jun 24;4(2):e003503. doi: 10.1136/bmjph-2025-003503 (PMC13295776; doi:10.1136/bmjph-2025-003503)
Supplement: online supplemental file 1 [file bmjph-4-2-s001.docx]

Long-term outcomes of a digital alcohol intervention targeting online help seekers - a simulation study of incidence of disease, quality-adjusted life years, and costs

Supplementary materials

# Appendix a - Model structure and analytical assumptions

This appendix contains a detail description of the individual-level simulator and its parameters. The simulator is designed to simulate the life course of individuals in a population. The population could, for instance, be a specific patient group, a birth cohort, or the general population of a country. The individuals who make up the simulated population are defined by their characteristics, which consists of their biological sex, age, health behaviours, prevalent diseases, history of diseases, and interventions. When contrasting interventions, the population is duplicated and one of the copies is given access to the intervention while the other is not. By doing so, simulated outcomes in the two otherwise identical populations can be contrasted, and decision support can be provided for the dissemination of the intervention in terms of difference in incidence of disease, health-related quality-of-life, and costs.

## 1 - Costs and health-related quality-of-life

Health-related quality of life is measured using quality-adjusted life-years (QALY). Costs accumulate for individuals depending on their disease state and interventions that they have access to. Similarly, QALYs accumulate for the individuals, weighted by QoL-weights associated with their diseases. Depending on the disease, costs and QoL-weights may be applied for life or until the disease is considered cured. All costs are reported in euro, however, input data for costs typically comes from the research literature and may therefore be reported in different currencies and for different years (i.e. price indices). The simulator updates these reported costs, taking into account inflation in the reported currency and then converting them into euro according to current exchange rates.

## 2 - Model structure

A simulation progresses by updating the state of the individuals for a number of *cycles*. A cycle is a unit of time that defines the granularity for which the simulations are run. Typically, which is also the case here, a cycle represent one year. A simulation can last for any number of cycles. In Supplementary Figure 1, we present a schematic overview of how the state of the simulated individuals are updated in a single cycle. Each step is described below.

1. In each cycle, each individual who make up the cohort is updated in turn.
2. Nothing is updated if the individual is deceased.
3. The behaviour of the individual is updated. For instance, their alcohol consumption may change depending on their personal characteristics including age, gender, diseases, treatments, etc. These updated behaviours are used throughout the current cycle to calculate individual level risk ratios.
4. Diseases that are already prevalent in the individual are progressed. For instance, if the individual has alcohol-related liver cirrhosis, it may progress to liver cancer. Progression may be conditional on the individual’s characteristics.
5. The individual is diagnosed with diseases conditional on their individually calculated hazard. These hazards are based on age and sex specific incidence rates attenuated by risk ratios associated with the individual’s behaviours.
6. The individual’s total cost and QALY are updated based on the diseases and treatments that they have. The non-disease specific baseline QALY of an individual is defined by their age, which is weighted conditional on the diseases they have. Costs and QALY can be discounted at specified rates.
7. The alive status of the individual is updated based on their prevalent diseases and baseline mortality. For each disease that is prevalent in the individual there is a disease specific mortality probability which is conditional on their age and sex. If surviving all prevalent diseases, then the risk of dying due to all other circumstances (the baseline mortality) is taken into account.
8. If the individual survives the cycle, then age is increased by one year (or cycle unit) before the cycle ends for the individual.


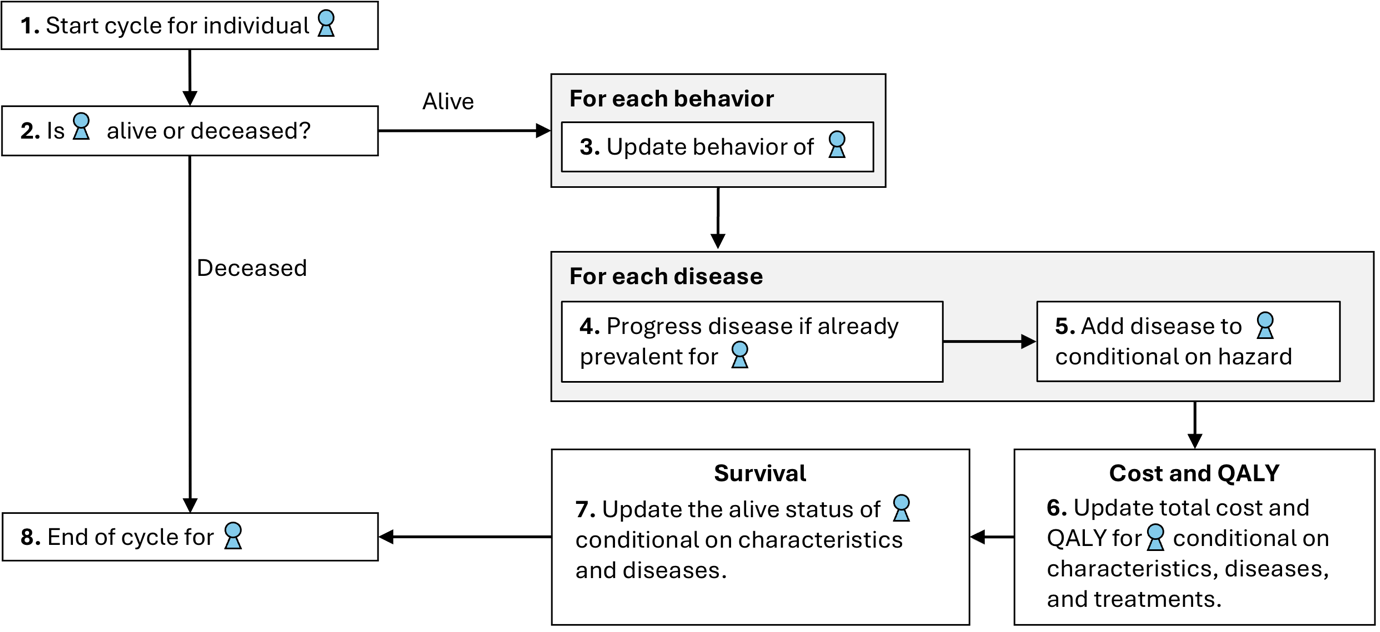


Supplementary Figure 1 - A schematic overview of how the state of a simulated individual is updated during a cycle of the simulator.

## 3 - Data inpuT

The simulator is guided by parameters derived from research literature, public reports, and registry data. A coarse division of these parameters can be made into three categories: incidence, mortality, and cost and QoL. Incidence parameters include incidence rates of disease in the simulated population, risk of progression of disease, and disease incidence risk ratios associated with health behaviours, in our case alcohol consumption. Mortality parameters include baseline mortality rates in the population and mortality rates associated with diseases. Cost and QALY parameters include costs and weights that are associated with a disease and interventions.

### 3.1 - Incidence parameters

#### 3.1.1 - Incidence rates, stages, and progression of disease

We derived annual incidence rates of disease from data published by the Swedish National Board of Health and Welfare. We used data from 2017 to 2021 for which incidence rates were available separately for men and women subdivided into age groups (0-4, 5-9, 10-14 years of age, etc.). We averaged the incidence rates across 2017 to 2021 and used the same incidence rate for each age within the age groups, i.e., 0-, 1-, 2-, 3-, and 4-year-olds all had the same incidence rate which was the average rate across years divided by five. There were no data published by the Swedish National Board of Health for alcohol-related liver disease, instead, we used data from a published report with incidence rates from 2021 [1].

Parameters for the stage of detection of cancer diseases were taken from the literature. A summary of the distribution of stages is shown in Supplementary Table 1. For some cancer diseases, e.g. colorectum cancer, the studies from which we extracted the stage probabilities included cases where the stage at detection was unknown. For these diseases, we normalised the probabilities for stage detection so that all simulated cancer diseases were assigned a stage.

For breast cancer, we used an annual risk of contralateral cancer of 1.5% and an annual risk of 18% of recurrence [2]. Risk of progressions of breast cancer was only applied for five years, after which the disease was considered cured (as was the case for all cancer diagnoses). For alcohol-related liver disease, we used an annual risk of 1.9% for progression to liver cancer [3].

Parameters for increased risk of stroke recurrence for individuals aged 65 or older were also taken from the literature [4]. The hazard ratios for stroke recurrence, applied to both haemorrhagic and ischemic stroke, are presented in Supplementary Table 2.

Supplementary Table 1 - Distribution of cancer stages when detected.

|  | **Stage** | | | | |  |
| --- | --- | --- | --- | --- | --- | --- |
|  | **I** | **II** | **III** | **IV** | **Unknown** | **Context (year) reference** |
| **Breast cancer (Normalised)** | 56% (56%) | 37% (37%) | 5% (5%) | 2% (2%) | - | Sweden (2010) [5] |
| **Colorectum cancer (Normalised)** | 12% (14%) | 30% (34%) | 26% (29%) | 20% (23%) | 12% (-) | Sweden (2013) [6] |
| **Oesophageal** **cancer (Normalised)** | 4% (6%) | 12% (19%) | 19% (30%) | 29% (45%) | 36% (-) | Netherlands (2005) [7] |
| **Liver cancer (Normalised)** | 33% (33%) | 21% (21%) | 38% (38%) | 8% (8%) | - | Sweden (2024) [8] |
| **Oral cancer (Normalised)** | 37% (37%) | 36% (36%) | 18% (18%) | 9% (9%) | - | South Korea (2013) [9] |
| **Pancreas cancer (Normalised)** | 7% (9%) | 17% (22%) | 10% (13%) | 43% (56%) | 23% (-) | Denmark (2020) [10] |

Supplementary Table 2 - Hazard ratios for haemorrhagic and ischemic stroke recurrence.

| **Stroke recurrence** | | |
| --- | --- | --- |
| **Age** | **Hazard ratio (95% confidence interval)** | **Reference** |
| **>= 85 years old** | 2.52 (1.62; 3.93) | United Kingdom (2020) [4] |
| **>= 75 years old** | 1.85 (1.32; 2.60) | United Kingdom (2020) [4] |
| **> 65 years old** | 1.47 (1.06; 2.04) | United Kingdom (2020) [4] |
| **<= 65 years old** | Reference | United Kingdom (2020) [4] |

#### 3.1.2 - Weekly alcohol consumption incidence hazard ratios

We derived the relative risk of incidence of disease associated with weekly alcohol consumption from estimates found in the research literature. Point estimates and confidence intervals of hazard ratios were sometimes reported per 100 grams of alcohol consumed per week, and sometimes as intervals of weekly consumption, e.g. 0 to 10 grams, 11 to 35 grams, etc. When standard drinks were used in the reporting, we converted these to grams using definitions used in the country where the study was conducted unless otherwise specified.

When hazard ratio point estimates and confidence intervals were reported per 100 grams of weekly alcohol consumption, we converted these to log hazard ratios per 1 gram. We used the confidence interval to calculate a standard deviation around the point estimate [11]. Log hazard ratios were sampled from a normal distribution using these derived parameters. Throughout the simulations, when the incidence rate of a disease was being calculated for a specific individual, we sampled a log hazard ratio from this normal distribution, multiplied the log hazard ratio with the individual’s current alcohol consumption, and added to the individual’s log incidence rate for the specific disease.

When hazard ratio point estimates and confidence intervals were presented in terms of intervals of weekly consumption, we used these parameters to generate synthetic data to estimate the log hazard ratio per 1 gram. This was done by first sampling log hazard ratios from a normal distribution for each consumption interval using the log transformed point estimate as mean and a standard deviation calculated based on the reported confidence interval. In total, we sampled 10,000 log hazard ratios, with the number of samples per consumption interval proportional to the interval length. We then paired each sampled log hazard ratio with a weekly alcohol consumption sampled uniformly within each respective consumption interval. We used these synthetic data to estimate a natural cubic spline regression model with knots at the reported consumption interval bounds. The regression models were estimated using Bayesian inference. For the intercept, we used a Cauchy prior centred at 0 with scale of 1. For covariates, we used Cauchy priors centred at 0 with a standard normal hyperprior for the scale. Finally, for the standard deviation parameter we used a half-Student’s t prior centred at 0 with 3 degrees of freedom and a scale of 2.5. The Cauchy shrinkage priors were chosen as the splines could not adequately fit the data when using Student’s t priors for some diseases for which the risks grew strongly with consumption (e.g., alcohol-related liver disease).

We used the mean and standard deviation of the marginal posterior distribution of each spline regression model parameter to define a distribution over models. We began each simulation by sampling, for each disease, a coefficient for the intercept, a coefficient for each covariate (i.e., weights for the splines), and a standard deviation. Thus, at the start of each simulation we sampled a spline regression model for each disease and used this model throughout the simulation. When the incidence rate of a disease was being calculated for a specific individual, log hazard ratios were sampled from a normal distribution defined by the sampled spline regression model conditional on the simulated individuals’ characteristics.

##### 3.1.2.1 - Cancers

For cancer disease, we derived hazard ratios associated with weekly alcohol consumption from estimates from a study conducted in Australia [12]. The study analysed the relationship between alcohol consumption and cancer risk using data from a cohort including 226,162 participants aged 45 years and older. Alcohol consumption was categorised into weekly alcohol consumption ranges, with the 10–35-gram range used as reference. Supplementary Table 3 contains the hazard ratio estimated that we used to derive parameters for our simulator using natural cubic spline regression.

In Supplementary Figure 2 Panel A, we have, for breast cancer, plotted the point estimates and confidence intervals for hazard ratios associated with the weekly alcohol consumption intervals presented in Supplementary Table 3, i.e., the data extracted from the literature (Raw input data). The data that we generated to estimate the natural cubic spline regression models are also plotted (Generated). Finally, posterior predictions of hazard ratios from the regression models are plotted (Predicted). In panels B to F of Supplementary Figure 2, plots are presented for the other modelled cancer diseases.

Supplementary Table 3 - Cancer disease hazard ratios associated with weekly consumption of alcohol.

| **Hazard ratio per different category of amount of alcohol consumed** | | | | | | |
| --- | --- | --- | --- | --- | --- | --- |
| **Grams of alcohol** | **0 -<10** | **≥10 - ≤35** | **>35 - ≤70** | **>70 - ≤140** | **>140 - ≤280** | **>280** |
| **Breast cancer** | | | | | | |
| Hazard ratio | 0.91  (0.80-1.03) | Ref | 1.02  (0.89-1.17) | 1.15  (1.00-1.34) | 1.17  (0.97-1.42) | - |
| **Colorectum cancer** | | | | | | |
| Point estimate  (95% CI) | 1.03  (0.9-1.18) | Ref | 0.97  (0.83-1.14) | 1.14  (0.98-1.33) | 1.23  (1.03-1.46) | 1.3  (1.03-1.62) |
| **Oesophageal cancer** | | | | | | |
| Point estimate  (95% CI) | 1.55  (0.90-2.67) | Ref | 1.07 (0.58-1.98) | 0.9  (0.47-1.70) | 1.21  (0.64-2.30) | 2.11  (1.07-4.13) |
| **Liver cancer** | | | | | | |
| Point estimate  (95% CI) | 1.93  (1.08-3.47) | Ref | 1.09  (0.54-2.17) | 1.48  (0.76-2.86) | 1.19  (0.57-2.50) | 3.02  (1.49-6.13) |
| **Oral cancer** | | | | | | |
| Point estimate  (95% CI) | 0.77  (0.57-1.04) | Ref | 0.73  (0.52-1.02) | 1.07  (0.78-1.46) | 1.19  (0.85-1.66) | 1.80  (1.24-2.62) |
| **Pancreas cancer** | | | | | | |
| Point estimate  (95% CI) | 1.09  (0.79-1.49) | Ref | 1.09  (0.77-1.55) | 1.31  (0.92-1.87) | 1.23  (0.82-1.84) | 0.92  (0.50-1.68) |

| Panel A – Breast cancer |
| --- |
| 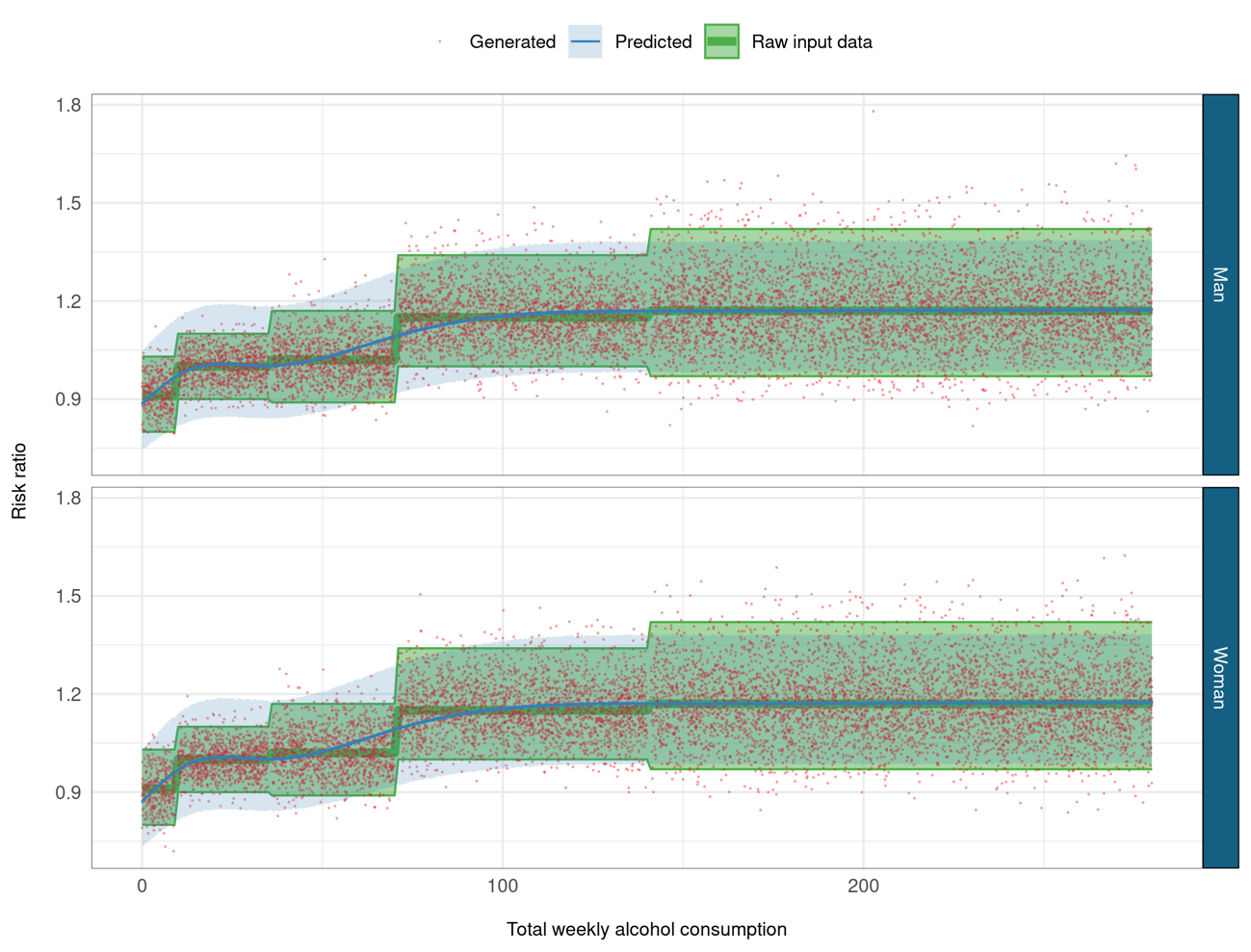 |
|  |
| Panel B – Colorectum cancer |
| 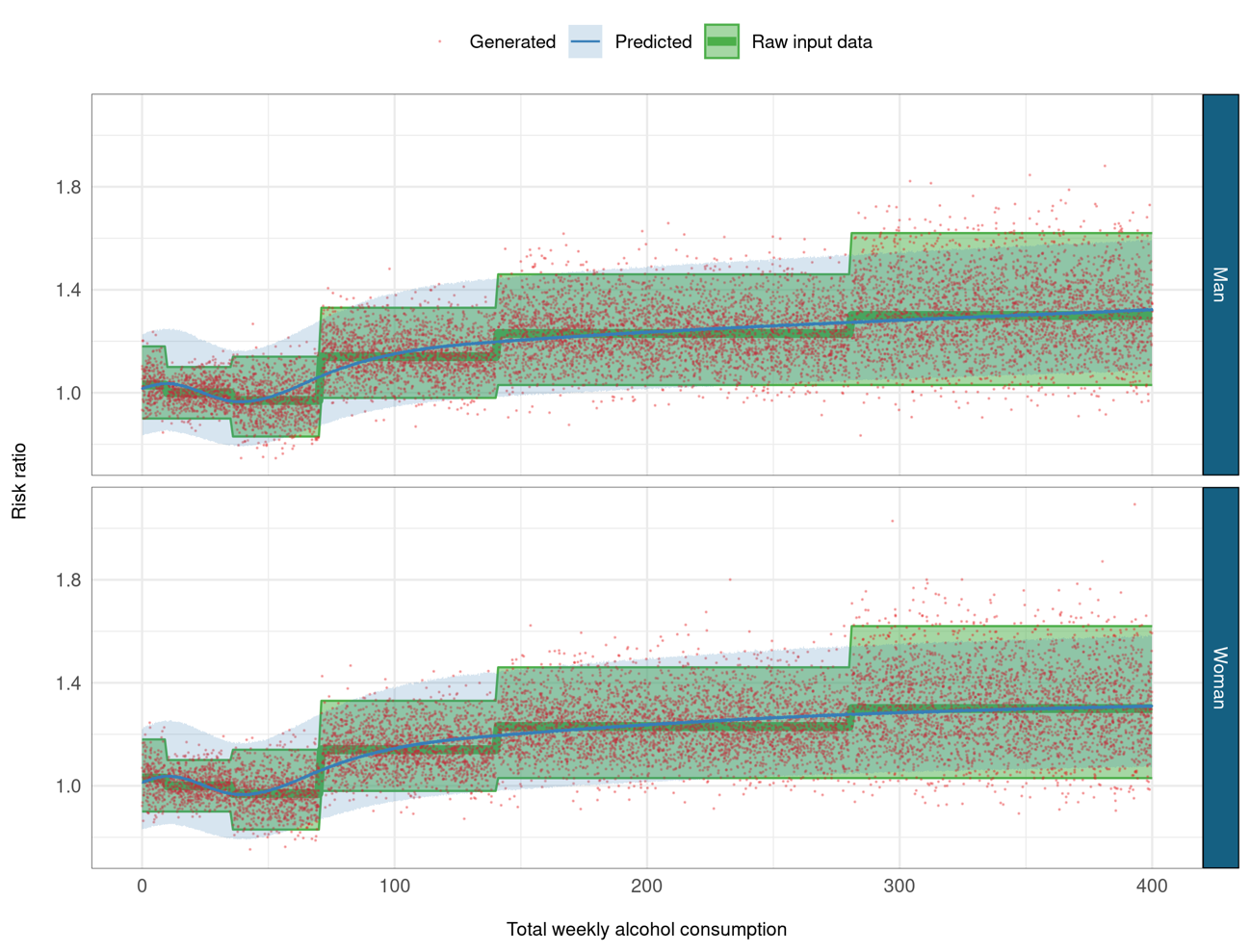 |
|  |
| Panel C – Oesophageal cancer |
| 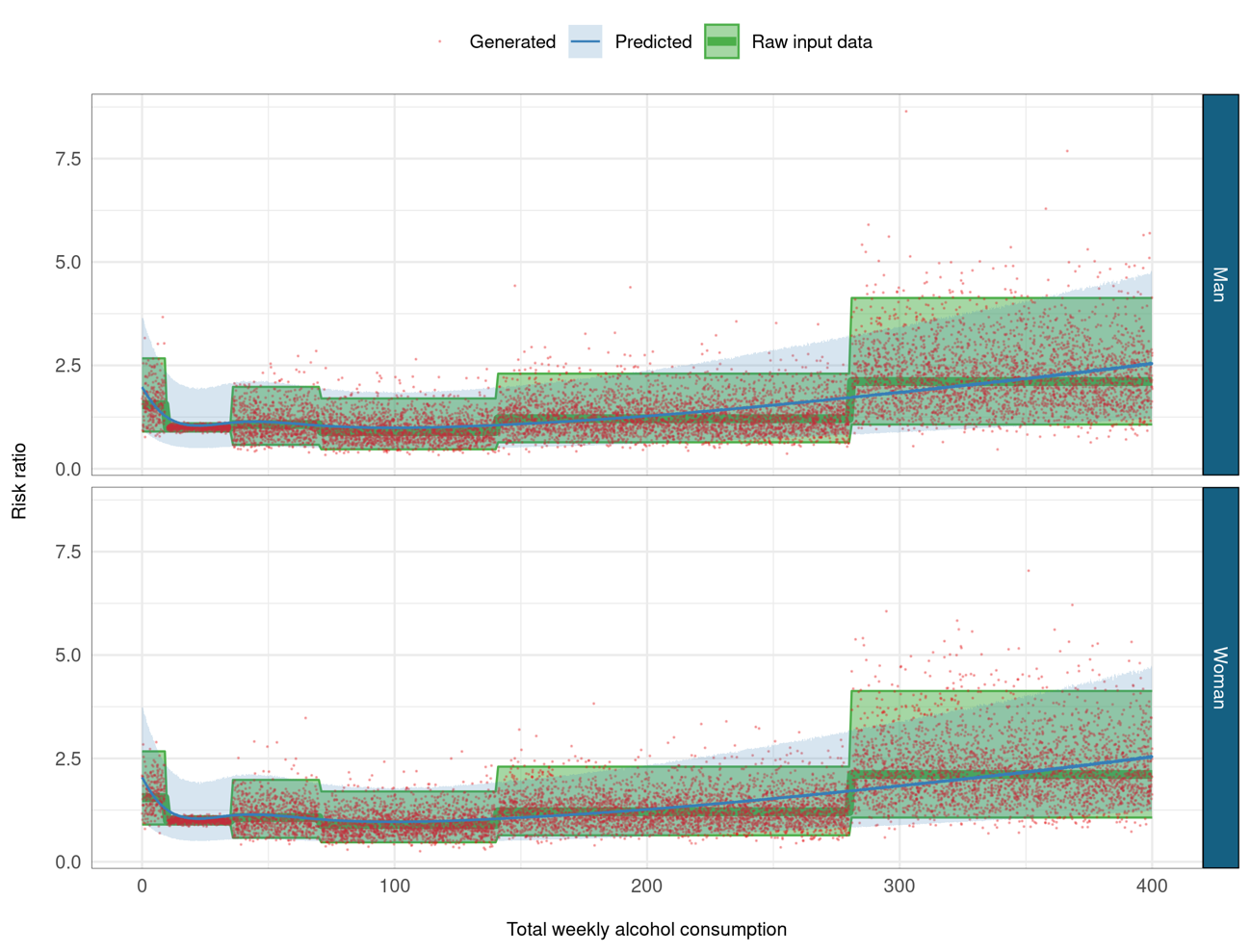 |
|  |
| Panel D – Liver cancer |
| 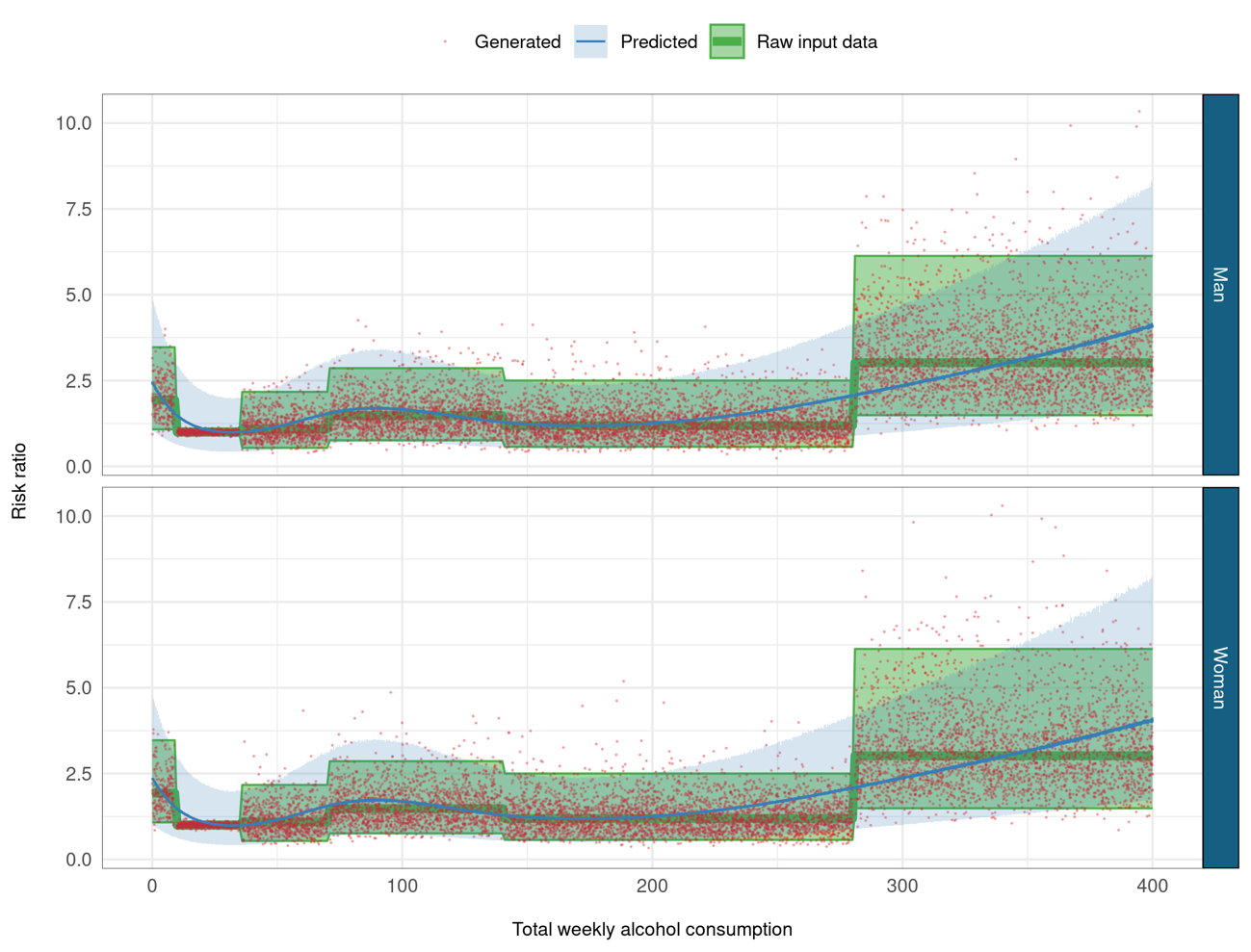 |
|  |
| Panel E – Oral cancer |
| 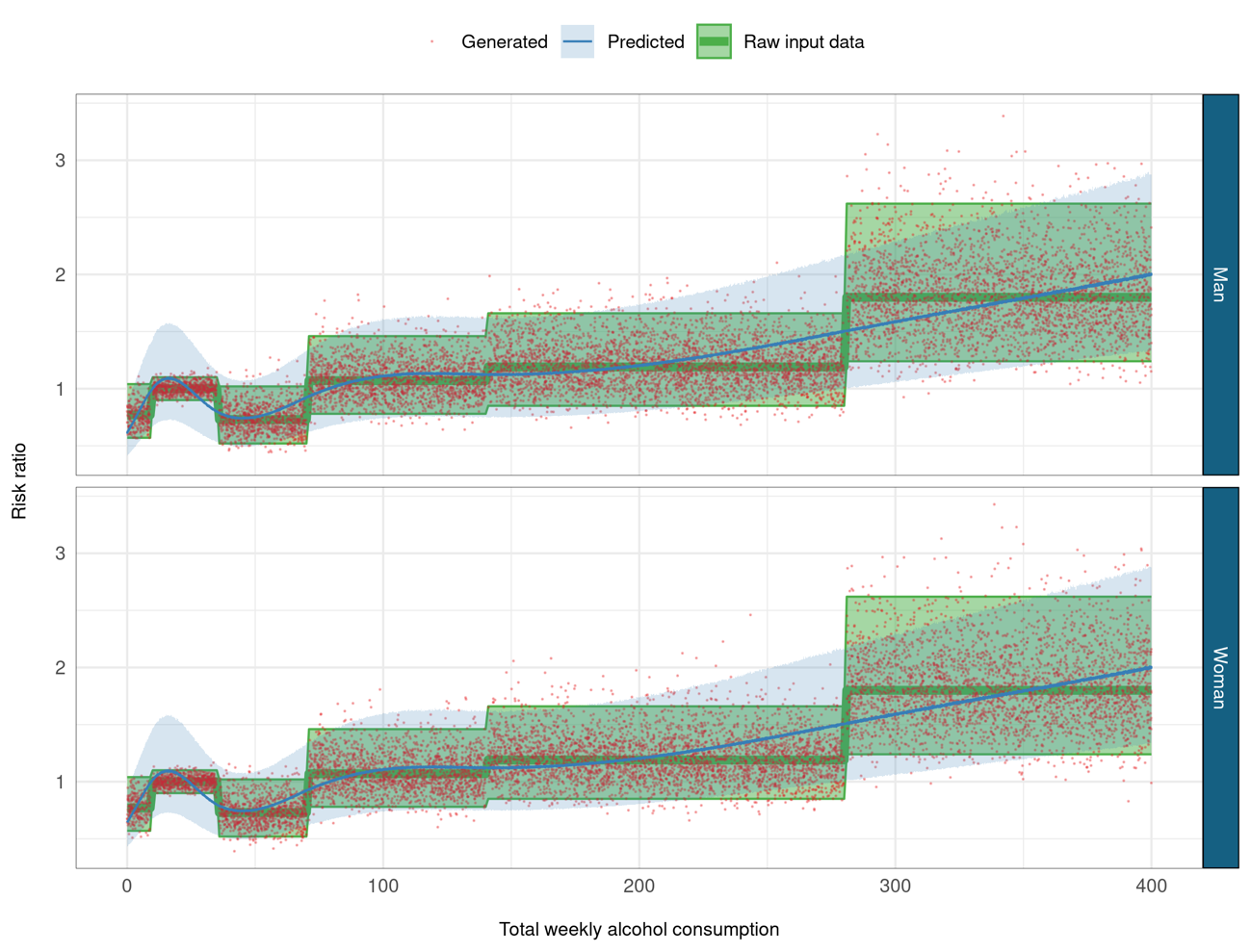 |
|  |
| Panel F – Pancreas cancer |
| 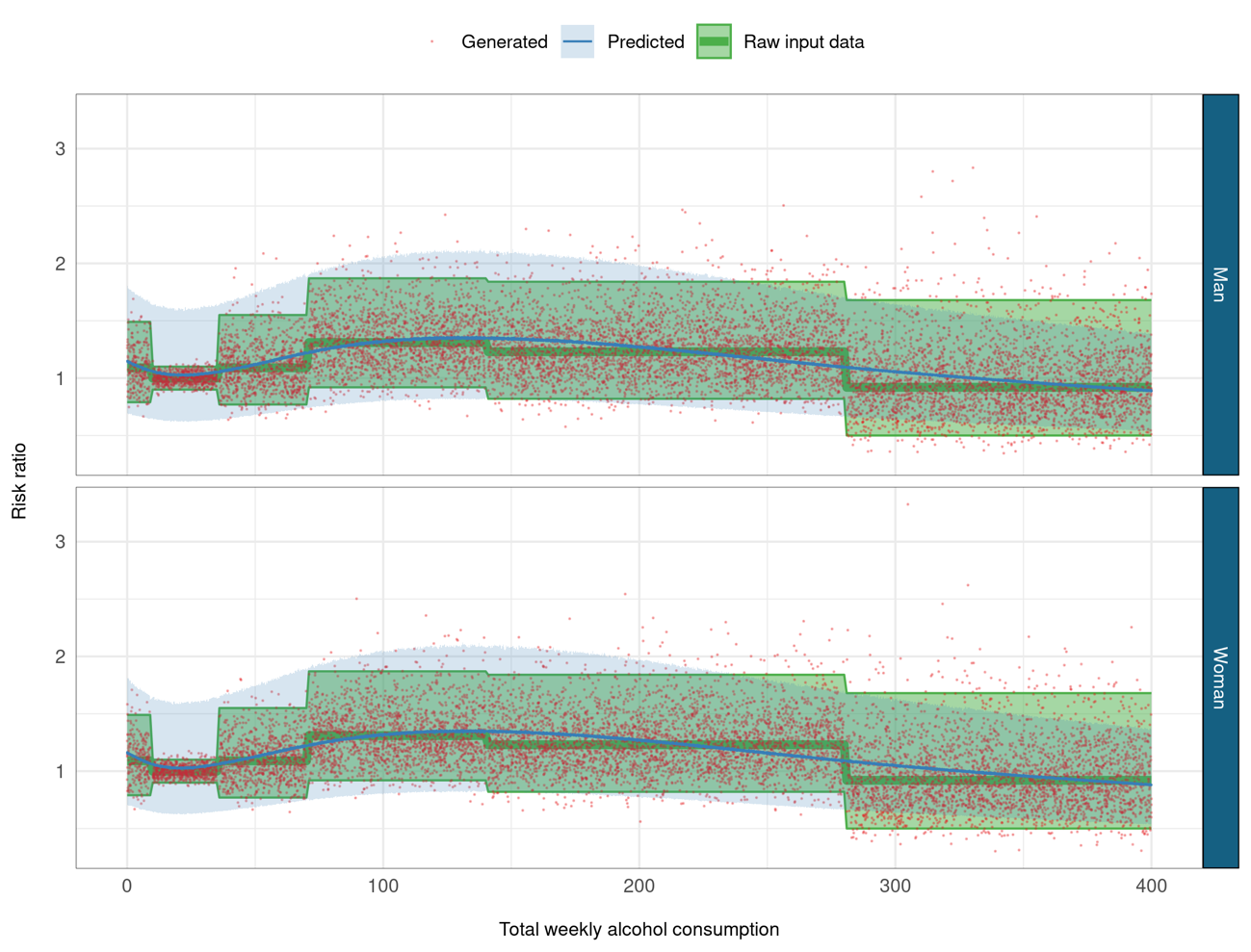 |
|  |
| Panel G – Alcohol-related liver disease |
| 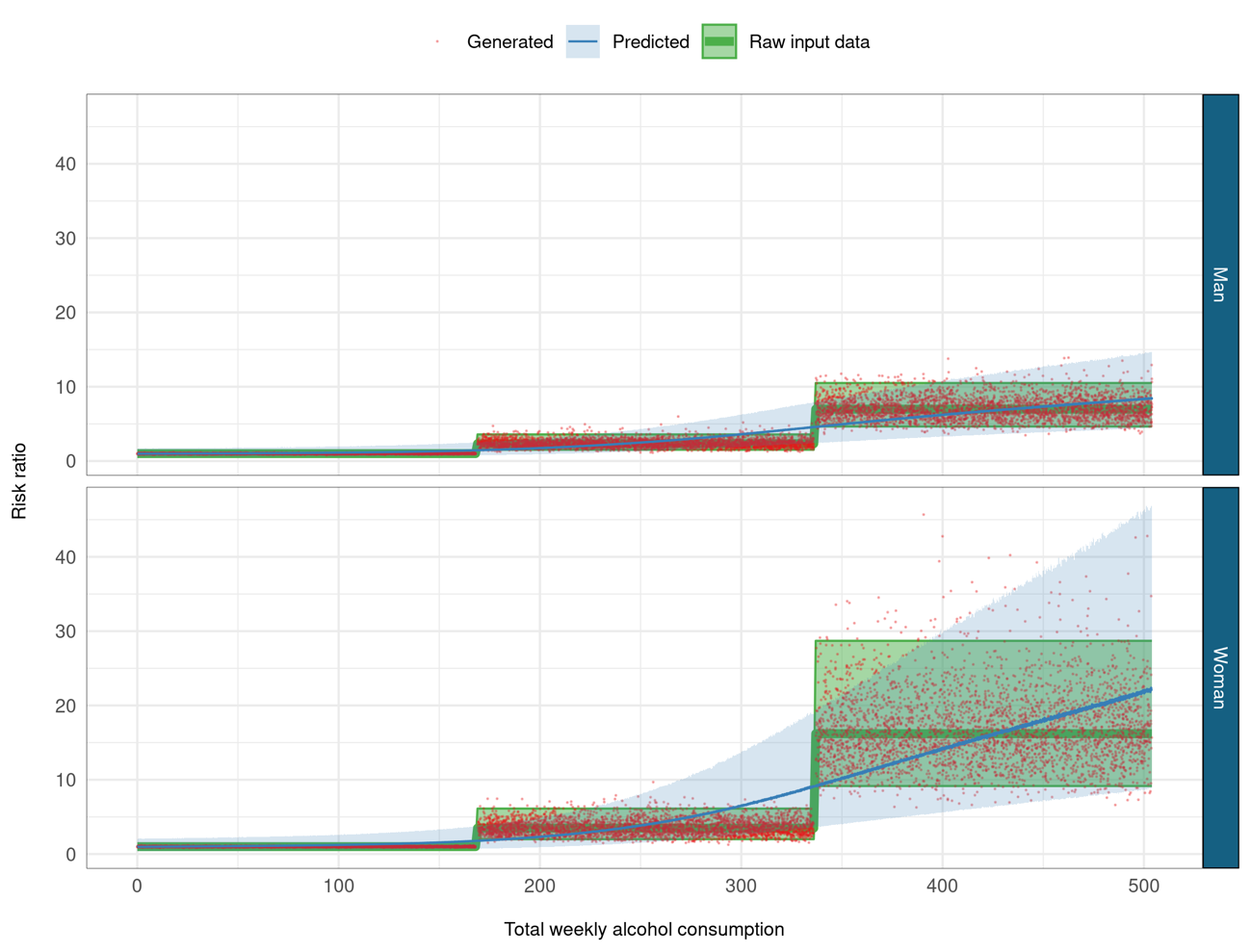 |

Supplementary Figure 2 – Point estimates and confidence intervals for hazard ratios associated with weekly alcohol consumption intervals extracted from the literature (Raw input data). Data generated to estimate regression models (Generated). Posterior predictions of hazard ratios from natural cubic spline regression models are plotted (Predicted).

##### 3.1.2.2 - Alcohol-related liver disease

For alcohol-related liver disease, we derived hazard ratios associated with alcohol consumption from estimates from a study of a Danish cohort [13]. The study analysed 55,917 participants 50-64 year of age. Supplementary Table 4 contains the reported hazard ratio estimates, stratified by sex, with 0-168 grams used as reference. In Supplementary Figure 2 Panel G, we have, as we did for cancer diseases, plotted the input data, the generated data, and the predicted hazard ratios for weekly alcohol consumption and alcohol-related liver disease that we generated using the cubic spline regression model and used in the simulator.

Supplementary Table 4 - Alcohol-related liver disease hazard ratios associated with weekly consumption of alcohol.

| **Hazard ratio per different category of amount of alcohol consumed** | | | |
| --- | --- | --- | --- |
| **Grams of alcohol** | **0-168 gram** | **169-336 gram** | **337-504 gram** |
| **Alcohol-related liver disease – Men** | | | |
| Point estimate (95% CI) | Ref | 2.33 (1.52-3.58) | 6.98 (4.65-10.5) |
| **Alcohol-related liver disease – Women** | | | |
| Point estimate (95% CI) | Ref | 3.49 (2.00-6.12) | 16.2 (9.16-28.7) |

##### 3.1.2.3 stroke and myocardial infarcation

For haemorrhagic stroke, ischemic stroke, and myocardial infarction, we retrieved hazard ratio point estimates associated with weekly alcohol consumption from the Global Burden of Disease study [11]. The study combined individual-participant data for 599,912 current drinkers in 83 prospective studies. In Supplementary Table 5, the reported hazard ratios per 100 grams of alcohol per week are presented for these three diseases.

Supplementary Table 5 - Stroke and myocardial infarction hazard ratios associated with weekly consumption of alcohol.

| **Hazard ratios per 100 gram of alcohol per week** | **Point estimate** | **95% CI** |
| --- | --- | --- |
| Myocardial infarction | 0.93 | 0.90; 0.97 |
| Haemorrhagic stroke | 1.17 | 1.12; 1.23 |
| Ischemic stroke | 1.13 | 1.09; 1.18 |

### 3.2 - Mortality parameters

#### 3.2.1 - Non-disease specific mortality

We derived parameters for non-disease specific mortality from life tables published by Statistics Sweden. The probability of dying for a person of a specific age (0 to 100 years) and biological sex was calculated as the average across the years 2019 through 2023.

#### 3.2.2 - Disease specific mortality

We derived disease specific mortality probabilities from the Swedish National Board of Health and Welfare’s Cause of Death register. Data were extracted for the years 2017 to 2021. The data presents the annual number of cases and number of deaths of a specific disease stratified by sex and age groups (0-4, 5-9, 10-14 years of age, etc.). Annual mortality probabilities calculated as the proportion of deaths among cases may vary substantially year over year, and mortality data can in some years be missing entirely for groups in which a disease is rare. Therefore, we used the data to estimate mortality probability using natural cubic spline logistic regression, allowing us to aggregate across years and define disease specific mortality probabilities for all individuals, conditional on their sex and age.

The procedure to estimate disease specific mortality was as follows. We first collapsed the input data across years by summing the number of cases and deaths for each sex and age group. Deaths divided by cases represented the mortality rate for each group. We used the same mortality rate for each age within the ranges, i.e., 0-, 1-, 2-, 3-, and 4-year-olds all had the same incidence rate which was the group rate divided by five. We then generated a synthetic dataset with cases which matched the mortality rate for each sex and age group, for instance, we generated 500 women of age 50 of which a number were deceased proportionate with the mortality rate for the group. We estimated a natural cubic spline logistic regression model of the mortality probability using the generated synthetic dataset. Estimation was done using Bayesian inference with Student’s t priors for all parameters, centred at 0 with 3 degrees of freedom and a scale of 2.5 for all parameters.

During simulations, we used the mean and standard deviation of the marginal posterior distribution of each regression model parameter to define a distribution over models. We began each simulation by sampling, for each disease, a coefficient for the intercept and each covariate (i.e., weights for the splines). During the simulation, when the mortality probability of a disease for a specific individual was calculated, we used the sampled model to calculate a mortality probability conditional on individual’s characteristics.

### 3.3 - Cost and quality-adjusted life-years

Non-disease specific baseline QoL-weights were retrieved from a Swedish study [14]. These weights are presented in Supplementary Table 6. The study did not provide QoL-weights for ages under 30 years. We have, therefore, assumed the same weights for ages 0 to 29 years as for the age group 30-34 years. These non-disease specific weights were used as a baseline QoL for the simulated individuals and were adjusted conditional on the individuals’ diseases.

In this study, we have assumed that the disease specific QoL-weights were independent of each other. The adjustment for the disease specific QoL-weights was done by multiplying the baseline weight for an individual with the QoL-weights associated with the individual’s diseases. For instance, for an individual with a baseline QoL of 0.9 who had two diseases with QoL-weights of 0.8 and 0.7, the resulting QoL-weight was: 0.9 x 0.8 x 0.7 = 0.504. This QoL-weight was then further adjusted by the annual discount rate.

Supplementary Table 6 - Non-disease specific baseline QoL-weights.

| **Age group** | **QoL-weight** |
| --- | --- |
| 0-29 years | 0.911 |
| 30-34 years | 0.911 |
| 35-39 years | 0.922 |
| 40-44 years | 0.916 |
| 45-49 years | 0.913 |
| 50-54 years | 0.903 |
| 55-59 years | 0.901 |
| 60-64 years | 0.904 |
| 65-69 years | 0.912 |
| 70-74 years | 0.904 |
| 75-79 years | 0.887 |
| 80-84 years | 0.858 |
| 85-89 years | 0.824 |
| 90-94 years | 0.783 |
| 95-104 years | 0.718 |

#### 3.3.1 Breast cancer

The QoL-weights for breast cancer were retrieved from a Swedish study and estimated using EQ-5D [15]. The weights in Supplementary Table 7 apply the first and following years of breast cancer and recurrence of cancer. The same weight was used for individuals developing contralateral cancer. We assumed a reduction in QoL due to breast cancer only in the first 5 years.

The cost associated with breast cancer is based on a Swedish study and presented in Supplementary Table 7 [2]. The costs are assumed to be the same for developing breast cancer for the first time and contralateral cancer. The costs associated with recurrence of cancer differ from the costs of an initial cancer diagnosis in the first year but are the same following years. In the referenced study, costs were originally reported in Euro adjusted to the 2007 price index.

Supplementary Table 7 - Cost and QoL-weights for breast cancer.

| **Breast cancer** | | **Reference** |
| --- | --- | --- |
| **QoL-weights** | **Estimate (95% CI)** |  |
| **First year after breast cancer / contralateral cancer** | 0.696 (0.634;0.747) | [15] |
| **First year after recurrence** | 0.779 (0.700;0.849) | [15] |
| **Following 4 years (first, contralateral, and recurrence)** | 0.779 (0.745;0.811) | [15] |
| **Cost** | **Euro 2024 (2007 EUR)** |  |
| **Year 1 first incidence** | 11,923 (8,585) | [2] |
| **Year 1 recurrence** | 12,996 (9,358) | [2] |
| **Year 2-6** | 543 (391) | [2] |
| **Year 6-10** | 219 (158) | [2] |

#### 3.3.2 - Colorectum cancer

The QoL-weights for colorectum cancer were retrieved from a study conducted in the United States of America and estimated using EQ-5D [16]. The study estimated weights for the first year after developing colorectum cancer (see Supplementary Table 8). We have assumed that QoL is affected the following four years with a linear increase of the weight to 1.

The cost associated with colorectum cancer was based on a study conducted in Canada and presented in Supplementary Table 8 [17]. In the referenced study, costs were originally reported in Canadian dollar adjusted to the 2007 price index.

Supplementary Table 8 - Cost and QoL-weights for colorectum cancer.

| **Colorectum cancer** | | | **Reference** |
| --- | --- | --- | --- |
| **QoL-weights** | | **Estimate (95% CI)** |  |
| **Stage 1 first year** | | 0.8339 (0.8072;0.8605) | [16] |
| **Stage 2 first year** | | 0.7833 (0.7539;0.8128) | [16] |
| **Stage 3 first year** | | 0.7310 (0.7096;0.7524) | [16] |
| **Stage 4 first year** | | 0.6001 (0.5668;0.6347) | [16] |
| **Cost** | **Year 1 Euro 2024 (2007 CAD)** | **Year 2-5 Euro 2024 (2007 CAD)** |  |
| **Stage 1** | 13,340 (13,734) | 489 (503) | [17] |
| **Stage 2** | 26,317 (27,095) | 832 (857) | [17] |
| **Stage 3** | 34,034 (35,040) | 832 (857) | [17] |
| **Stage 4** | 70,206 (72,281) | 651 (670) | [17] |

#### 3.3.3 - Oesophageal cancer

The QoL-weights for oesophageal cancer were retrieved from a Canadian study and estimated using EQ-5D [18]. The weights in Supplementary Table 9 apply in the first year, after which we have assumed that QoL is affected in the following four years with a linear increase of the weights to 1.

The cost associated with oesophageal cancer was based on a study conducted in the United Kingdom and presented in Supplementary Table 9. In the referenced study, costs were originally reported in Euro adjusted to the 2024 price index.

Supplementary Table 9 - Cost and QoL-weights for oesophagus cancer.

| **Oesophageal** **cancer** | | **Reference** |
| --- | --- | --- |
| **QoL-weights** | **Estimate (SD)** |  |
| **Stage 1 first year** | 0.78 (0.15) | [18] |
| **Stage 2/3 first year** | 0.82 (0.13) | [18] |
| **Stage 4 first year** | 0.74 (0.18) | [18] |
| **Cost (first year)** | **Euro 2024** |  |
| **Stage 1** | 28,257 | [19] |
| **Stage 2** | 39,909 | [19] |
| **Stage 3** | 32,753 | [19] |
| **Stage 4** | 31,788 | [19] |

#### 3.3.4 - Liver cancer

The QoL-weights for liver cancer were retrieved from a Canadian study and estimated using EQ-5D [20]. The weights in Supplementary Table 10 apply in the first year, after which we have assumed that QoL is affected the following four years with a linear increase of the weights to 1.

The cost associated with liver cancer was based on a Swedish study and presented in Supplementary Table 10 [21]. Similar to the weights, we consider costs for liver cancer up to year 5. In the referenced study, costs were originally reported in Euro adjusted to the 2021 price index.

Supplementary Table 10 - Cost and QoL-weights for liver cancer.

| **Liver cancer** | | **Reference** |
| --- | --- | --- |
| **QoL-weights** | **Estimate (95% CI)** |  |
| **First year** | 0.65 (0.44;0.86) | [20] |
| **Cost** | **Euro 2024 (2021 EUR)** |  |
| **First year** | 11,951 (10,465) | [21] |
| **Following years (2-4)** | 1,961 (1,717) | [21] |

#### 3.3.5 - Oral cancer

The QoL-weights for oral cancer were retrieved from a study from the United Kingdom and estimated using EQ-5D [22]. The weights in Supplementary Table 11 apply in the first year, after which we have assumed that QALY is affected the following four years with a linear increase of the weights to 1.

The cost associated with oral cancer was based on the same study from the United Kingdom and presented in Supplementary Table 11 [22]. The study reports costs for the first three years, however, we consider costs for oral cancer up to year 5, assuming that costs for years 4 and 5 are the same as those for year 3. In the referenced study, costs were originally reported in British pounds and adjusted to the 2001 price index.

Supplementary Table 11 - Cost and QoL-weights for oral cancer.

| **Oral cancer** | | | | **Reference** |
| --- | --- | --- | --- | --- |
| **QoL-weights** | | | **Estimate (SD)** |  |
| **Stage 1 - First year** | | | 0.88 (0.20) | [22] |
| **Stage 2, 3 and 4 - First year** | | | 0.68 (0.33) | [22] |
| **Cost** | **Year 1 Euro 2024 (2001 GBP)** | **Year 2 Euro 2024 (2001 GBP)** | **Year 3 Euro 2024 (2001 GBP)** |  |
| **Stage 1** | 8,306 (3,948) | 1,161 (552) | 871 (414) | [22] |
| **Stage 2** | 14,986 (7,123) | 1,475 (701) | 1,477 (702) | [22] |
| **Stage 3** | 21,556 (10,246) | 1,572 (747) | 1,660 (789) | [22] |
| **Stage 4** | 20,818 (9,895) | 3,894 (1,851) | 3,720 (1,768) | [22] |

#### 3.3.6 - Pancreas cancer

The QoL-weights for pancreas cancer were retrieved from a study from Canada and estimated using EQ-5D [23]. The weights in Supplementary Table 12 apply in the first year, after which we have assumed that QoL is affected the following four years with a linear increase of the weights to 1. The literature on QoL related to pancreatic conditions is sparse and variable. Previous cost-effectiveness analyses have not always incorporated a specific weight due to the limited research available. Instead, they have relied solely on population-weighted estimates. The weights in Supplementary Tab2 are intended for use when the disease is in a stable state. However, this utility is applied even when conditions may not be stable, and individuals still have an increased mortality risk.

The cost associated with pancreas cancer was based on a study from the United States of America and presented in Supplementary Table 12 [24]. The cost in the referenced article were reported per month after the first year. Since we use 1-year cycles we converted these into costs that occurs every following full year. Costs were originally reported in US dollars adjusted to the 2019 price index. presents both the original costs and the updated figures.

Supplementary Table 12 - Cost and QoL-weights for pancreas cancer.

| **Pancreas cancer** | | | **Reference** |
| --- | --- | --- | --- |
| **QoL-weights** | | **Estimate (SD)** |  |
| **First year** | | 0.72 (0.185) | [23] |
| **Cost** | **Year 1 Euro 2024 (2019 USD)** | **Following months  Euro 2024 (2019 USD)** |  |
| **Stage 1 and 2** | 7,816 (6,900) | 1,331 (1,175) | [24] |
| **Stage 3** | 11,941 (10,542) | 2,086 (1,842) | [24] |
| **Stage 4** | 10,393 (9,175) | 3,832 (3,383) | [24] |

#### 3.3.7 - HAemorrhagic and ischemic stroke

The QoL-weights for haemorrhagic and ischemic stroke were retrieved from a study from the United Kingdom estimated using EQ-5D [25]. The QoL-weights are presented in Supplementary Table 13. Specific weights were reported for years 1, 2, and 5, and we used year 2 weights for years 3 and 4. The study also reported a QoL-weight for the remaining life years of the individual.

The cost associated with ischemic and haemorrhagic stroke was based on a Swedish study and presented in Supplementary Table 13 [26]. The costs for the second year are estimated to continue throughout life. If an individual experience another stroke, the costs would recur for the first year and then return to second year costs. In the referenced study, costs were originally reported in Euro and adjusted to the 2016 price index.

Supplementary Table 13 - Cost and QoL-weights for ischemic and haemorrhagic stroke

| **Ischemic and haemorrhagic stroke** | | **Reference** |
| --- | --- | --- |
| **QoL-weights** | **Estimate (SD)** |  |
| **Haemorrhagic stroke** |  |  |
| **First year** | 0.70 (0.27) | [25] |
| **Second year (used for third and fourth)** | 0.66 (0.29) | [25] |
| **Fifth year** | 0.56 (0.34) | [25] |
| **Annual for life** | 0.81 | [26] |
| **Ischemic stroke** |  |  |
| **First year** | 0.67 (0.36) | [25] |
| **Second year (used for third and fourth)** | 0.81 (0.18) | [25] |
| **Fifth year** | 0.79 (0.25) | [25] |
| **Annual for life** | 0.96 | [26] |
| **Cost** | **Euro 2024 (2016 EUR)** |  |
| **Haemorrhagic stroke** |  |  |
| First year | 45,593 (37,788) | [26] |
| Second year | 5,685 (4,712) | [26] |
| **Ischemic stroke** |  |  |
| First year | 23,980 (19,875) | [26] |
| Second year | 5,857 (4,854) | [26] |

#### 3.3.8 - Myocardial infarcation

The QoL-weights for myocardial infarction were retrieved from a study from Sweden and estimated using EQ-5D [27]. The weights are presented in Supplementary Table 14. Specific weights were assigned to different age groups for the first year. In following years, the same weights are applied across all age groups.

The costs associated with myocardial infarction was based on a Swedish study and presented in Supplementary Table 14 [27]. The costs are estimated to occur in the first year, and costs for following years are expected to continue until death. In the referenced study, costs were originally reported in Euro and adjusted to the 2012 price index.

Supplementary Table 14 - Cost and QALY for myocardial infarction.

| **Myocardial infarction** | | **Reference** |
| --- | --- | --- |
| **QoL-weights** | **Estimate** |  |
| **Age < 69 - First year** | 0.8748 | [27] |
| **Age 70-79 - First year** | 0.8430 | [27] |
| **Age > 80 - First year** | 0.7814 | [27] |
| **Following years** | 0.9373 | [27] |
| **Cost** | **Euro 2024 (2012 EUR)** |  |
| **First year** | 19,622 (15,656) | [27] |
| **Following years** | 5,229 (4,172) | [27] |

#### 3.3.9 - Alcohol-related liver disease

The QoL-weights for alcohol-related liver disease were retrieved from a Canadian study and estimated using EQ-5D [20]. It is not possible to fully recover from alcohol-related liver disease; instead, there is a decrease in quality of life each year. The weight in Supplementary Table 15 applies to both the first year and the subsequent years.

The costs associated with alcohol-related liver disease were estimated in a Swedish setting [21] and presented in Supplementary Table 15. In the referenced study, costs were originally reported in Euro and adjusted to the 2021 price index.

Supplementary Table 15 - Cost and QALY for alcohol-related liver disease

| **Alcohol-related liver disease** | | **Reference** |
| --- | --- | --- |
| **QoL-weights** | **Estimate (95% CI)** |  |
| **Alcohol-related liver disease** |  |  |
| Per year | 0.74 (0.66;0.83) | [20] |
| **Cost** | **Euro 2024 (2021 EUR)** |  |
| **Alcohol-related disease** |  |  |
| Per year | 4,343 (3,803) | [21] |

## 4 - Analysis and validation

One of the objectives of this study was to develop a baseline version of the individual-level simulator. Part of doing so was to show that the output from the individual-level simulations is congruent with the parameters entered, and our expectations of how disease and mortality should develop in a population over time. For this purpose, we ran simulations of a population consisting of 100,000 men and 100,000 women, 0 to 100 years of age, for 100 years.

Unlike deterministic cohort models, including most Markov models, two runs of our simulator do not result in the same results. There are two main reasons for this. First, outcomes are probabilistic throughout the simulations, for instance, if there is a 1% probability that an individual will die in a given year, then we sample from a Bernoulli distribution to decide if the person died or not. This *Monte Carlo* *error* is reduced if the simulated population is large enough, however, there will always be small differences due to this. The second and larger source of variation comes from the input parameters. The simulator incorporates uncertainties regarding incidence, mortality, cost, and QoL throughout simulations, and this uncertainty propagates into the results. Therefore, the simulator should be run multiple times and outcomes analysed across simulations. Thus, by design, a probabilistic sensitivity analysis is included in the running of the simulator. For our analyses in this study, we aggregated outcomes across 5,000 simulations.

## 5 - Baseline results

### 5.1 - incidence of disease

In Supplementary Figure 3 Panel A, we have plotted the derived annual log incidence rate for breast cancer used as parameters in the simulator based on data published by the Swedish National Board of Health and Welfare (Input parameters). The plot also contains the annual log incidence rate among simulated individuals (Simulated). Mean log incidence rates are presented as points, and bars represent the interquartile range. Panels B to J of Supplementary Figure 3 presents the same data for each of the diseases in the simulator. As can be seen, there is little difference between input parameters and simulated data, suggesting that the incidence rate of diseases among simulated individuals is as expected. It should be noted that the incidence rate for liver cancer is slightly higher in the simulated outcomes due to the model accounting for progression from alcohol-related liver disease to liver cancer, thus, the increased incidence rate is expected.

| Panel A – Breast cancer |
| --- |
| 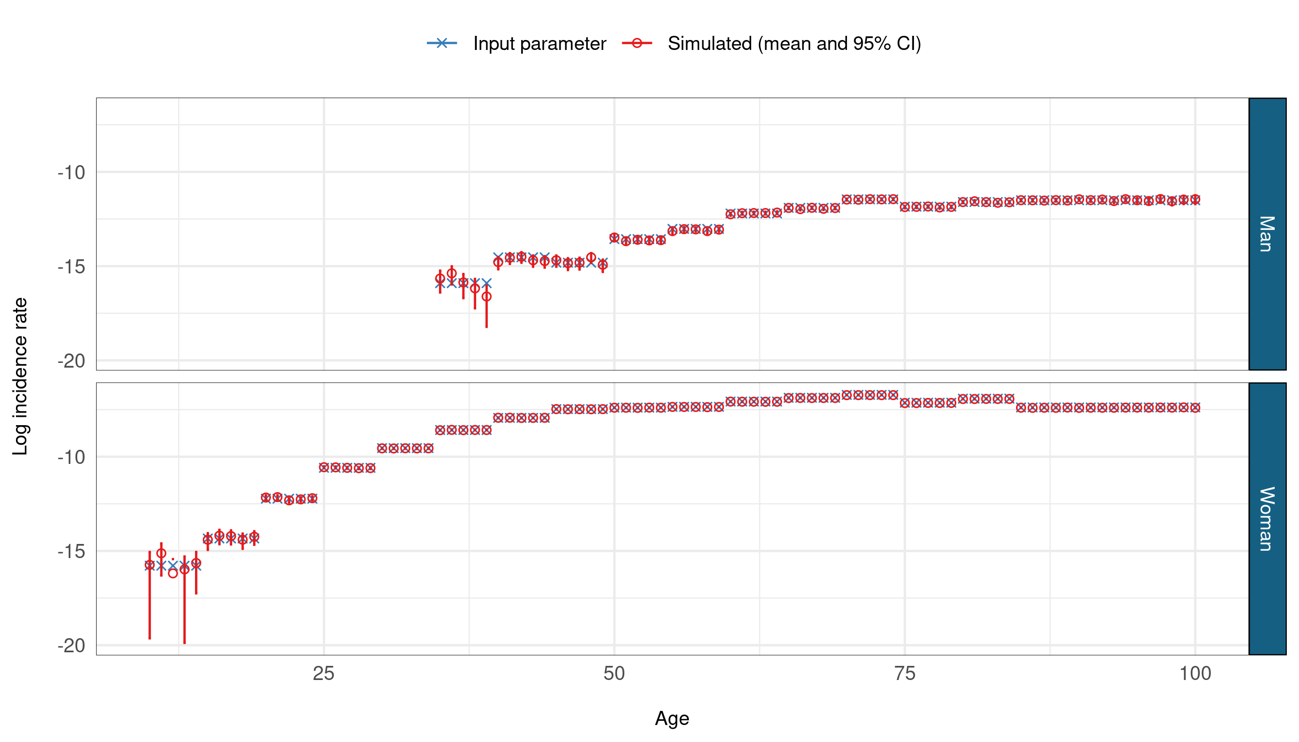 |
|  |
| Panel B – Colorectum cancer |
| 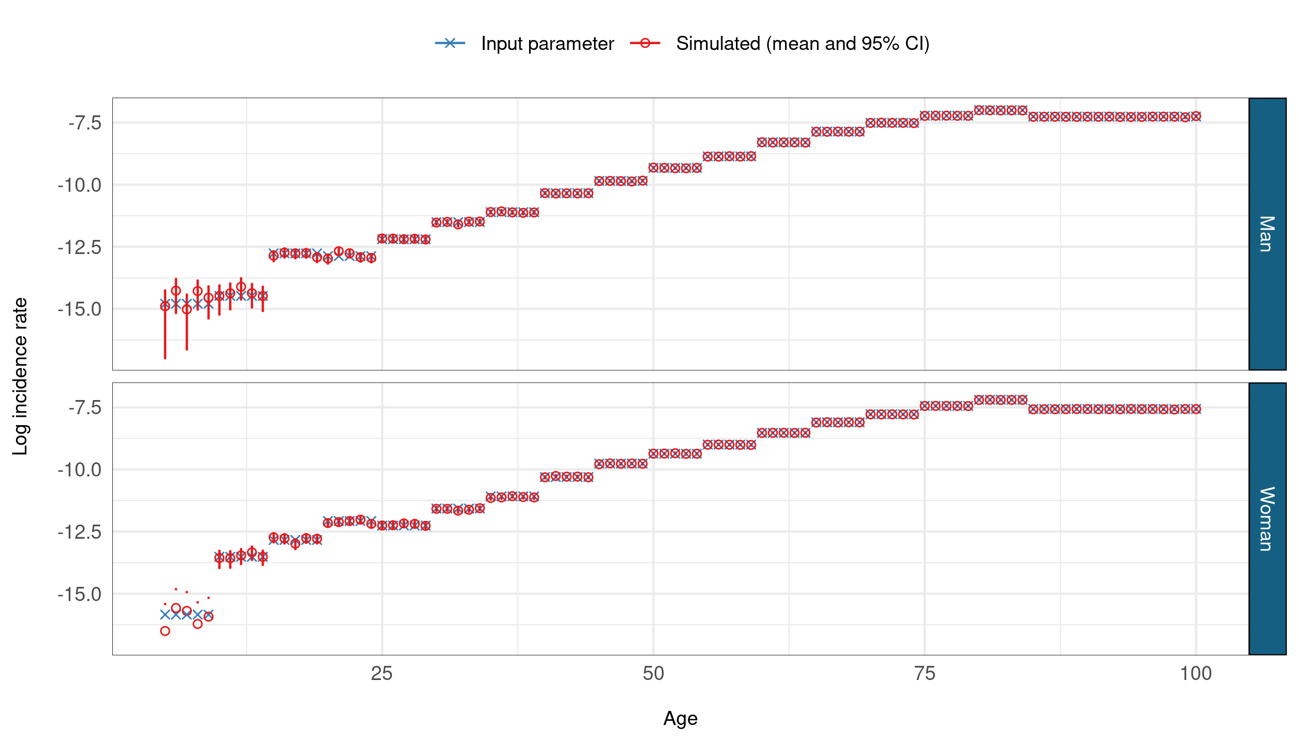 |
| Panel C – Oesophageal cancer |
| 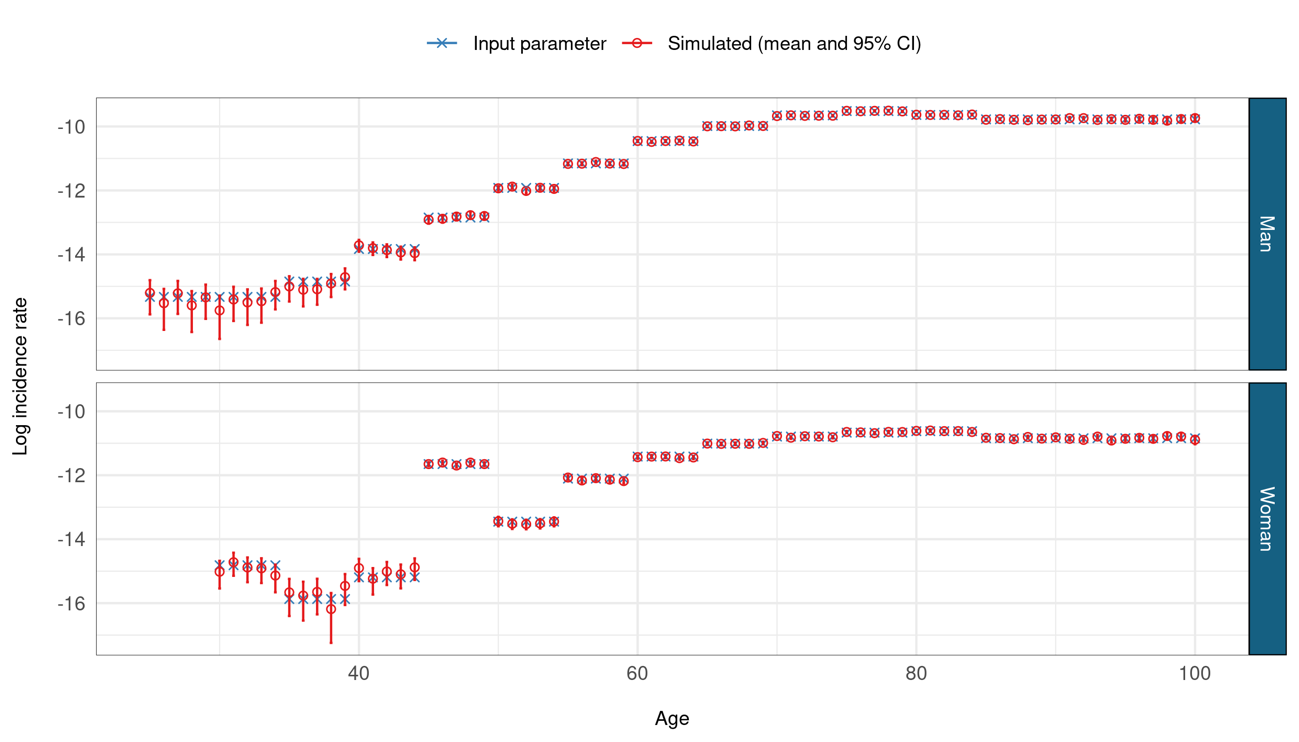 |
|  |
| Panel D – Liver cancer |
| 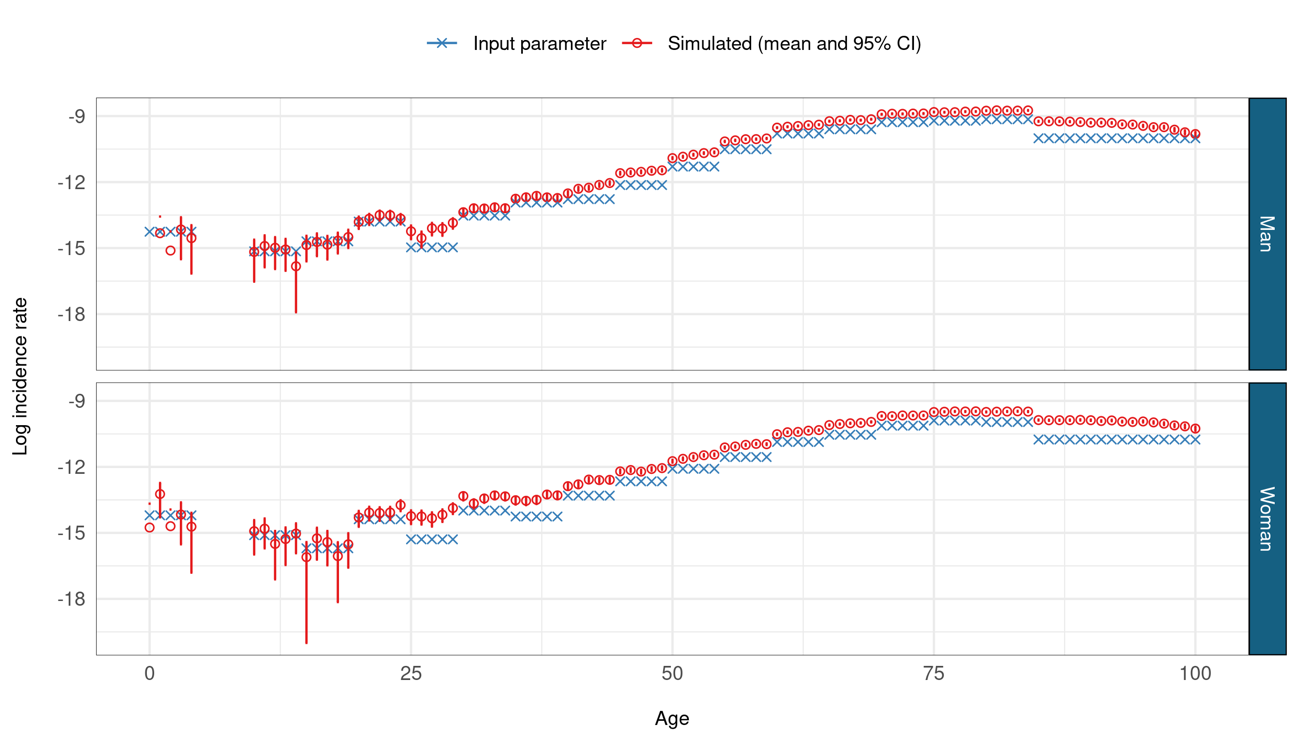 |
| Panel E – Oral cancer |
| 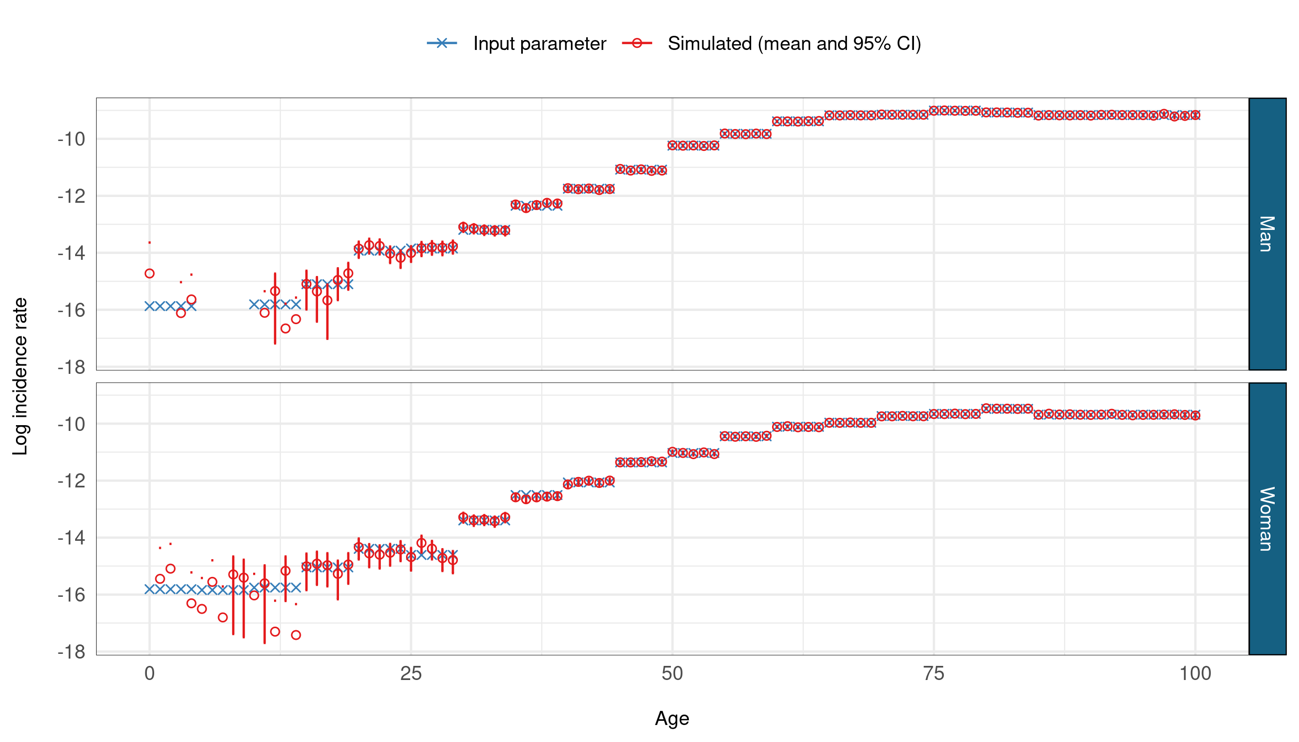 |
|  |
| Panel F – Pancreas cancer |
| 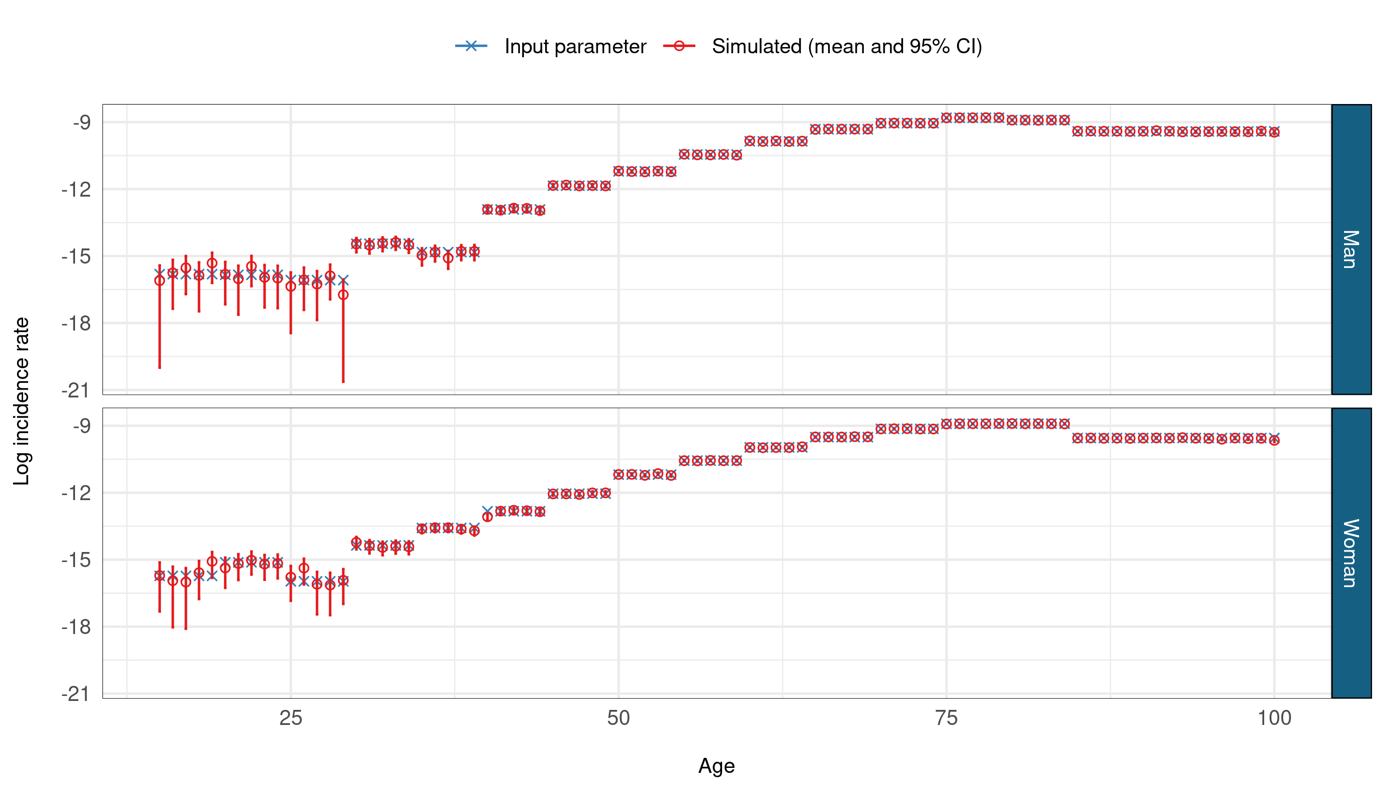 |
| Panel G– Ischemic stroke |
| 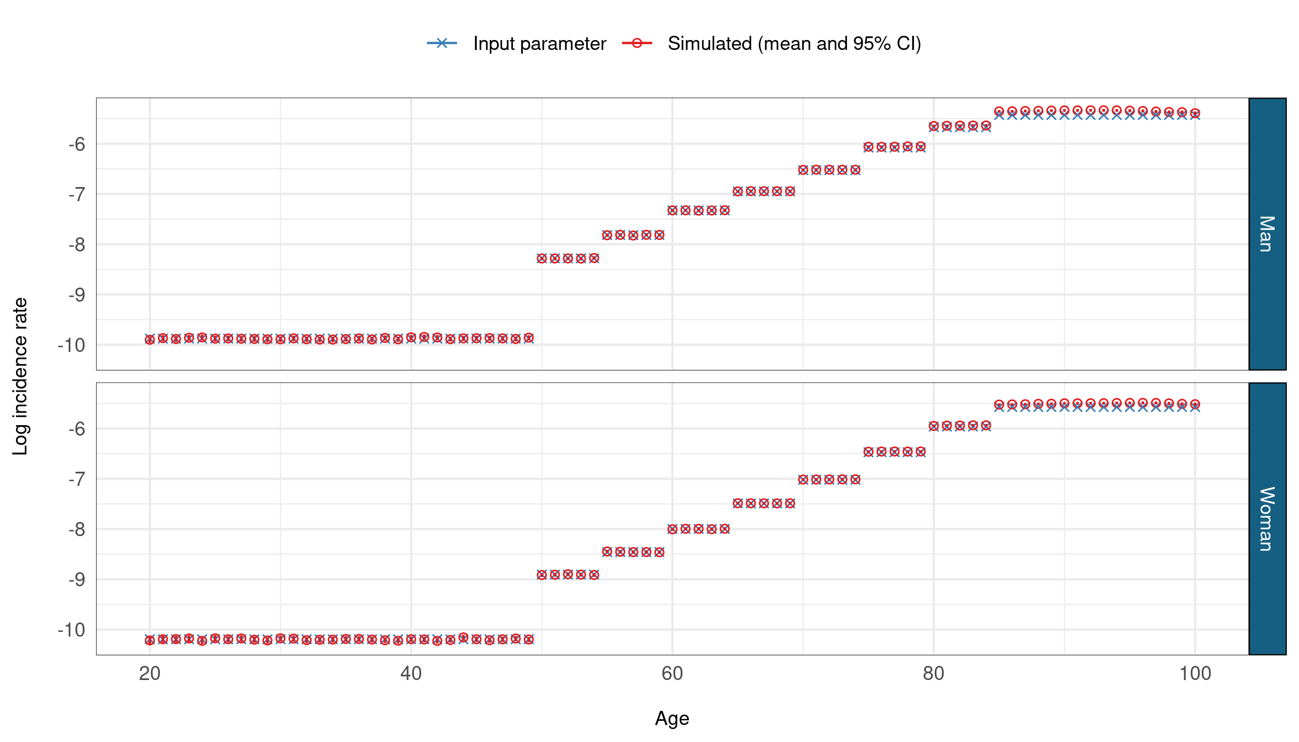 |
|  |
| Panel H – Haemorrhagic stroke |
| 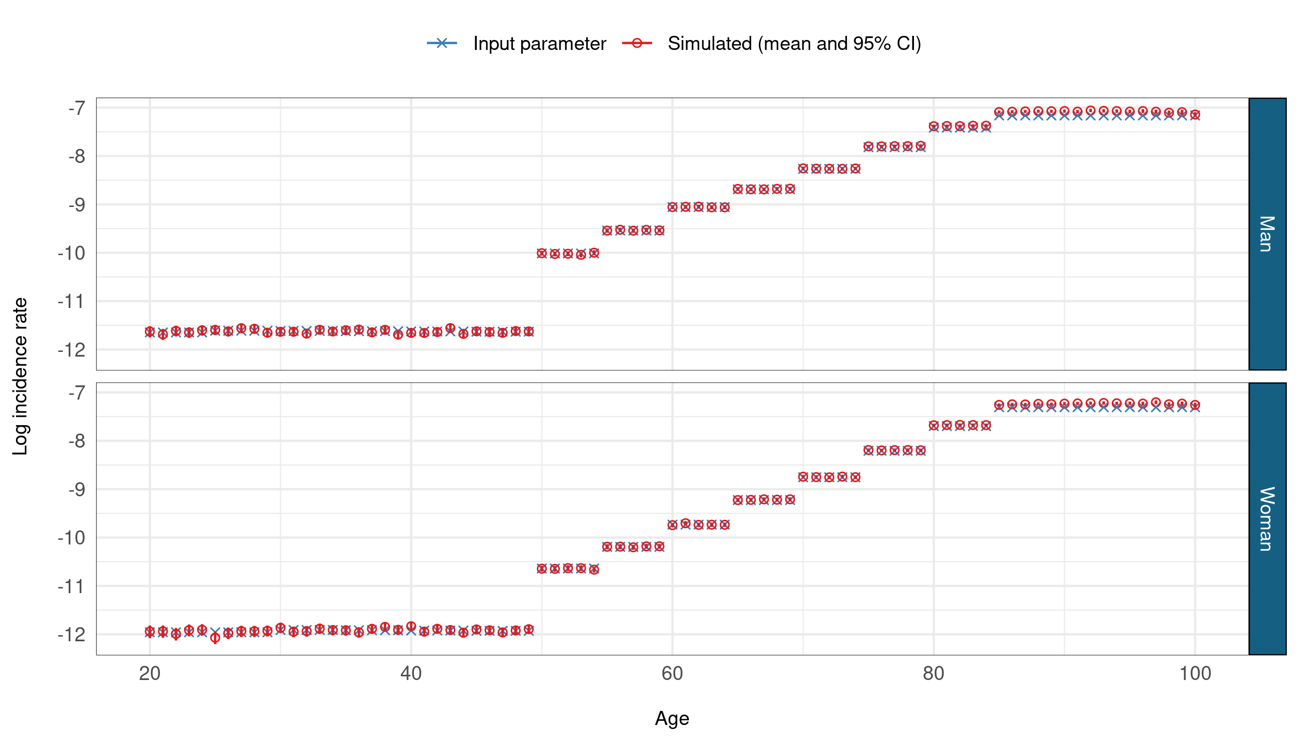 |
| Panel I – Myocardial infarction |
| 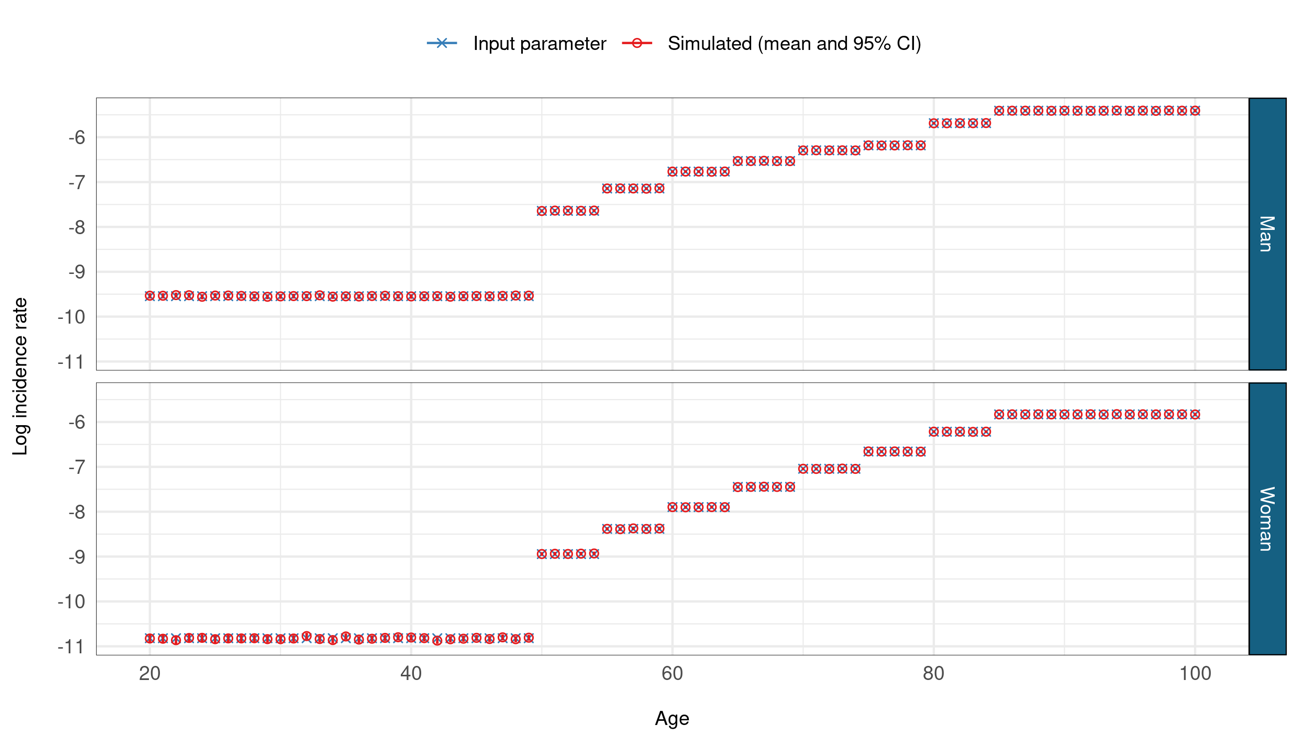 |
|  |
| Panel J – Alcohol-related liver disease |
| 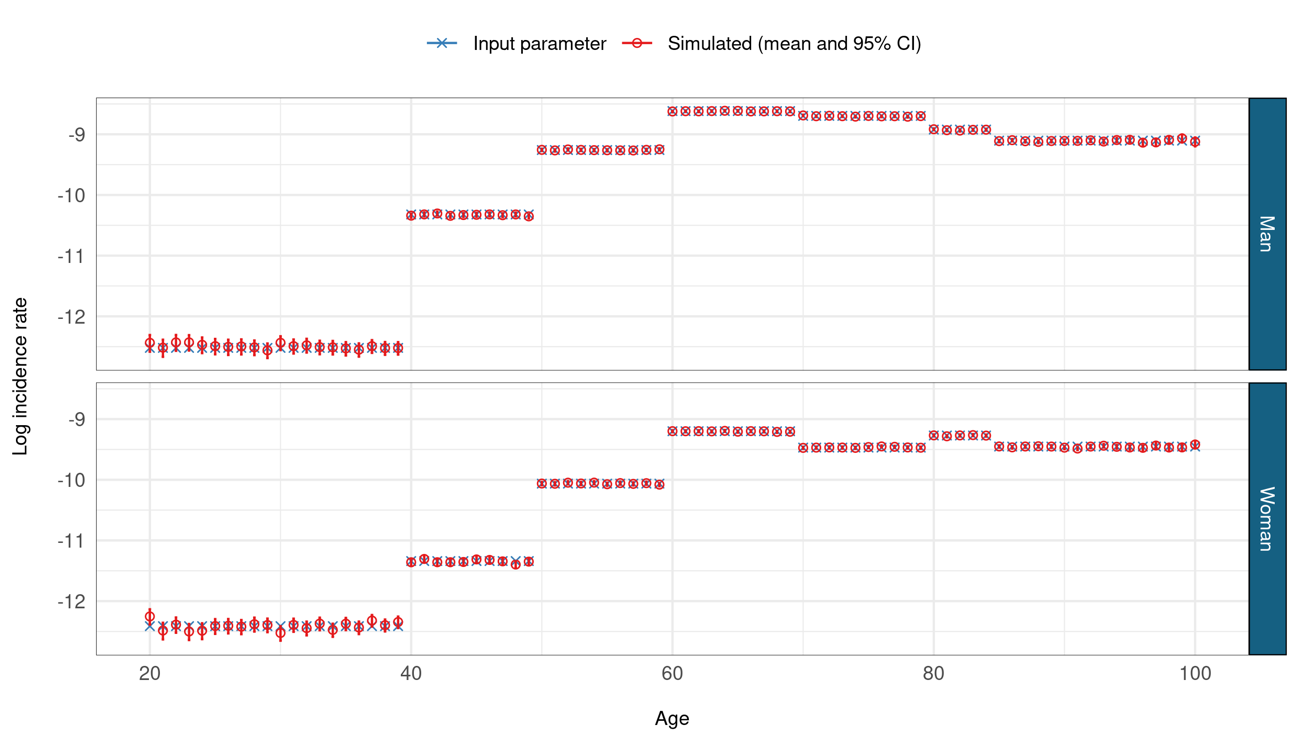 |

Supplementary Figure 3 - Comparing input parameters (Input parameter), and the incidence rate among simulated individuals (Simulated). Incidence rates are on the log scale. Points represent the mean and bars +/- 1.96 standard errors.

### 5.2 - non-disease specific mortality

In Supplementary Figure 4, we have plotted non-disease specific mortality data from Statistics Sweden (Input data), the derived input parameters from these data, i.e., average across the years (Input parameters), and the probability of dying of the simulated individuals (Simulated).


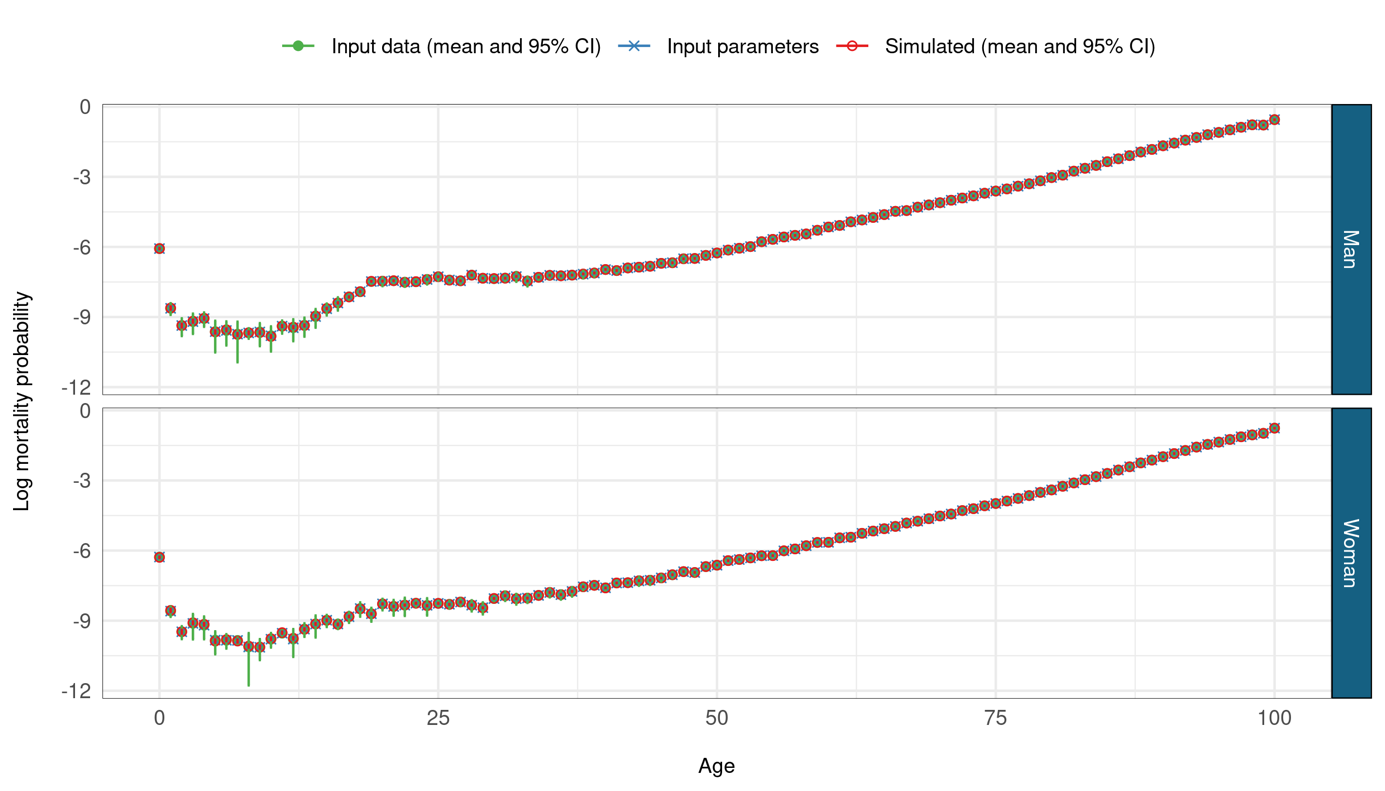


Supplementary Figure 4 - Comparing non-disease specific mortality data from Statistics Sweden (Input data), with derived parameters from these data (Input parameters), and the probability of dying of simulated indivudals (Simulated). Mortality probabilities are on the log scale. Points represents means and bars +/- 1.96 standard errors.

### 5.3 - Disease specific mortality

In Supplementary Figure 5 Panel A, we have, for breast cancer, plotted the annual mortality probability calculated by aggregating cases and deaths across years (Input). We have also plotted the input parameters estimated from the input data using natural cubic spline logistic regression (Input parameters). Finally, the simulated disease specific mortality probabilities are plotted (Simulated). Mortality probabilities are plotted on the log scale. The plots show that mortality rates among the simulated individuals due to the modelled diseases are as expected, with few cases of mortality in younger ages due to the low incidence rate of these diseases in young individuals.

| Panel A – Breast cancer |
| --- |
| 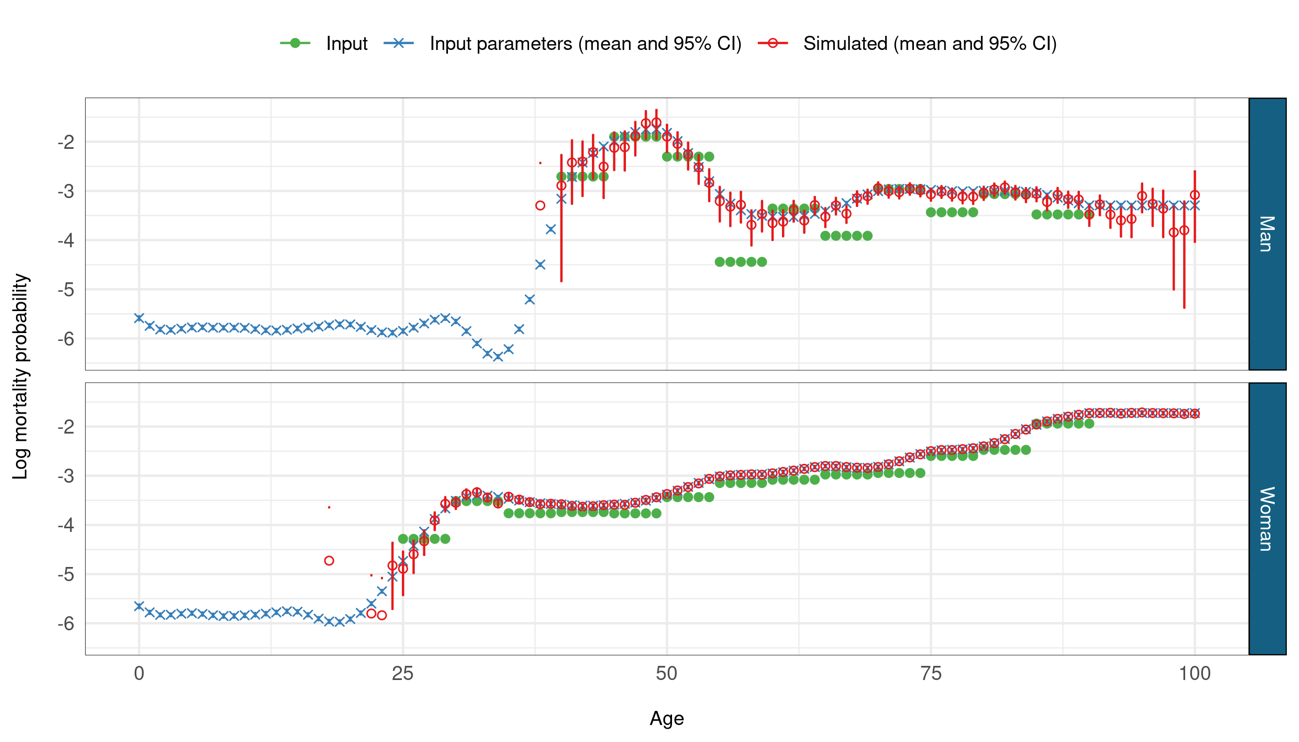 |
|  |
| Panel B – Colorectum cancer |
| 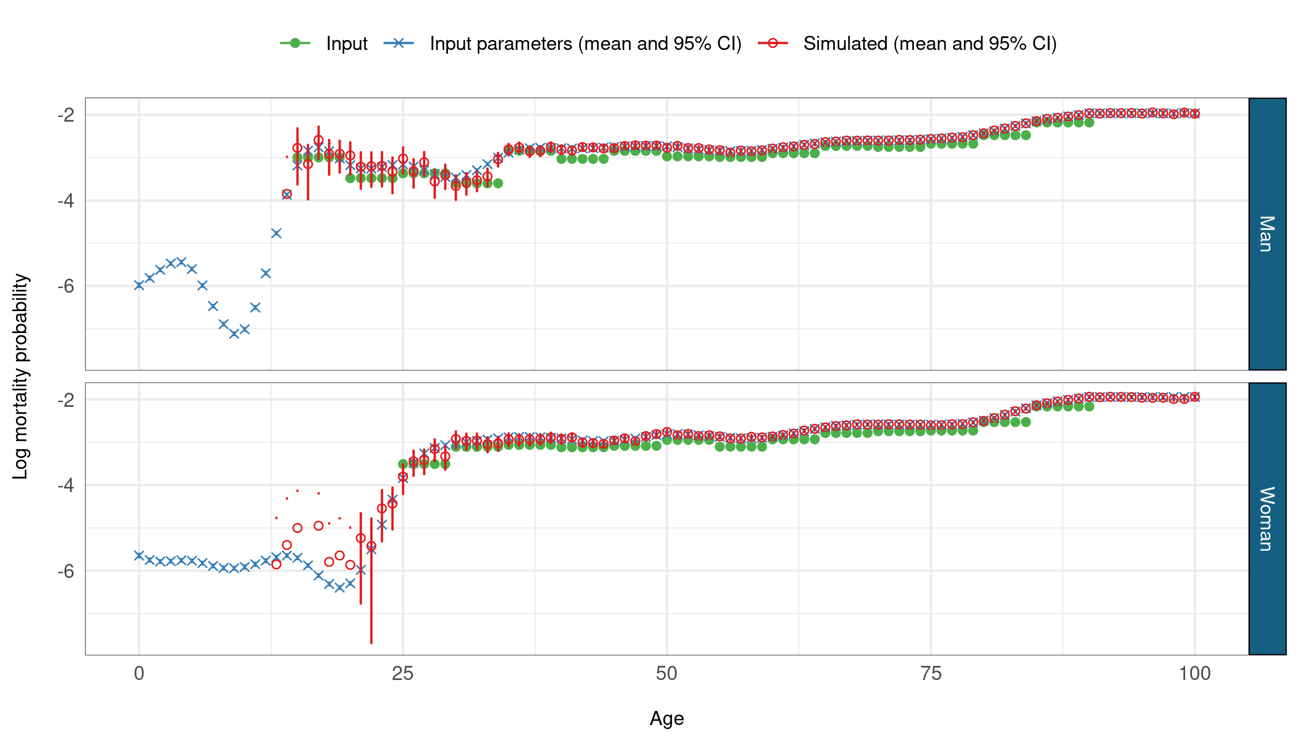 |
| Panel C – Oesophageal cancer |
| 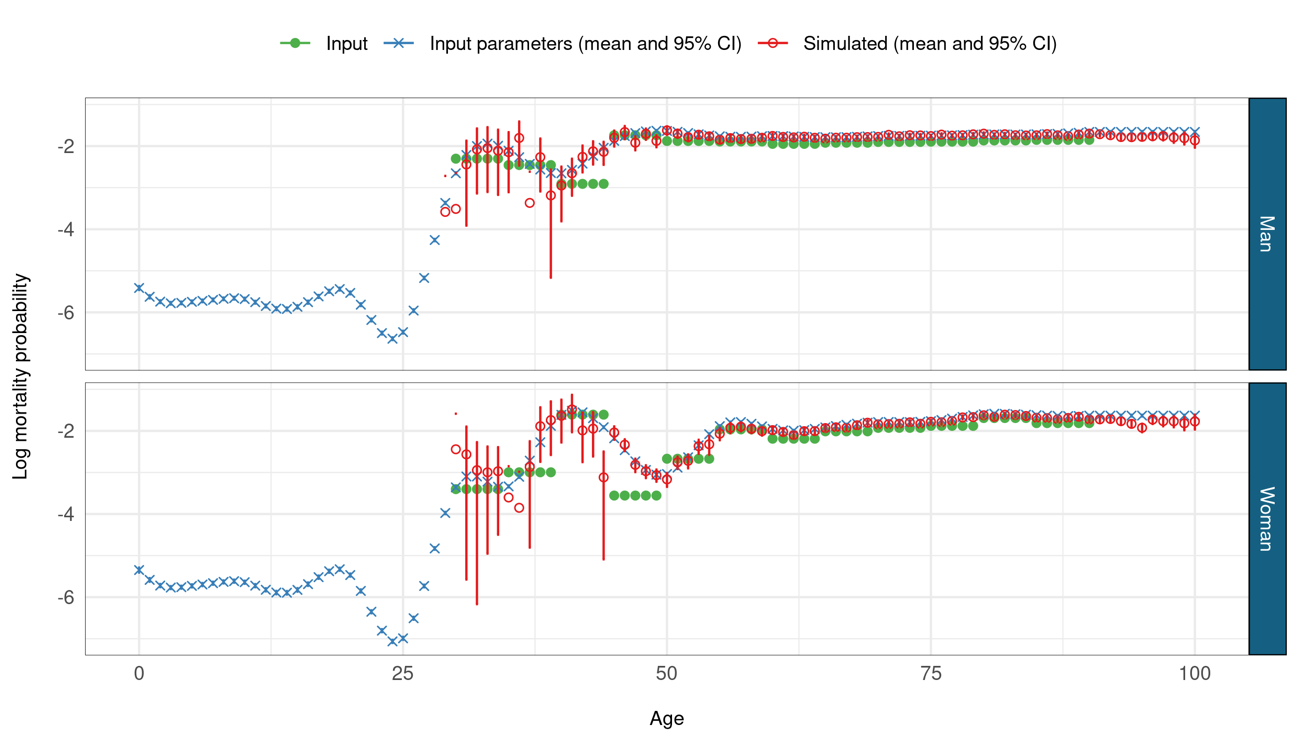 |
|  |
| Panel D – Liver cancer |
| 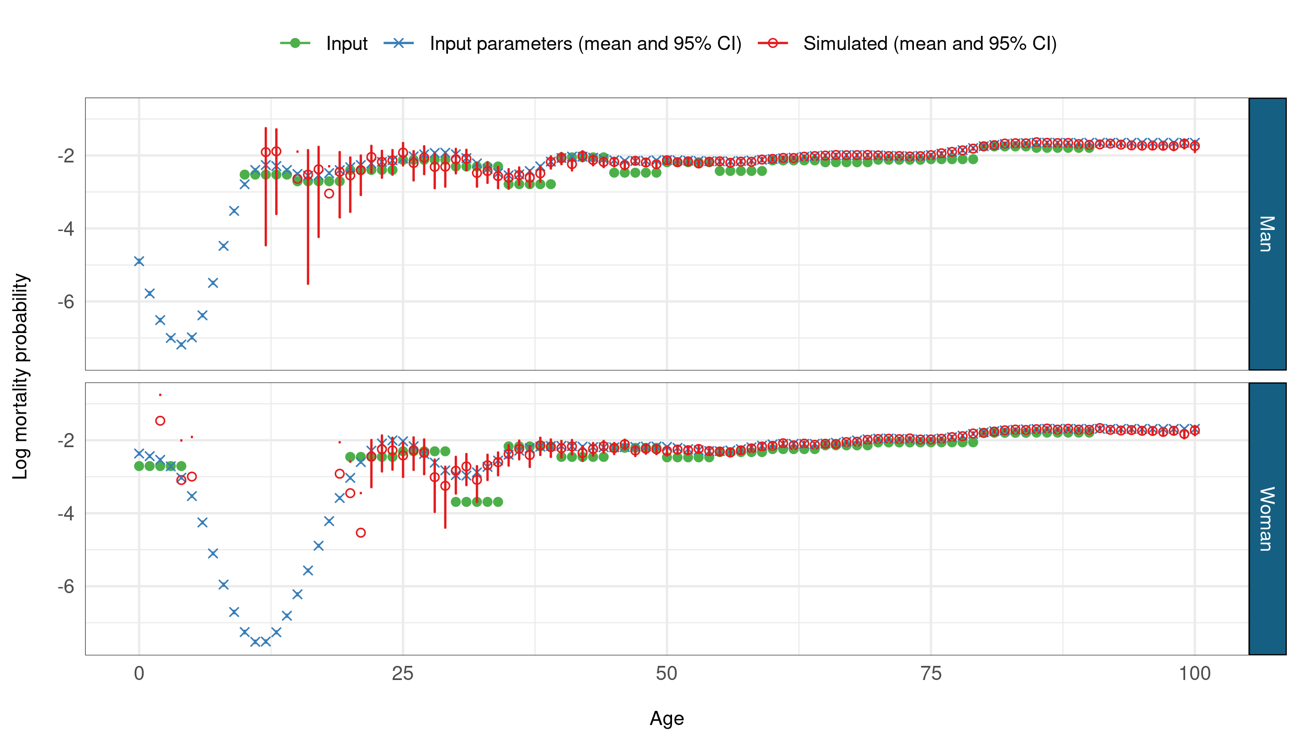 |
| Panel E – Oral cancer |
| 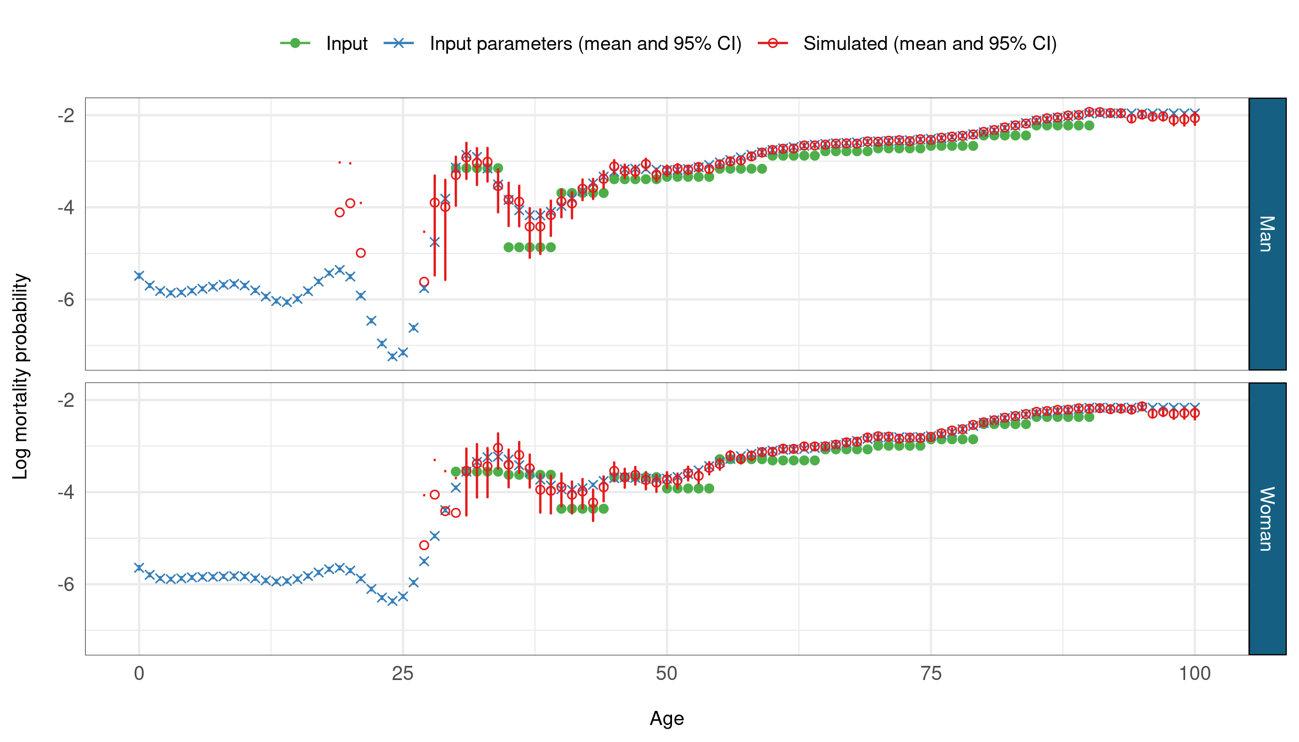 |
|  |
| Panel F – Pancreas cancer |
| 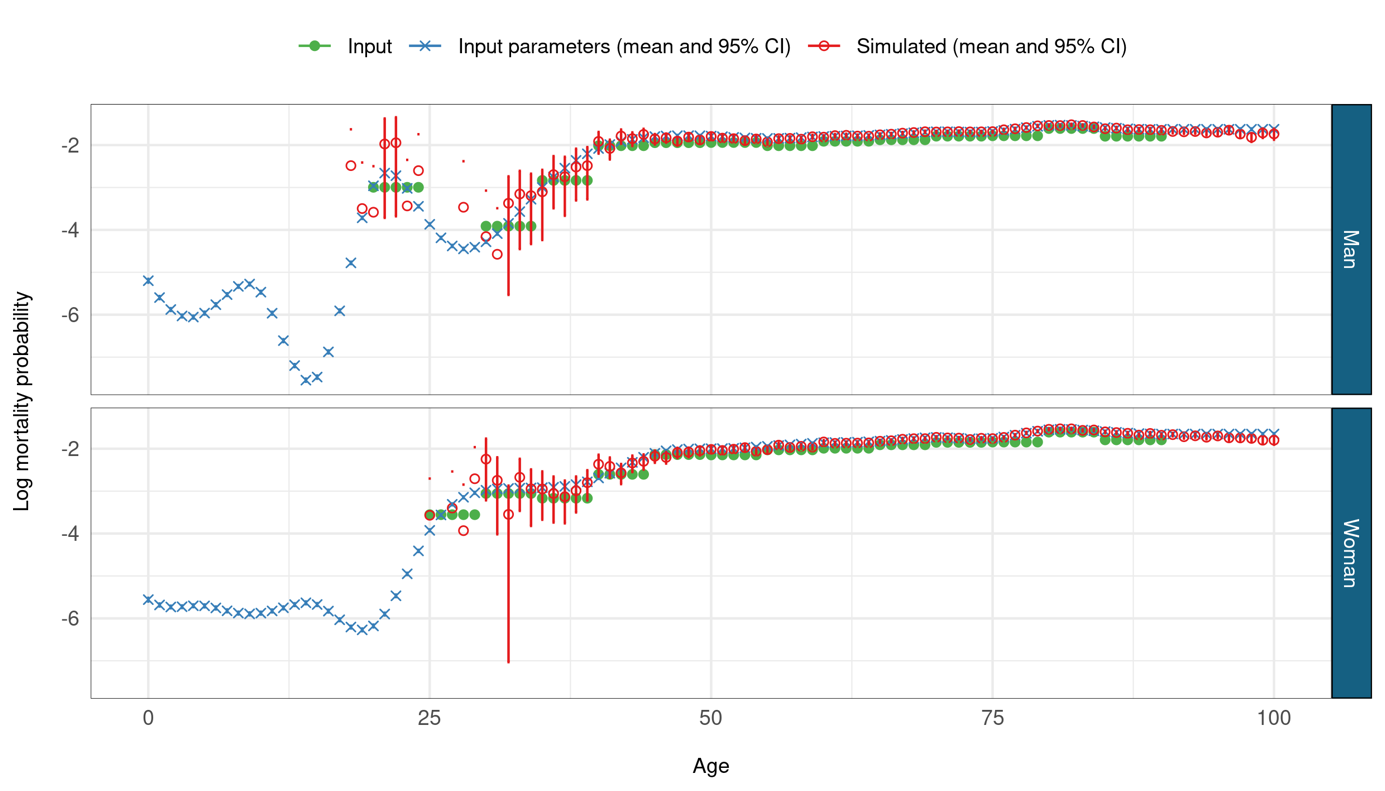 |
| Panel G – Ischemic stroke |
| 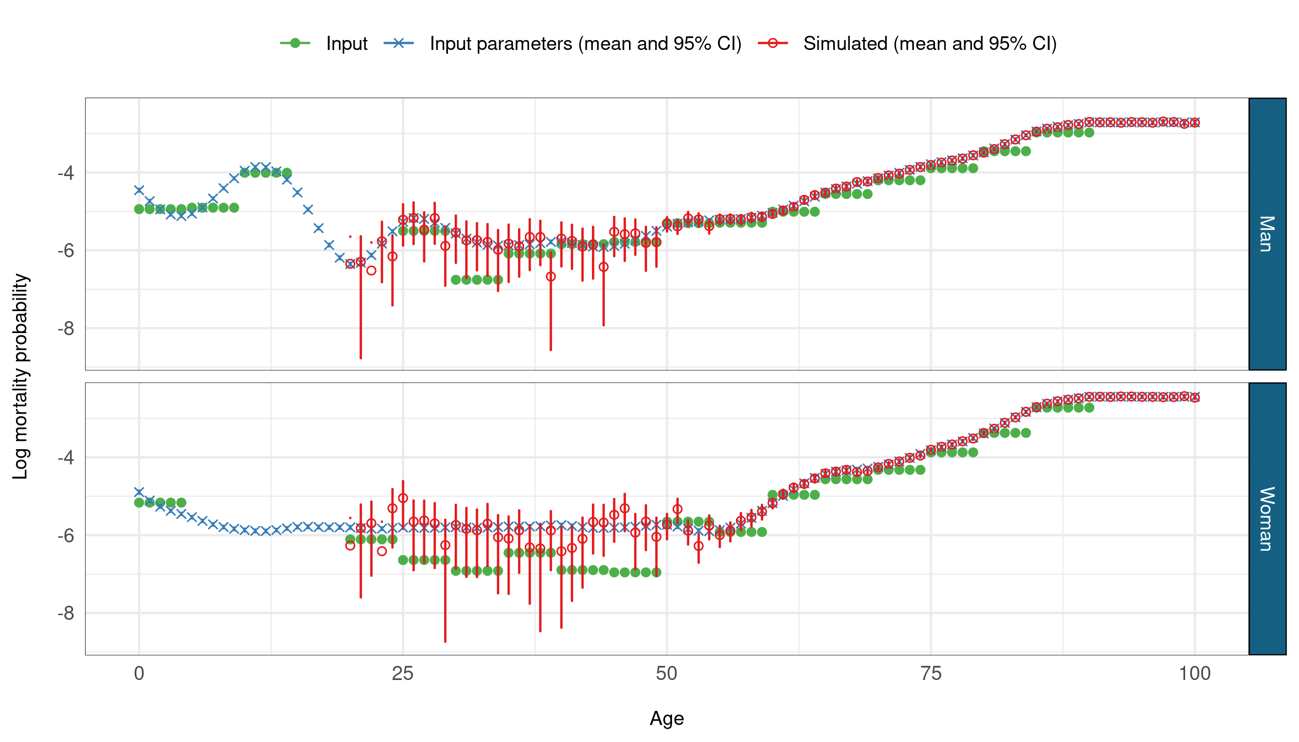 |
|  |
| Panel H – Haemorrhagic stroke |
| 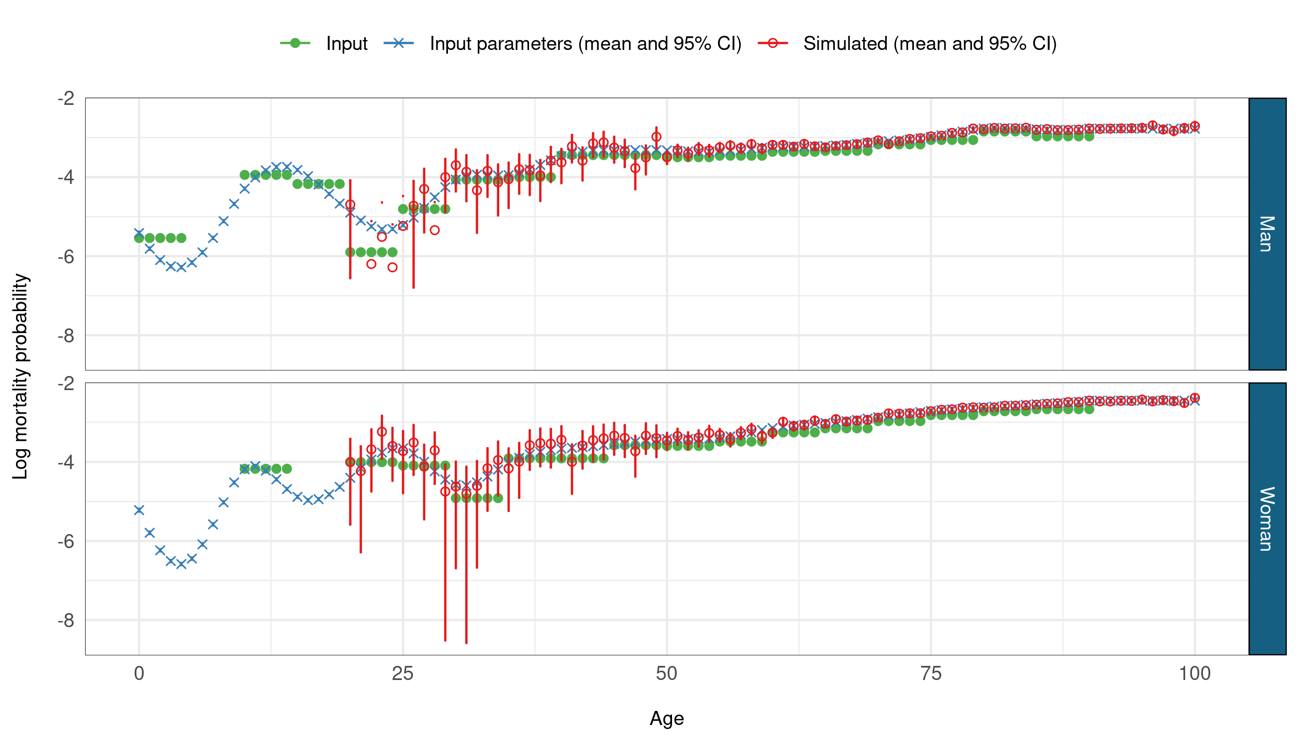 |
| Panel I – Myocardial infarction |
| 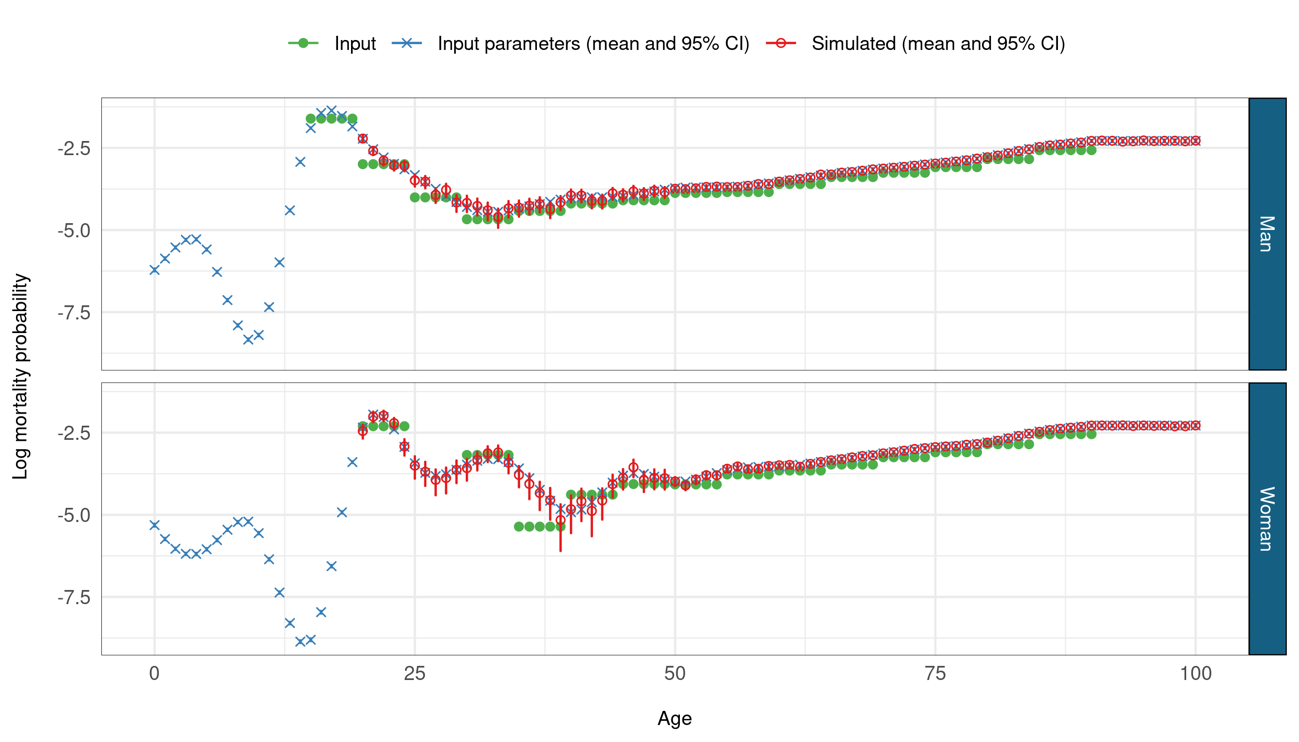 |
|  |
| Panel J – Alcohol-related liver disease |
| 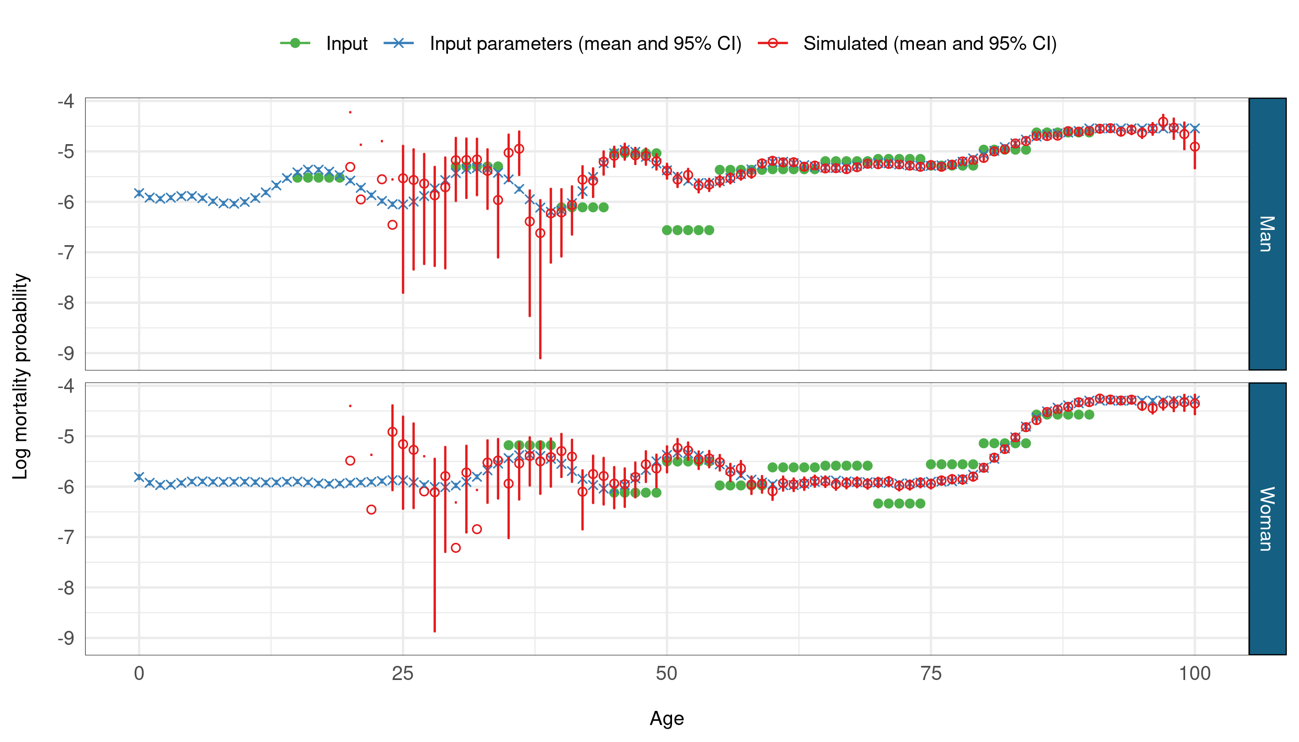 |

Supplementary Figure 5 - Annual mortality probability calculated by aggregating cases and deaths across years (Input). Input parameters estimated from the input data using natural cubic spline logistic regression (Input parameters). Simulated disease specific mortality probabilities are plotted (Simulated). Mortality probabilities are plotted on the log scale, with points representing means and bars +/- 1.96 standard errors.

### 5.4 - Health related quality-of-life

In Panel A of Supplementary Figure 6, we have plotted lifetime accumulated QALY against age at the beginning of the simulation. For instance, we can see that individuals aged 0 at the start of the simulation accumulated on average approximately 30 discounted QALYs over their lifetime.

| Panel A – Discounting QALY 3% annually |
| --- |
| **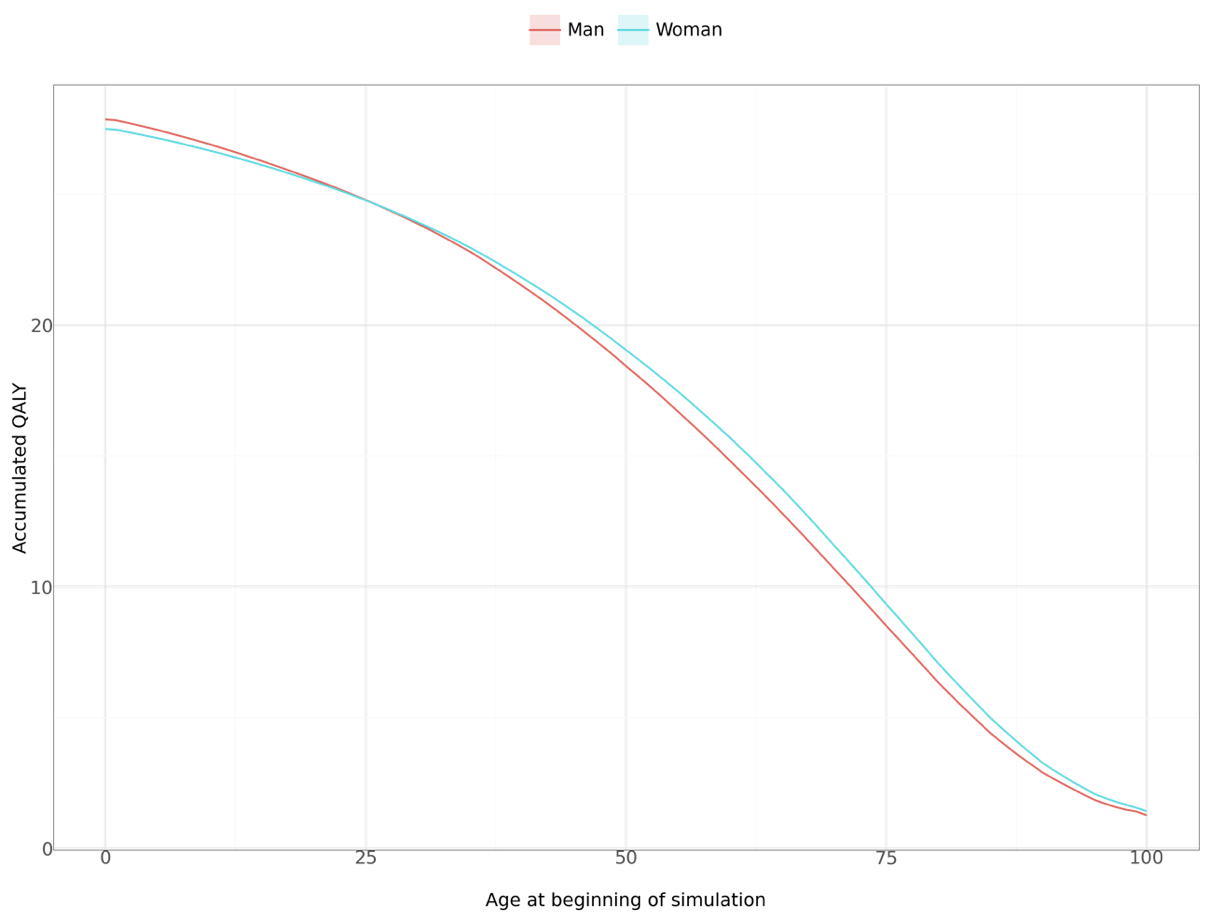** |

Supplementary Figure 6 – Lifetime accumulated QALY plotted against age at the beginning of the simulation.

# References

1. Vaz J, Eriksson B, Strömberg U, Buchebner D, Midlöv P. Incidence, aetiology and related comorbidities of cirrhosis: a Swedish population-based cohort study. BMC Gastroenterology. 2020;20(1):84.

2. Lundkvist J, Wilking N, Holmberg S, Jönsson L. Cost-effectiveness of exemestane versus tamoxifen as adjuvant therapy for early-stage breast cancer after 2-3 years treatment with tamoxifen in Sweden. Breast Cancer Res Treat. 2007;102(3):289–99.

3. Lin CW, Lin CC, Mo LR, Chang CY, Perng DS, Hsu CC, et al. Heavy alcohol consumption increases the incidence of hepatocellular carcinoma in hepatitis B virus-related cirrhosis. Journal of Hepatology. 2013;58(4):730–5.

4. Flach C, Muruet W, Wolfe CDA, Bhalla A, Douiri A. Risk and Secondary Prevention of Stroke Recurrence. Stroke. 2020;51(8):2435–44.

5. Leong SPL, Shen ZZ, Liu TJ, Agarwal G, Tajima T, Paik NS, et al. Is Breast Cancer the Same Disease in Asian and Western Countries? World J Surg. 2010 Oct 1;34(10):2308–24.

6. Kodeda K, Nathanaelsson L, Jung B, Olsson H, Jestin P, Sjövall A, et al. Population-based data from the Swedish Colon Cancer Registry. British Journal of Surgery. 2013 Jul 1;100(8):1100–7.

7. Visser O, van Leeuwen FE. Stage-specific survival of epithelial cancers in North-Holland/Flevoland, The Netherlands. European Journal of Cancer. 2005 Oct 1;41(15):2321–30.

8. Engstrand J, Stål P, Gilg S, Jansson A, Strömberg C. Hepatocellular carcinoma in cirrhotic versus non-cirrhotic liver: Treatment and survival differences in a nationwide cohort. Scand J Surg. 2024 Jun 1;113(2):120–30.

9. Geum DH, Roh YC, Yoon SY, Kim HG, Lee JH, Song JM, et al. The impact factors on 5-year survival rate in patients operated with oral cancer. J Korean Assoc Oral Maxillofac Surg. 2013 Oct;39(5):207–16.

10. Kirkegård J, Gaber C, Lund JL, Hinton SP, Ladekarl M, Heide-Jørgensen U, et al. Acute pancreatitis as an early marker of pancreatic cancer and cancer stage, treatment, and prognosis. Cancer Epidemiology. 2020 Feb 1;64:101647.

11. Wood AM, Kaptoge S, Butterworth AS, Willeit P, Warnakula S, Bolton T, et al. Risk thresholds for alcohol consumption: combined analysis of individual-participant data for 599 912 current drinkers in 83 prospective studies. The Lancet. 2018;391(10129):1513–23.

12. Sarich P, Canfell K, Egger S, Banks E, Joshy G, Grogan P, et al. Alcohol consumption, drinking patterns and cancer incidence in an Australian cohort of 226,162 participants aged 45 years and over. Br J Cancer. 2021;124(2):513–23.

13. Askgaard G, Grønbæk M, Kjær MS, Tjønneland A, Tolstrup JS. Alcohol drinking pattern and risk of alcoholic liver cirrhosis: A prospective cohort study. Journal of Hepatology. 2015 May 1;62(5):1061–7.

14. Teni FS, Gerdtham UG, Leidl R, Henriksson M, Åström M, Sun S, et al. Inequality and heterogeneity in health-related quality of life: findings based on a large sample of cross-sectional EQ-5D-5L data from the Swedish general population. Qual Life Res. 2022;31(3):697–712.

15. Lidgren M, Wilking N, Jönsson B, Rehnberg C. Health related quality of life in different states of breast cancer. Qual Life Res. 2007;16(6):1073–81.

16. Yousefi M, Safari H, Akbari Sari A, Raei B, Ameri H. Assessing the performance of direct and indirect utility eliciting methods in patients with colorectal cancer: EQ-5D-5L versus C-TTO. Health Serv Outcomes Res Method. 2019;19(4):259–70.

17. Telford JJ, Levy AR, Sambrook JC, Zou D, Enns RA. The cost-effectiveness of screening for colorectal cancer. CMAJ : Canadian Medical Association Journal. 2010;182(12):1307.

18. Doherty MK, Leung Y, Su J, Naik H, Patel D, Eng L, et al. Health utility scores from EQ-5D and health-related quality of life in patients with esophageal cancer: a real-world cross-sectional study. Diseases of the Esophagus. 2018;31(12):doy058.

19. Herbert GL, Robinson DBT, Powell AG, Abdelrahman T, Khalid U, Lewis WG. Economic cost-utility analysis of stage-directed oesophageal cancer treatment. BJS Open. 2024 Mar 1;8(2):zrad159.

20. Chong CAKY, Gulamhussein A, Heathcote JE, Lilly L, Sherman M, Naglie G, et al. Health-State Utilities and Quality of Life in Hepatitis C Patients. Official journal of the American College of Gastroenterology | ACG. 2003;98(3):630.

21. Gruneau L, Kechagias S, Sandström P, Ekstedt M, Henriksson M. Cost-effectiveness analysis of noninvasive tests to identify advanced fibrosis in non-alcoholic fatty liver disease. Hepatol Commun. 2023;7(7):e00191.

22. Speight P, Palmer S, Moles D, Downer M, Smith D. The cost-effectiveness of screening for oral cancer in primary care. Health Technol Assess. 2006;(10(14)).

23. Tam VC, Ko YJ, Mittmann N, Cheung MC, Kumar K, Hassan S, et al. Cost-Effectiveness of Systemic Therapies for Metastatic Pancreatic Cancer. Current Oncology. 2013;20(2):90–106.

24. Peters MLB, Eckel A, Seguin CL, Davidi B, Howard DH, Knudsen AB, et al. Cost-Effectiveness Analysis of Screening for Pancreatic Cancer Among High-Risk Populations. JCO Oncol Pract. 2024;20(2):278–90.

25. Luengo-Fernandez R, Gray AM, Bull L, Welch S, Cuthbertson F, Rothwell PM. Quality of life after TIA and stroke. Neurology. 2013;81(18):1588–95.

26. Lyth J, Svennberg E, Bernfort L, Aronsson M, Frykman V, Al-Khalili F, et al. Cost-effectiveness of population screening for atrial fibrillation: the STROKESTOP study. European Heart Journal. 2023;44(3):196–204.

27. Nikolic E, Janzon M, Hauch O, Wallentin L, Henriksson M, for the PLATO Health Economic Substudy Group. Cost-effectiveness of treating acute coronary syndrome patients with ticagrelor for 12 months: results from the PLATO study. European Heart Journal. 2013;34(3):220–8.
