# Supplementary material for: Long-term outcomes of a digital alcohol intervention targeting online help-seekers: a simulation study of incidence of disease, quality-adjusted life-years and costs
Source: BMJ Public Health. 2026 Jun 24;4(2):e003503. doi: 10.1136/bmjph-2025-003503 (PMC13295776; doi:10.1136/bmjph-2025-003503)
Supplement: online supplemental file 2 [file bmjph-4-2-s002.docx]

# Appendix b - tables and figures

## scenario 1


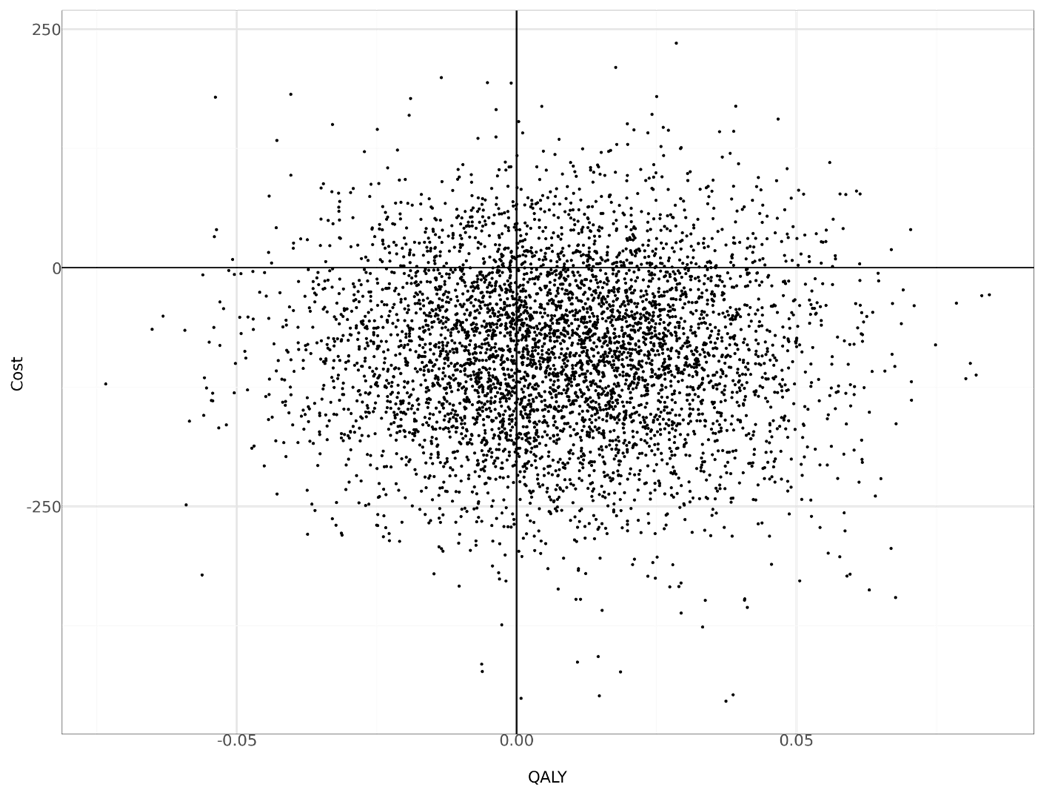


NE
9.8%

NW
5.5%

SE
53%

SW
31.7%

**Supplementary Figure 1 – Cost-effectiveness plane (Scenario 1). Incremental cost and incremental QALY (DAI minus TAU) from 5,000 simulations.**

## Scenario 2

**Supplementary Table 1 - Long-term simulation outcomes with an adjustment of the intervention’s population level effect (Scenario 2).**

| **Scenario 2: Intervention effect 10 percentage points annual decline** | | | | | | | | |
| --- | --- | --- | --- | --- | --- | --- | --- | --- |
|  | **Incidence rates, mean (IQR)^a^** | | | | | | **Hazard ratio (CI)^b^** | |
|  | **DAI** | | **TAU** | | | | **DAI vs. TAU** | |
| **Number of cases per 100 000 individuals:** | **Men** | **Women** | **Men** | | **Women** | | **Men** | **Women** |
| Alcohol-related liver disease | 1128  (658; 1416) | 933  (424; 1161) | 1145  (663; 1431) | | 950  (433; 1192) | | 0.985  (0.982; 0.987) | 0.982  (0.980; 0.985) |
| Breast cancer | 21  (14;29) | 3238  (3058; 3410) | 21  (14;29) | | 3245 (3058;3427) | | -- ^c^ | 0.999  (0.997; 1.000) |
| Colorectum cancer | 1537  (1450; 1616) | 1366  (1295; 1436) | 1535  (1445; 1616) | | 1368  (1298; 1436) | | 1.000  (1.000; 1.003) | 1.000  (0.998; 1.004) |
| Oesophageal cancer | 191  (152; 224) | 73  (55; 86) | 191  (152; 224) | | 73  (55; 86) | | 1.000  (0.995; 1.006) | 0.997  (0.989; 1.005) |
| Haemorrhagic stroke | 1201  (1150; 1254) | 947  (906; 989) | 1203  (1150; 1254) | | 947  (946; 949) | | 0.998  (0.996; 1.001) | 0.998  (0.995; 1.000) |
| Ischemic stroke | 62602  (6139; 6415) | 5058  (5054; 5062) | 6275  (6139; 6415) | | 5060  (4956; 5162) | | 0.998  (0.997; 0.999) | 0.998  (0.998; 1.000) |
| Liver cancer | 663  (657; 670) | 400  (394; 406) | 671  (496; 806) | | 406  (265; 482) | | 0.992  (0.988; 0.995) | 0.983  (0.979; 0.986) |
| Myocardial infarction | 4915  (4808; 5022) | 3153  (3083; 3224) | 4910  (4808; 5012) | | 3151  (3079; 3224) | | 1.000  (0.999; 1.002) | 1.001  (0.999; 1.002) |
| Oral cancer | 326  (272; 372) | 184  (155; 210) | 329  (277; 372) | | 184  (155; 210) | | 0.993  (0.998; 0.997) | 0.999  (0.994; 1.004) |
| Pancreas cancer | 288  (258; 315) | 294  (265; 320) | 287  (258; 315) | | 295  (265; 320) | | 1.00  (0.997; 1.007) | 0.999  (0.995; 1.003) |
|  | **Cost-effectiveness outcomes (per individual), mean (IQR)^a^** | | | | | | | |
|  | **DAI** | | | **TAU** | | **Difference** | | |
| Health care costs (euro) | 3053  (2933; 3142) | | | 3065  (2947; 3155) | | -13  (-70; 45) | | |
| QALY | 19.667  (19.650; 19.685) | | | 19.665  (19.648; 19.683) | | 0.002  (-0.013; 0.017) | | |
| ICER (euro per QALY) | DAI dominant (improved QALYs and lower costs) | | | | | | | |
| ^a^ = IQR represents the uncertainty across simulations ^b^ = Confidence interval, mean plus/minus 1.96 standard errors ^c^ = Estimating hazard ratio was not feasible due to an insufficient number of cases of breast cancer among men | | | | | | | | |


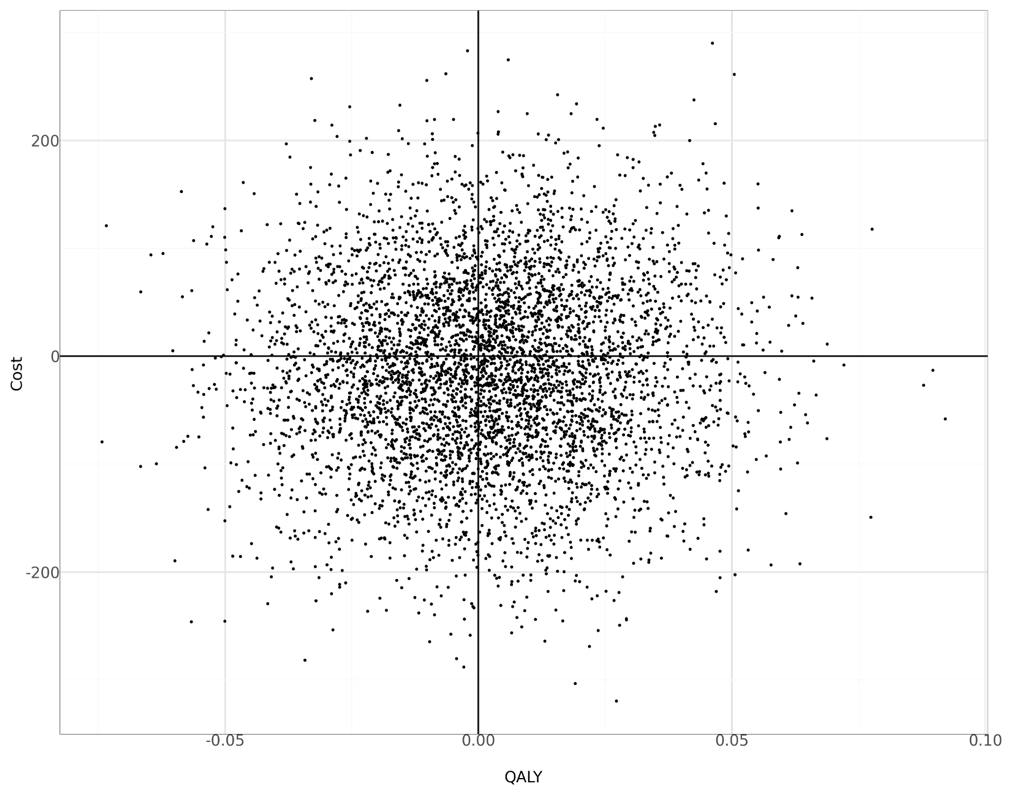


NW
19.7%

NE
24%

SW
26.7%

SE
29.6%

**Supplementary Figure 2 – Cost-effectiveness plane (Scenario 2). Incremental cost and incremental QALY (DAI minus TAU) from 5,000 simulations.**

## scenario 3

**Supplementary Table 2 - Long-term simulation outcomes with an adjustment of the intervention’s population level effect (Scenario 3).**

| **Scenario 3: Intervention effect 1 percentage points annual decline** | | | | | | | |
| --- | --- | --- | --- | --- | --- | --- | --- |
|  | **Incidence rates (IQR)^a^** | | | | | **Hazard ratio (CI)^b^** | |
|  | **DAI** | | **TAU** | | | **DAI vs. TAU** | |
| **Number of cases per 100 000 individuals:** | **Men** | **Women** | **Men** | | **Women** | **Men** | **Women** |
| Alcohol-related liver disease | 1000  (572; 1254) | 782  (351; 975) | 1145  (662; 1431) | | 950  (434; 1192) | 0.873  (0.870; 0.875) | 0.823  (0.821; 0.825) |
| Breast cancer | 21  (14; 29) | 3208  (3024; 3389) | 21  (14; 28) | | 3246  (3058; 3427) | --^c^ | 0.988  (0.987; 0.989) |
| Colorectum cancer | 1518  (1436; 1602) | 1349  (1278; 1416) | 1535  (1445; 1616) | | 1368  (1298; 1436) | 0.988  (0.986; 0.990) | 0.986  (0.984; 0.988) |
| Oesophageal cancer | 184  (147; 219) | 71  (55; 86) | 191  (153; 224) | | 73  (55; 86) | 0.967  (0.963; 0.975) | 0.978  (0.969; 0.986) |
| Haemorrhagic stroke | 1138  (1087; 1188) | 912  (911; 914) | 1203  (1150; 1254) | | 947  (906; 989) | 0.946  (0.944; 0.949) | 0.963  (0.960; 0.965) |
| Ischemic stroke | 6030  (5900; 6158) | 4926  (4818; 5028) | 6276  (6138; 6415) | | 5060  (4956; 5163) | 0.962  (0.961; 0.963) | 0.974  (0.973; 0.975) |
| Liver cancer | 613  (452; 739) | 357  (238; 424) | 671  (406; 265) | | 406  (265; 482) | 0.915  (0.912; 0.918) | 0.878  (0.875; 0.882) |
| Myocardial infarction | 4990  (4884; 5099) | 3192  (3189; 3195) | 4910  (4807; 5013) | | 3151  (3079; 3224) | 1.016  (1.014; 1.017) | 1.012  (1.011; 1.014) |
| Oral cancer | 311  (262; 353) | 177  (151; 203) | 329  (277; 372) | | 184  (155; 210) | 0.945  (0.943; 0.953) | 0.963  (0.958; 0.968) |
| Pancreas cancer | 289  (258; 315) | 294  (269; 320) | 288  (258; 315) | | 295  (265; 320) | 1.004  (0.999; 1.009) | 0.999  (0.995; 1.004) |
|  | **Cost-effectiveness outcomes (per individual), mean (IQR)^a^** | | | | | | |
|  | **DAI** | | | **TAU** | | **Difference** | |
| Health care costs (euro) | 2994  (2888; 3076) | | | 3066  (2947; 3155) | | -71  (-128; -14) | |
| QALY | 19.671  (19.655; 19.690) | | | 19.665  (19.648; 19.690) | | 0.007  (-0.009; 0.021) | |
| ICER (euro per QALY) | DAI dominant (improved QALYs and lower costs) | | | | | | |
| ^a^ = IQR represents the uncertainty across simulations ^b^ = Confidence interval, mean plus/minus 1.96 standard errors ^c^ = Estimating hazard ratio was not feasible due to an insufficient number of cases of breast cancer among men | | | | | | | |


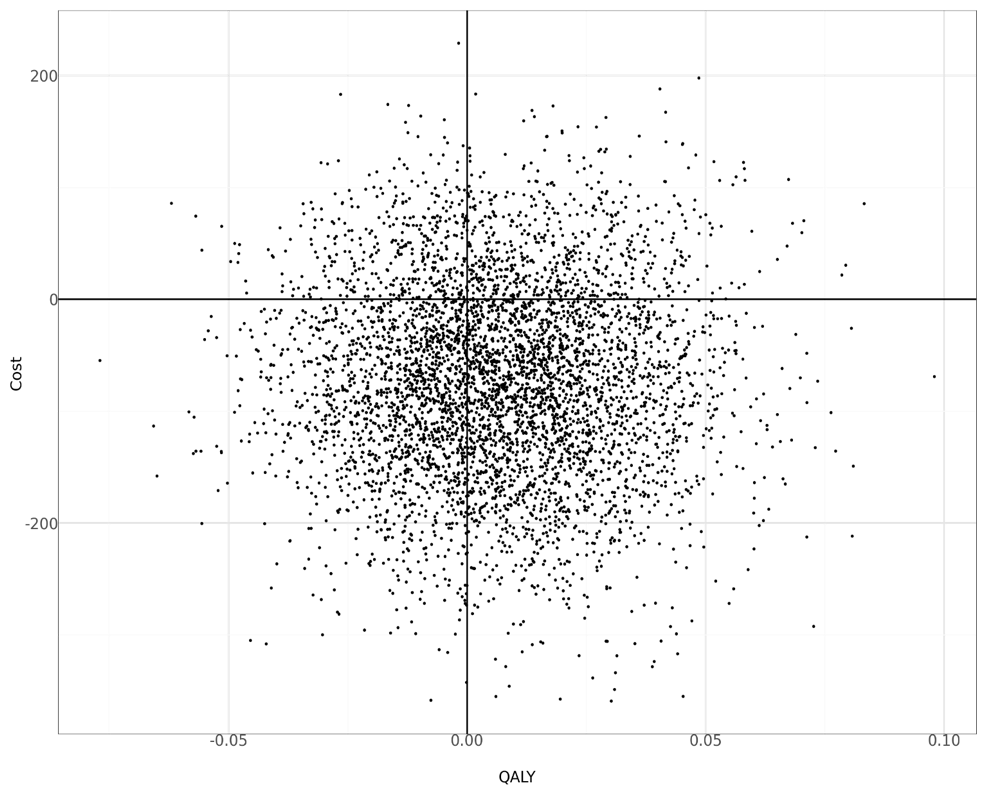


SW
31.2%

SE
48.6%

NW
8.1%

NE
12.1%

**Supplementary Figure 3 – Cost-effectiveness plane (Scenario 3). Incremental cost and incremental QALY (DAI minus TAU) from 5,000 simulations.**

## Scenario 4

**Supplementary Table 3 - Long-term simulation outcomes with halving of the intervention’s population level effect (Scenario 4).**

| **Scenario 4: Intervention effect halved** | | | | | | | |
| --- | --- | --- | --- | --- | --- | --- | --- |
|  | **Incidence rates (IQR)^a^** | | | | | **Hazard ratio (CI)^b^** | |
|  | **DAI** | | **TAU** | | | **DAI vs. TAU** | |
| **Number of cases per 100 000 individuals:** | **Men** | **Women** | **Men** | | **Women** | **Men** | **Women** |
| Alcohol-related liver disease | 1051  (601; 1321) | 837  (375; 1038) | 1145  (662; 1431) | | 950  (434; 1192) | 0.915  (0.913; 0.917) | 0.873  (0.871; 0.875) |
| Breast cancer | 21  (14; 28) | 3235  (3052; 3413) | 21  (14; 28) | | 3246  (3058; 3427) | --^c^ | 0.993  (0.992; 0.994) |
| Colorectum cancer | 1533  (1445; 1612) | 1369  (1298; 1440) | 1535  (1445; 1616) | | 1368  (1298; 1436) | 0.991  (0.988; 0.993) | 0.991  (0.989; 0.993) |
| Oesophageal cancer | 188  (148; 219) | 73  (55; 86) | 191  (153; 224) | | 73  (55; 86) | 0.981  (0.975; 0.987) | 0.984  (0.976; 0.993) |
| Haemorrhagic stroke | 1168  (1116; 1221) | 938  (899; 978) | 1203  (1150; 1254) | | 947  (906; 989) | 0.959  (0.957; 0.962) | 0.970  (0.968; 0.972) |
| Ischemic stroke | 6174  (6038; 6305) | 5055  (4946; 5159) | 6276  (6138; 6415) | | 5060  (4956; 5163) | 0.970  (0.969; 0.971) | 0.979  (0.978; 0.980) |
| Liver cancer | 652  (477; 779) | 386  (255; 465) | 671  (406; 265) | | 406  (265; 482) | 0.942  (0.939; 0.945) | 0.915  (0.912; 0.918) |
| Myocardial infarction | 5022  (4917; 5127) | 3238  (3165; 3309) | 4910  (4807; 5013) | | 3151  (3079; 3224) | 1.012  (1.011; 1.014) | 1.010  (1.009; 1.011) |
| Oral cancer | 319  (267; 362) | 181  (152; 207) | 329  (277; 372) | | 184  (155; 210) | 0.966  (0.961; 0.970) | 0.975  (0.969; 0.980) |
| Pancreas cancer | 290  (262; 320) | 298  (269; 324) | 288  (258; 315) | | 295  (265; 320) | 0.999  (0.994; 1.004) | 1.004  (1.000; 1.008) |
|  | **Cost-effectiveness outcomes (per individual), mean (IQR)^a^** | | | | | | |
|  | **DAI** | | | **TAU** | | **Difference** | |
| Health care costs (euro) | 3016 (2888; 3144) | | | 3066  (2947; 3155) | | -50  (-124; -24) | |
| QALY | 19.668 (19.642; 19.693) | | | 19.665  (19.648; 19.690) | | 0.003  (-0.012; 0.017) | |
| ICER (euro per QALY) | DAI dominant (improved QALYs and lower costs) | | | | | | |
| ^a^ = IQR represents the uncertainty across simulations ^b^ = Confidence interval, mean plus/minus 1.96 standard errors ^c^ = Estimating hazard ratio was not feasible due to an insufficient number of cases of breast cancer among men | | | | | | | |


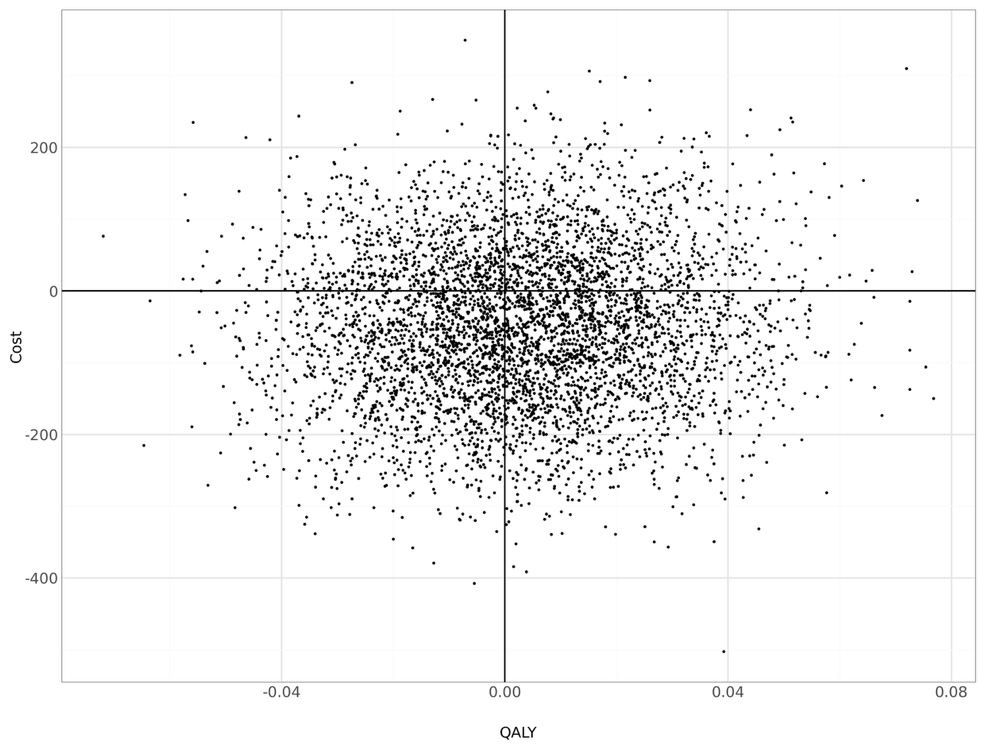


SW
30.4%

SE
37.1%

NW
14.4%

NE
18.1%

**Supplementary Figure 4 – Cost-effectiveness plane (Scenario 4). Incremental cost and incremental QALY (DAI minus TAU) from 5,000 simulations.**
