# Supplementary material for: Long-term outcomes of a digital alcohol intervention targeting online help-seekers: a simulation study of incidence of disease, quality-adjusted life-years and costs
Source: BMJ Public Health. 2026 Jun 24;4(2):e003503. doi: 10.1136/bmjph-2025-003503 (PMC13295776; doi:10.1136/bmjph-2025-003503)

Supplementary Figure 1 - Cumulative incidence rates per 100 000 individuals of the modelled diseases (mean and IQRrepresenting the uncertainty across simulations)

Panel A – Alcohol-related liver disease

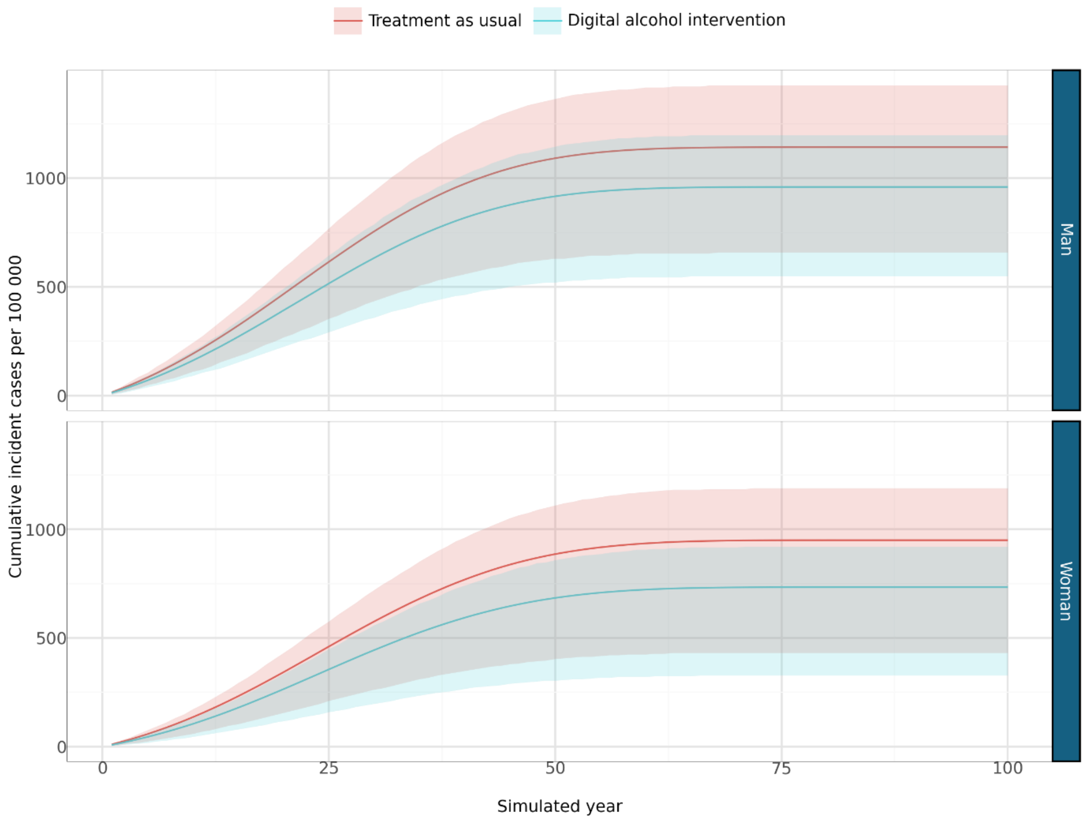

Panel B – Breast cancer

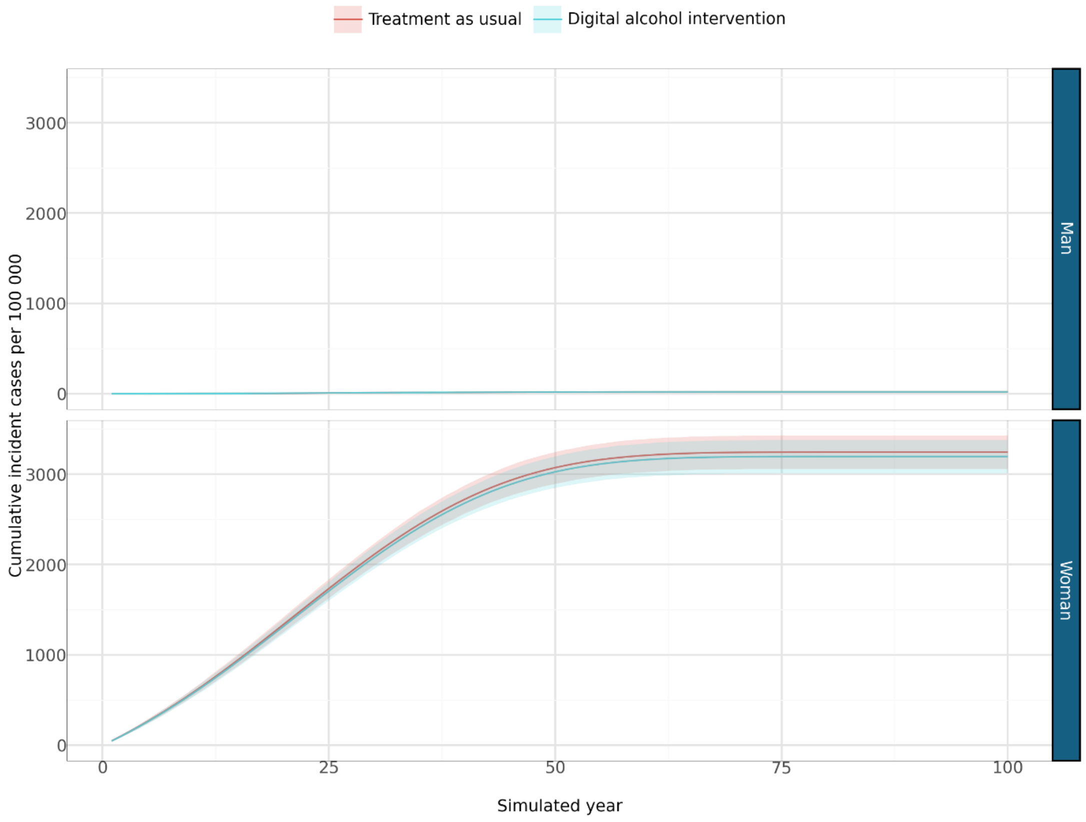

Panel C – Colorectum cancer

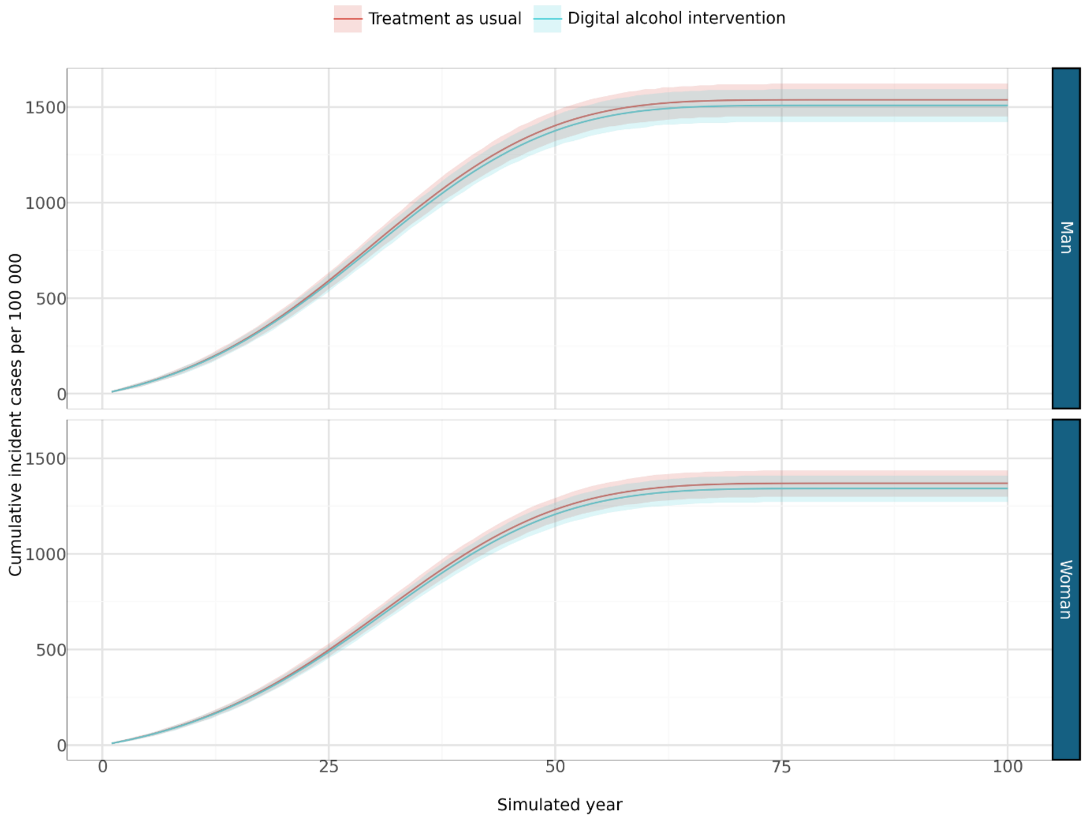

Panel D – Oesophageal cancer

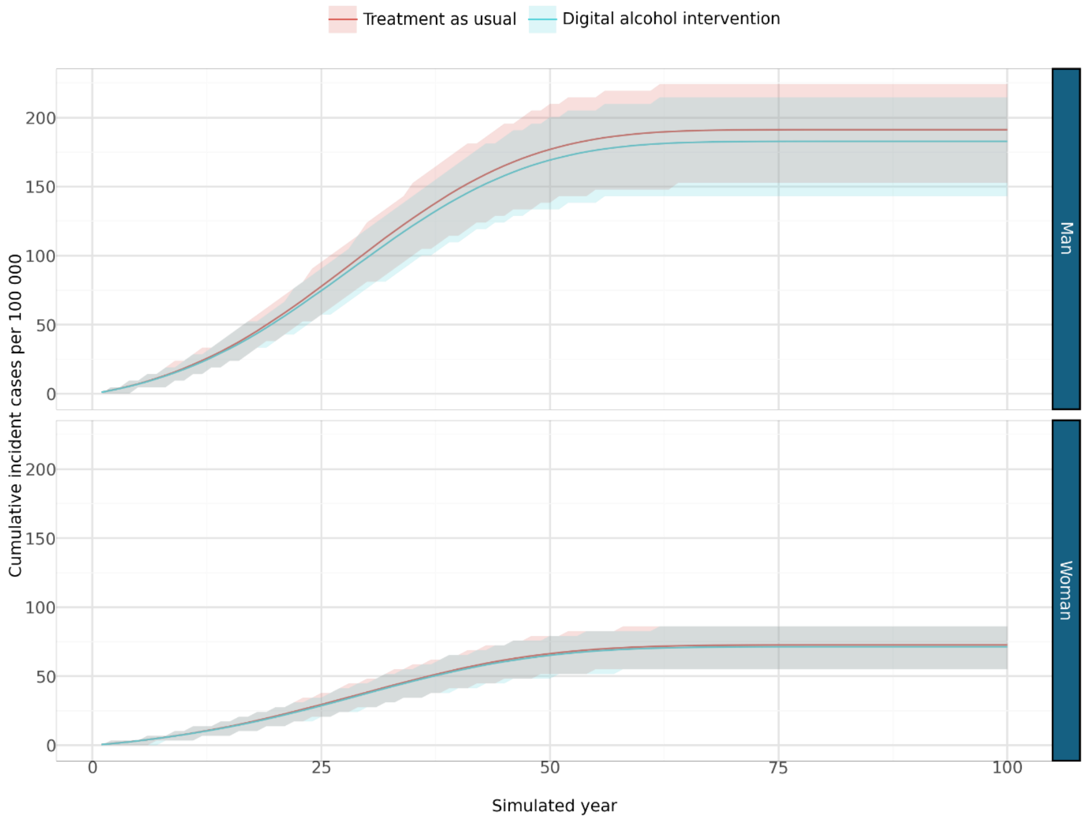

Panel E – Liver cancer

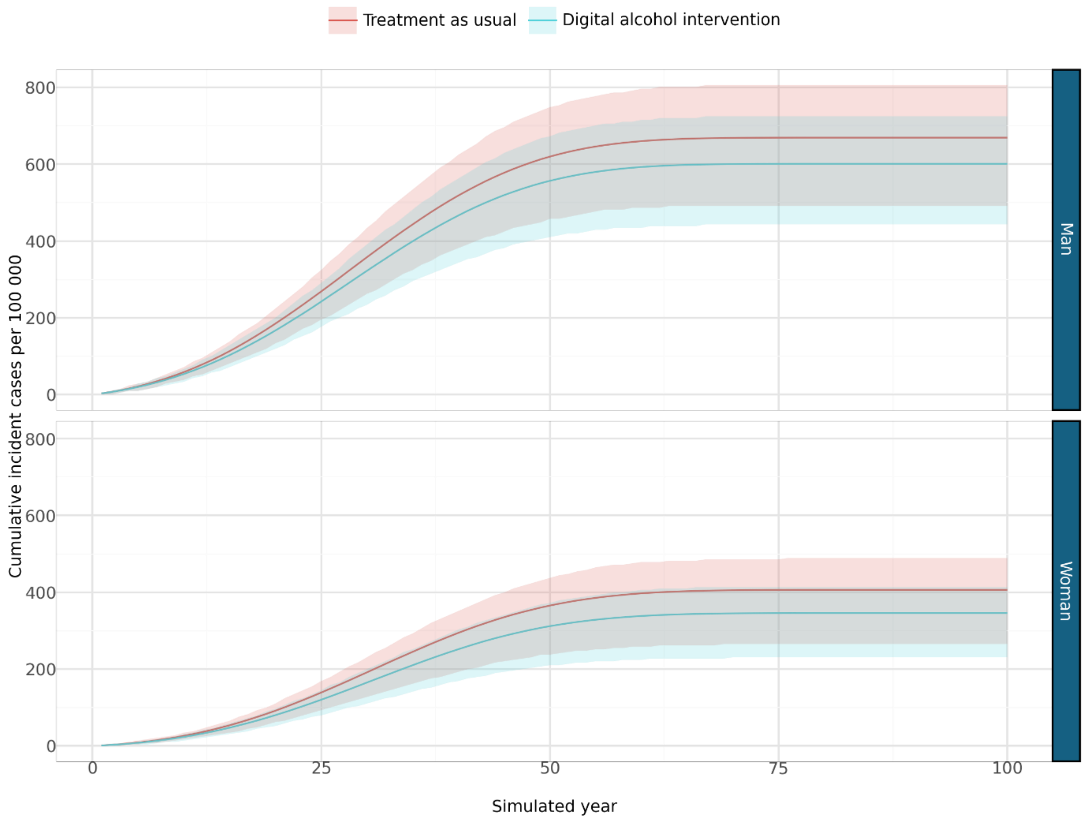

Panel F– Oral cancer

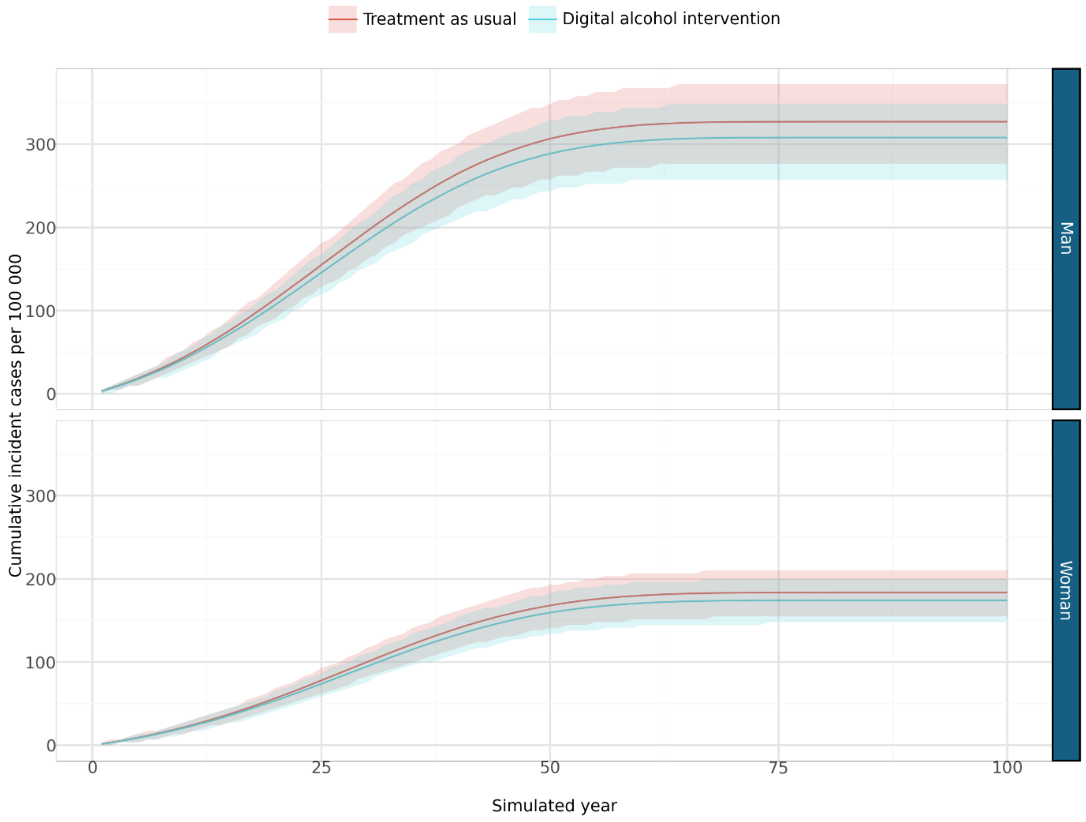

Panel G – Pancreas cancer

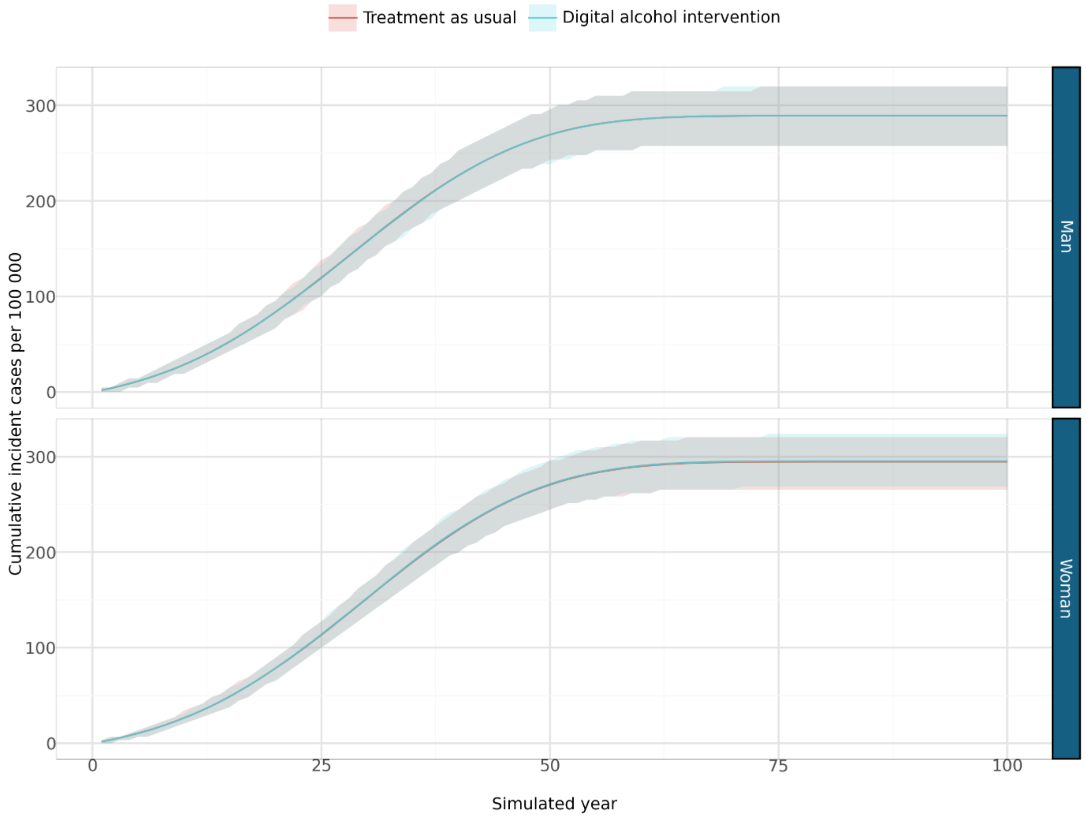

Panel H – Haemorrhagic stroke

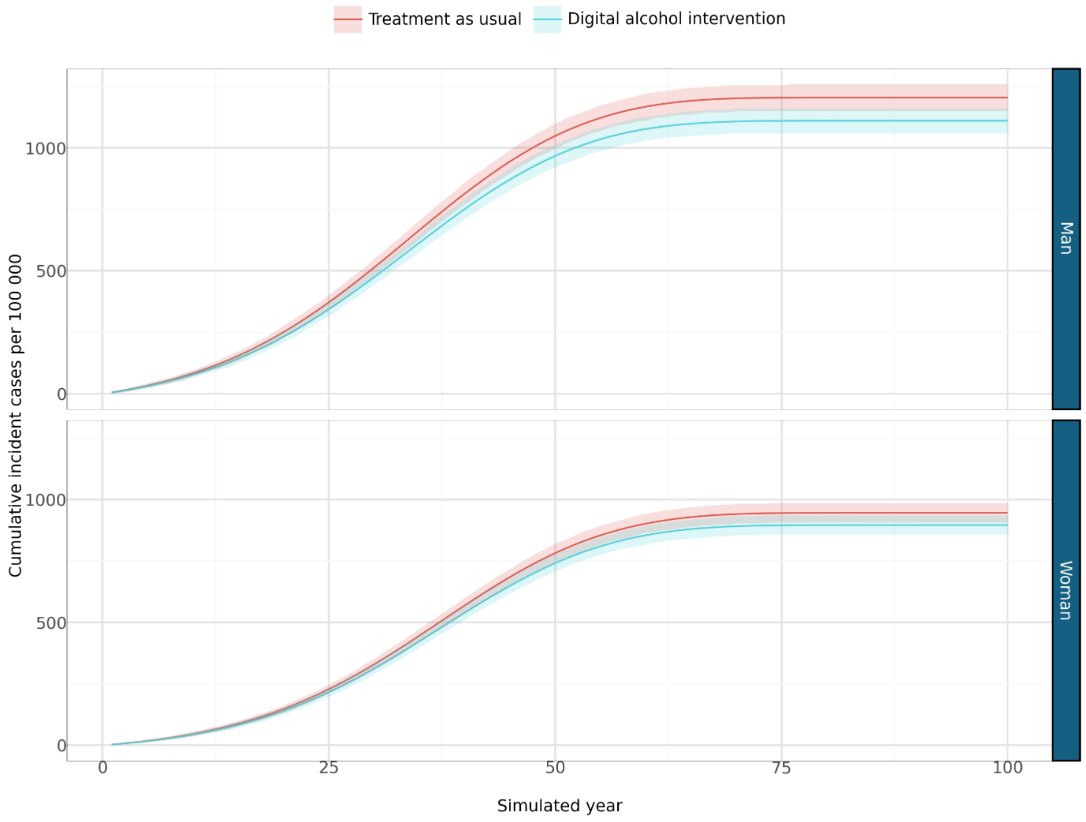

Panel I– Ischemic stroke

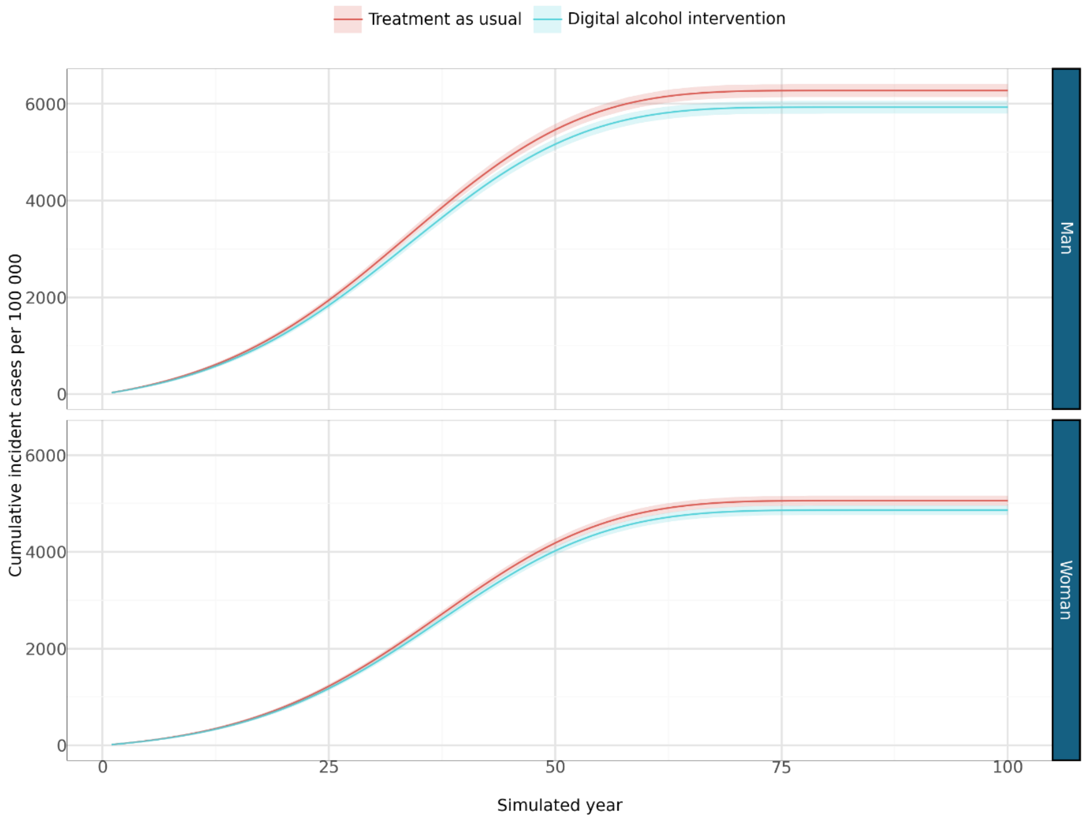

Panel J – Myocardial infarction

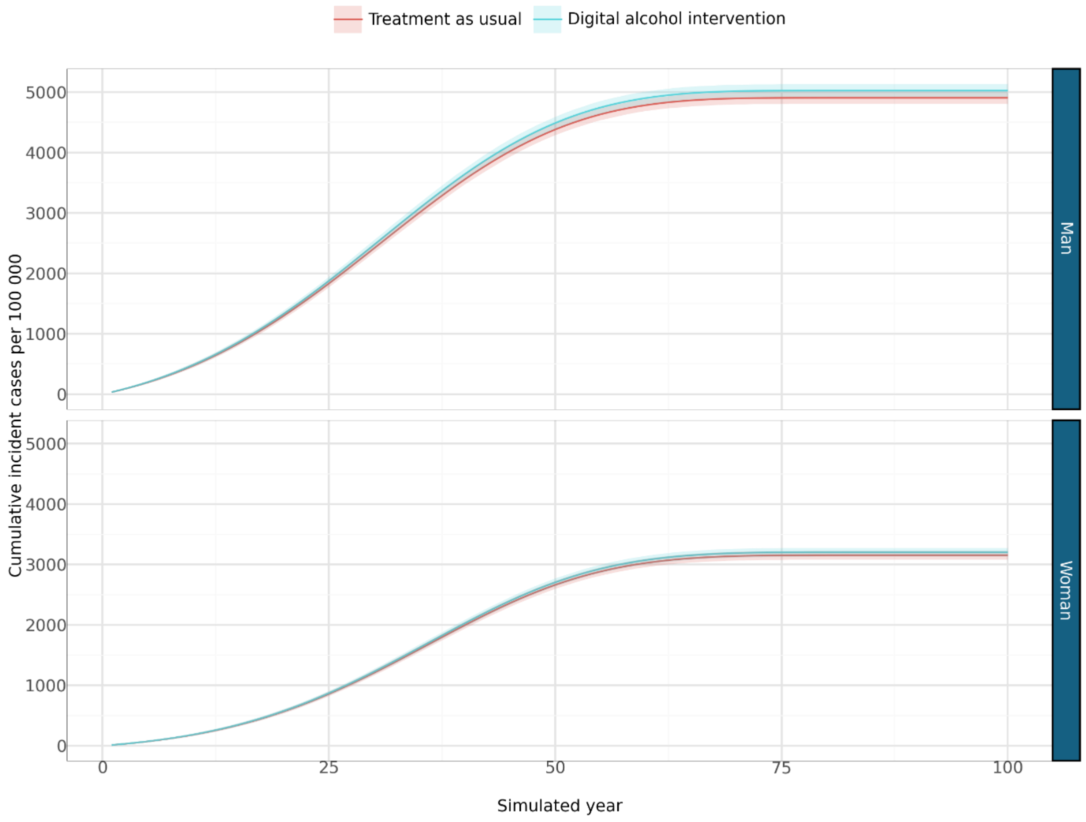

Supplement: online supplemental file 3 [file bmjph-4-2-s003.pdf]
